# Supplementary material for: In-situ noncovalent interaction of ammonium ion enabled C–H bond functionalization of polyethylene glycols
Source: Nat Commun. 2024 May 24;15:4445. doi: 10.1038/s41467-024-48584-8 (PMC11126569; doi:10.1038/s41467-024-48584-8)
Supplement: Supplementary file 1 — Supplementary Information [file 41467_2024_48584_MOESM1_ESM.pdf]

Supplementary Information

In-situ Noncovalent Interaction of Ammonium Ion Enabled C–H Bond Functionalization of Polyethylene Glycols

Zongnan Zhang,[1†] Xueli Lv,[2†] Xin Mu,[1] Mengyao Zhao,[3] Sichang Wang,[3] Congyu Ke,[3] Shujiang Ding,*[1] Dezhong Zhou,*[1] Minyan Wang,*[2] Rong Zeng*[1]

[1] School of Chemistry & School of Chemical Engineering and Technology, Xi'an Jiaotong University, Xi'an 710049, P. R. China

[2] State Key Laboratory of Coordination Chemistry, School of Chemistry and Chemical Engineering, Nanjing University, Nanjing 210093, P. R. China

[3] College of Chemistry and Chemical Engineering, Xi'an Shiyou University, Xi'an, 710065, P. R. China

Corresponding author: dingsj@xjtu.edu.cn; dezhong.zhou@xjtu.edu.cn; wangmy@nju.edu.cn; rongzeng@xjtu.edu.cn

†These authors contributed equally to this work

Table of Contents

**Supplementary Notes**	S3-S4
**Supplementary methods and Discussions**	S5-S86
(1) Preparation of Starting materials	S5-S6
(2) Screening of the reaction of PEG 2000 and **1**	S7
(3) The time-dependent and kinetic experiments of PEG 2000 with **1**	S8-S12
(4) The GPC traces and MALDI-TOF spectra using PEG 2000 with **2**.	S13-S14
(5) Concern on the oligomerization	S15
(6) The C–H bond functionalization of the PEGs with various deficient alkenes	S16-S25
(7) PEGylation of pharmaceuticals with PEG 12000	S26-S33
(8) Modular synthesis of multifunctional polymeric carrier template	S34-S35
(9) PEGylation of BSA with **MeO-PEG-OMe 2000-17**	S36-S38
(10) Mechanism investigation of C–H bond functionalization of PEGs with acylate	S39-S52
(11) Computational details	S53
(12) Spectra	S54-S86
**Supplementary References**	S87-S88

Supplementary Notes

(1) Materials:

Acetonitrile (MeCN) and dichloromethane (DCM) were purified by a Vigor solvent purification system. Anhydrous $FeCl_3$ compounds was purchased from Energy Chemical. TBACl (tetrabutylammonium chloride) was purchased from TCI. $FeCl_3$ and TBACl were stored and weighed in the glovebox. Iridium salt and quinuclidine were purchased from Adamas-beta. PEG 2000 and MeO-PEG-OMe 2000 were purchased from Aladdin. PEG 12000 was purchased from Sigma-Aldrich. Other commercially available chemicals were purchased and used without additional purification unless noted otherwise.

(2) Setting-up of the photo-induced reactions:

The LED light (30 W, emitting area: 30 × 30 mm) was assembled using the 460 nm chips purchased from GuangHong Chips. The material of the reaction vessels is regular borosilicate glass. The distance from the light source to the reaction vessel is 5 cm (**Supplementary Figure S1**).

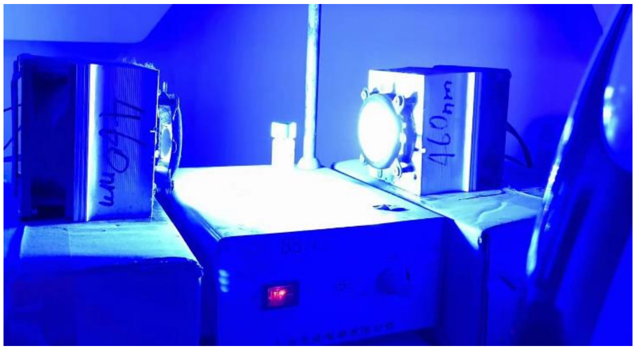

Supplementary Figure 1. The setting-up reactions.

(3) IR:

Infrared spectra were recorded on a Nicolet iS5 using neat thin film technique.

(4) NMR:

^{1}H NMR spectra were recorded on JNM-ECZ400S/L1 or Bruker-600 MHz spectrometer at 400 or 600 MHz, ^{13}C NMR spectra were recorded at 101 or 151 MHz, ^{19}F NMR spectra were recorded at 376 MHz, and ^{31}P NMR spectra were recorded at 162 MHz. Spectra were acquired in $CDCl_3$. Chemical shifts are reported in parts per million (ppm, δ), downfield from tetramethylsilane (TMS, δ = 0.00 ppm) and are referenced to residual solvent ($CDCl_3$, δ = 7.26 ppm (^{1}H) and 77.00 ppm (^{13}C). Coupling constants were reported in Hertz (Hz). Data for NMR

spectra were reported as follows: s = singlet, d = doublet, t = triplet, dd = doublet of doublets, m = multiplet, coupling constant (Hz), and integration.

(5) HRMS:

High-resolution mass spectra (HRMS) were obtained on a Waters I-Class VION IMS QTof and are reported as m/z (relative intensity). Accurate masses are reported for the molecular ion $[M+Na]^+$, $[M+H]^+$, $[M-OH]^+$, $[M-H]^-$ or $[M]^+$.

(6) GPC:

M_w, M_n and *Đ* of polymers were determined by an Agilent 1260 infinity II gel permeation chromatography (GPC) equipped with a refractive index (RI) detector. 5.0 mg of sample was dissolved in 1 mL of DMF containing 0.1% LiBr, and then filtered through a 0.22 μm filter. GPC columns (PolarGel-M Gard, 50 mm × 7.5 mm, and PolarGel-M, 300 mm × 7.5 mm, two in series) were eluted with DMF (plus 0.1% LiBr) at a flow rate of 1 mL/min at 50 °C. GPC column were calibrated with linear poly(methyl methacrylate) (PMMA) standards.

(7) MALDI-TOF MS:

MALDI-TOF mass spectra were acquired on an Autoflex Speed MALDI-TOF mass spectrometer (Bruker Daltonics, Germany) equipped with a Smart beam-II laser (355 nm, 1 KHz, Bruker Daltonics). Take an appropriate amount of sample and dissolve it with 50% acetonitrile aqueous solution (0.1% TFA). Matrix: *trans*-2-[3-(4-tert-butylphenyl)-2-methyl-2-propenylidene] malononitrile (DCTB) was dissolved in DCM (20 mg/mL), and sodium trifluoroacetate was dissolved in methanol (10 mg/mL). The DCTB solution and the sodium trifluoroacetate solution were mixed in ratio (v/v) of 10/1. Taking 0.5 μL of matrix solution on the target plate, after the solution is dry, take another 0.5 μL of sample solution in the same position, after the solution is naturally dried, taking another 0.5 μL of matrix to spread on the upper layer of the sample. The MS scan range of the sample was 1000-20000 Da.

(8) Definition of the level of functionalization (LOF)

The level of functionalization (LOF) = $n_{(\text{benched alkene in polymer})}/n_{(\text{monomer of polymer})} = A_{(\text{benched alkene in polymer})}/A_{(\text{monomer of polymer})}$. n: mole number; A: integral area by ^{1}H NMR.

Supplementary methods and Discussions

(1) Preparation of Starting materials

The electron deficient alkenes including **12**[1], **14**[2] are known and were prepared according to the known literature.

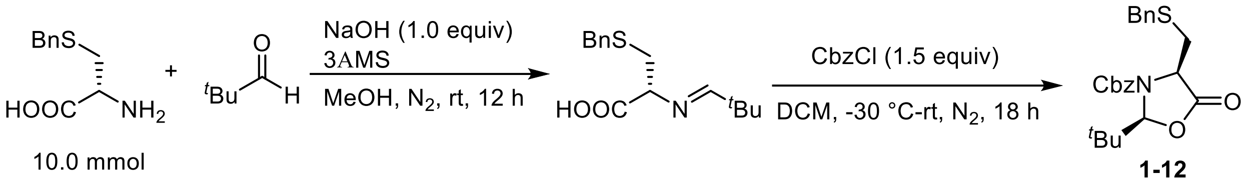

To a round bottom flask equipped with a stir bar was added S-benzyl-L-cysteine (2.1100 g, 1.0 mmol), NaOH (0.4000 g, 10.0 mmol), and anhydrous MeOH (80 mL). The reaction was stirred at room temperature for 30 minutes. Trimethylacetaldehyde (1.3 mL, d = 0.793 g/mL, 1.0320 g, 12.0 mmol) and activated 3 Å molecular sieves (20.0000 g) were added to the reaction flask in one portion. The reaction was placed under nitrogen atmosphere and stirred at room temperature until the starting material had been consumed. The reaction was quickly filtered through celite and concentrated by rotary evaporation. The residue was dried under high vacuum for 24 hours to afford the imine as a white solid. The imine was dissolved in anhydrous DCM (80 mL) and cooled to -30 °C. Benzyl chloroformate (2.1 mL, d = 1.212 g/mL, 2.5500 g, 15.0 mmol) was added to the reaction dropwise via syringe. The reaction then warmed to room temperature and stirred for 18 hours. The mixture was washed with 1 M aqueous NaOH (50 mL × 1). The organic layer was dried over sodium sulfate, filtered, and concentrated by rotary evaporation. The residue was purified by flash chromatography (petroleum ether/ethyl acetate = 10/1) to afford the product **1-12** (700.4 g, 20% yield) as a colorless oil. The physical properties and spectral data were consistent with the reported values[1].

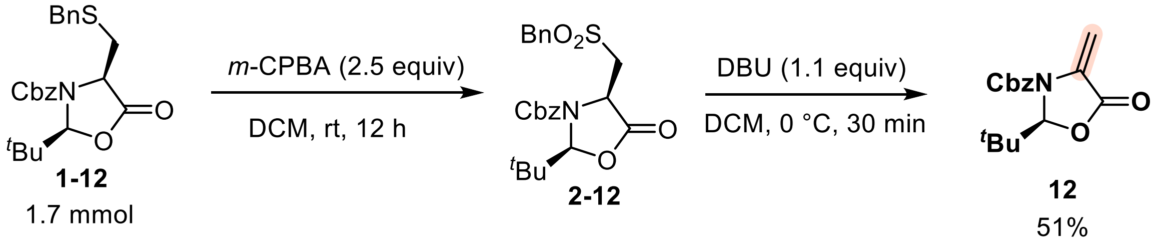

To a round bottom flask equipped with a stir bar was added **1-12** (700.0 mg, 1.7 mmol), *meta*-chloroperoxybenzoic acid (1.0627 g, 4.3 mmol), and DCM (20 mL). The reaction was stirred at room temperature for 18 hours. The reaction mixture was washed with 1 M aqueous sodium

hydroxide (50 mL × 3). The organic layer was dried over sodium sulfate, filtered, and concentrated by rotary evaporation. The residue was purified by flash chromatography (petroleum ether/ethyl acetate = 10/1 to 5/1) to afford the product **2-12** directly for further conversion.

To a round bottom flask equipped with a stir bar was added **2-12**, and DCM (20 mL).The flask was chilled to 0 °C in an ice bath, and DBU (0.3 mL, d = 1.019 g/mL, 288.8 mg, 1.9 mmol) was added dropwise via syringe. The reaction was stirred at 0 °C until the starting material had been consumed (determined by TLC, about 10 minutes). While still at 0 °C, the reaction mixture was quenched with saturated aqueous ammonium chloride (20 mL), the layers were separated, and the organic phase was washed with saturated aqueous ammonium chloride (50 mL × 3). The organic layer was dried over sodium sulfate, filtered, and concentrated by rotary evaporation. The residue was purified by flash chromatography (petroleum ether/ethyl acetate = 10/1) to afford the product **12** (250.9 g, 51%) as a white solid. The physical properties and spectral data are consistent with the reported values[1].

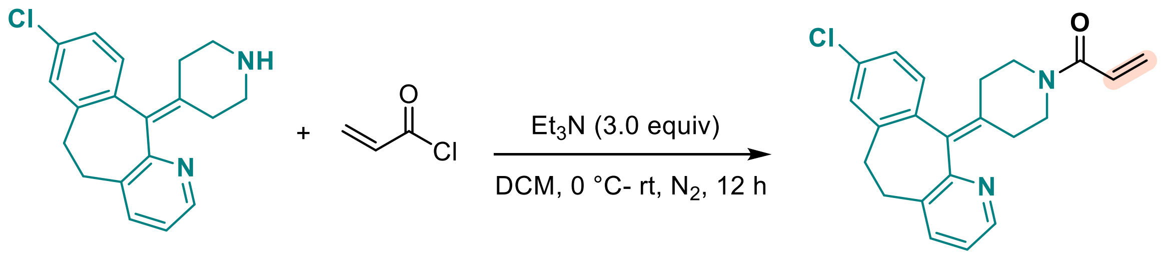

2.0 mmol

Desloratadine

1.2 equiv

14 96%

from Desloratadine

To an oven-dried 50 mL round-bottom flask were added Desloratadine (622.0 mg, 2.0 mmol), acryloyl chloride (0.2 mL, d = 1.114 g/mL, 218.4 mg, 2.4 mmol) and DCM (20 mL) under N_2. Then Et_3N (0.6 mL, d = 0.728 g/mL, 404.0 mg, 4.0 mmol) was added under a nitrogen atmosphere at 0 °C. The resulting mixture was warmed up to room temperature and stirred for 12 h until the reaction was complete as monitored by TLC. To this reaction vessel was added a saturated aqueous solution of Na_2CO_3 (10 mL), and the aqueous layer was extracted with ethyl acetate (EA) (30 mL × 3) and washed with a saturated aqueous solution of NaCl. The combined organic layer was dried over anhydrous Na_2SO_4, filtered, and evaporated. Silica gel flash chromatography (petroleum ether/ethyl acetate = 1/3) afforded **14** (701.6 mg, 96%). The physical properties and spectral data are consistent with the reported values[2].

(2) Screening of the reaction of PEG 2000 and 1

Supplementary Table 1 Reaction optimization using PEG 2000 and **1** [a]

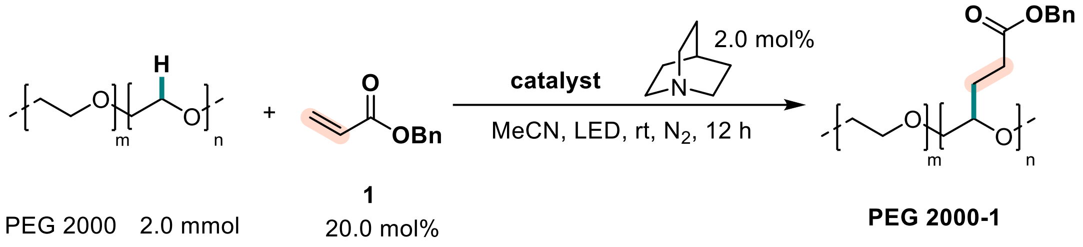

Entry	Catalyst	solvent	LOF (mol%)
1	**PC-1** (0.1 mol%)	MeCN	8.3
2	none	MeCN	0
3[b]	**PC-1** (0.1 mol%)	MeCN	0
4	**PC-2** (0.1 mol%)	MeCN	0
5	**PC-3** (0.1 mol%)	MeCN	6.0
6	**PC-4** (0.1 mol%)	MeCN	trace
7	4CzIPN (0.1 mol%)	MeCN	trace
8	TBAFeCl4 (0.2 mol%)	MeCN	0[b]
9[b]	TBAFeCl4 (0.2 mol%)	MeCN	0[b]
10	Benzophenone (0.2 mol%)	MeCN	0[b]
11	**PC-1** (0.1 mol%)	DCM	trace
12	**PC-1** (0.1 mol%)	acetone	7.5
13	**PC-1** (0.1 mol%)	DMF	0

[a] The reactions were conducted with PEG 2000 (4.0 mmol), **1** (20.0 mol%), catalyst, quinuclidine (2 mol%), and MeCN (4 mL) in a 4 mL vial with 460 nm LED photo-irradiation at rt for 12 h. [b] No quinuclidine was used. [c] 390 nm LEDs (100 W) were used.

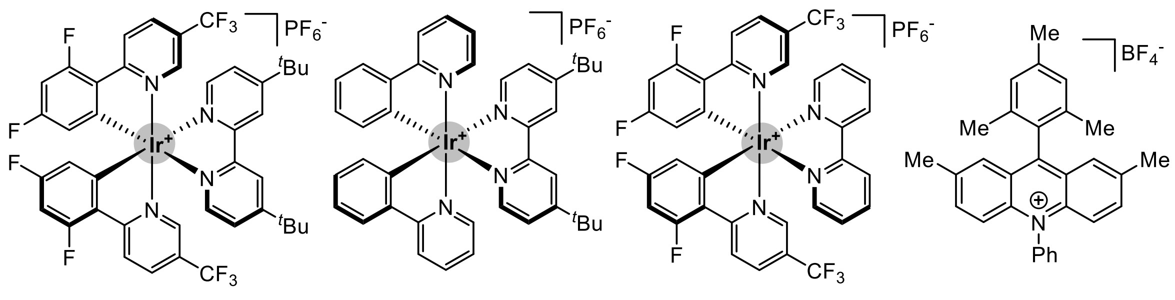

(3) The time-dependent and kinetic experiments of PEG 2000 with 1

(3-1) The time-dependent experiment

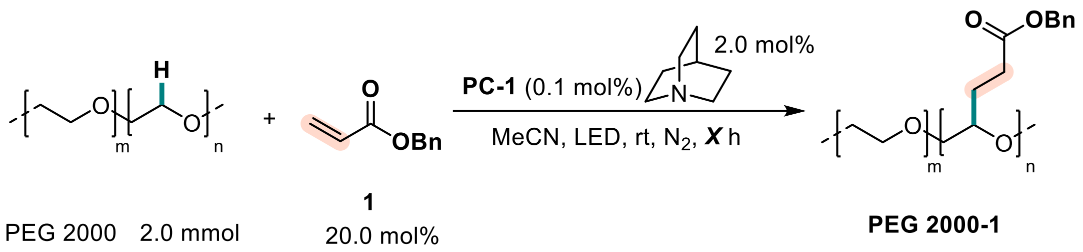

To a 4 mL vial were added **PC-1** (2.2 mg, 0.002 mmol), quinuclidine (4.4 mg, 0.04 mmol), PEG 2000 (88.0 mg, 2.0 mmol), **1** (60.0 µL, *d* = 1.080 g/mL, 64.8 mg, 0.4 mmol), and MeCN (4.0 mL) in an N_2 glovebox. The vial was then sealed and transferred out of the glovebox. Under irradiation at 460 nm LEDs, the resulting mixture was stirred for 1, 2, 5, 12, 24, or 48 hours at rt. Evaporation and Flash chromatography on silica gel (DCM to DCM/MeOH = 10/1) afforded **PEG 2000-1**. The level of functionalization was determined by ^{1}H NMR.

Time (h)	LOF (mol%)
0	0
1	0.8
2	1.4
5	5.5
12	8.3
24	12.5
48	17.6

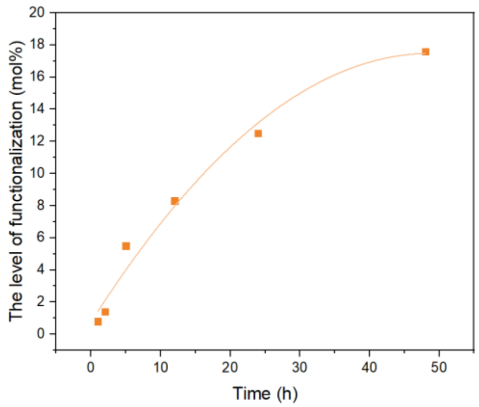

Supplementary Figure 2. LOF vs reaction time.

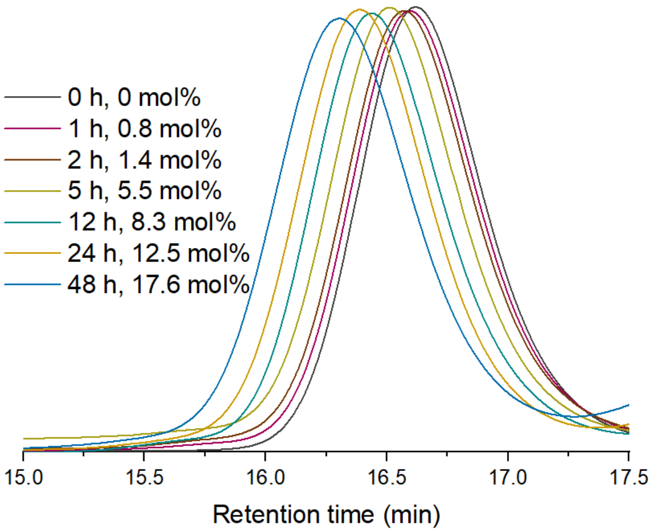

Time (h)	LOF (mol%)	M_w (g/mol)	M_n (g/mol)	*Đ*
0	0	3213	2913	1.10
1	0.8	3286	2971	1.11
2	1.4	3386	3065	1.10
5	5.5	3675	3425	1.07
12	8.3	3896	3607	1.08
24	12.5	4157	3861	1.08
48	17.6	4524	4164	1.09

Supplementary Figure 3. GPCs of **PEG 2000-1** in various times

(3-2) Reaction kinetics

The kinetic orders of the decarboxylative methylation reaction in large-alkyl carboxylic acid, thioxanthone photosensitizer, nickel catalyst, and light intensity were determined by the method of Variable Time Normalization Analysis (VTNA) reported by Burés et al. (*Angew. Chem. Int. Ed.* **2016**, *55*, 16084–16087). Kinetic orders were determined via inspection of product formation curves when modifying the power (α) of the concentration-adjusted x-axis ($\Sigma[A]^{\alpha}\Delta t$) to account for the influence of a given reaction component (A) on the overall rate; the kinetic order that provides the best overlap of the product formation curves indicates the reaction order.

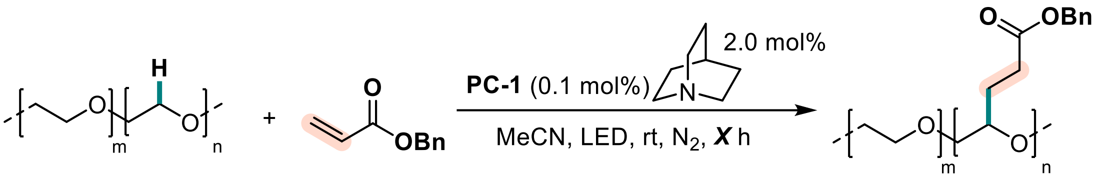

PEG 2000 2.0 mmol

1

20.0 mol%

PEG 2000-1

To a 4 mL vial were added **PC-1**, quinuclidine, PEG 2000, **1**, and MeCN (4.0 mL) in an N_2 glovebox. The vial was then sealed and transferred out of the glovebox. Under irradiation at 460 nm LEDs, the resulting mixture was stirred for 1, 2, 4, 6, 8, 10 or 12 hours at rt. Evaporation afforded crude **PEG 2000-1**. The level of functionalization was determined by ^{1}H NMR, CH_2Br_2 as internal standard.

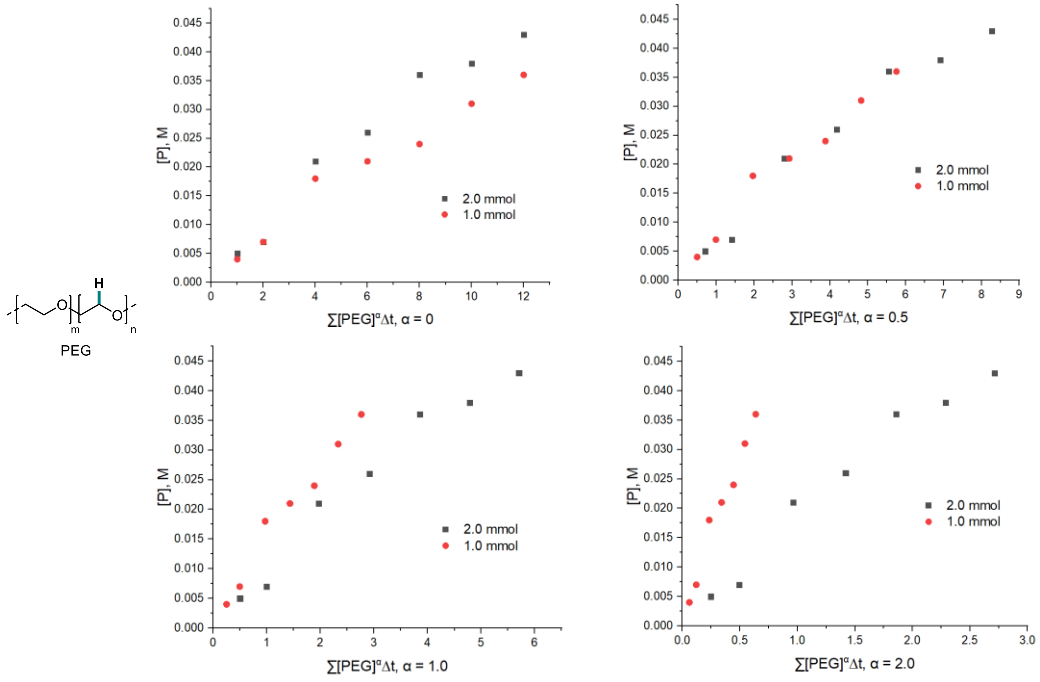

Supplementary Figure 4. The data suggests the reaction has ***0.5 order*** in PEG 2000

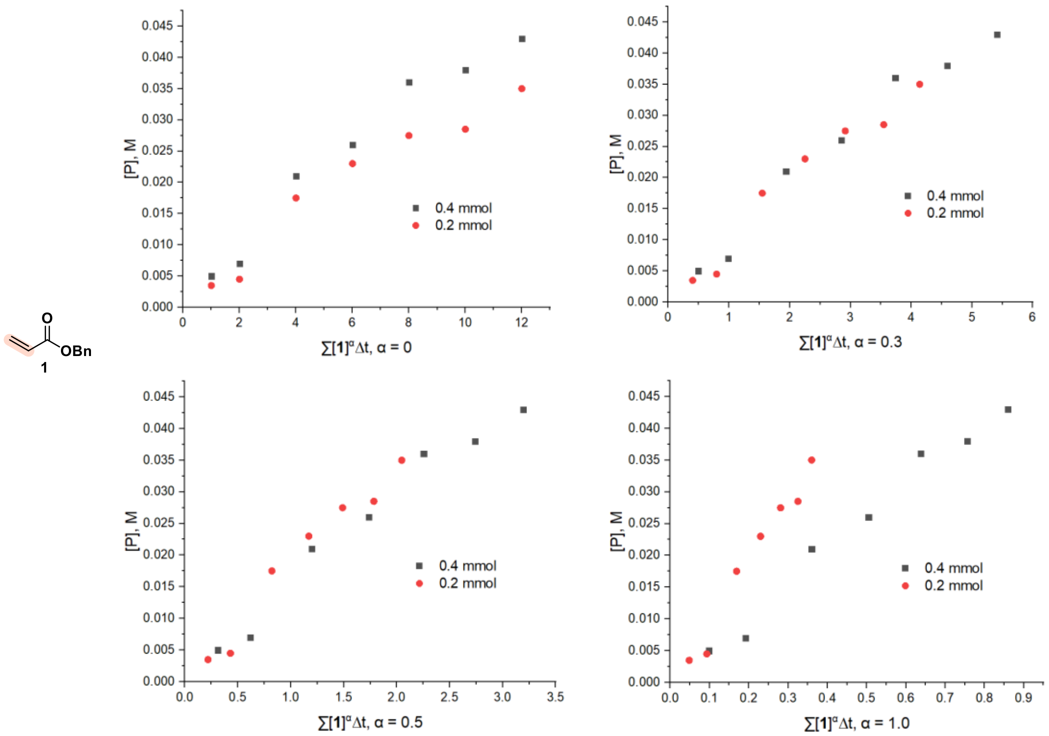

Supplementary Figure 5. The data suggests the reaction has ***0.3 order*** in acylate **1**

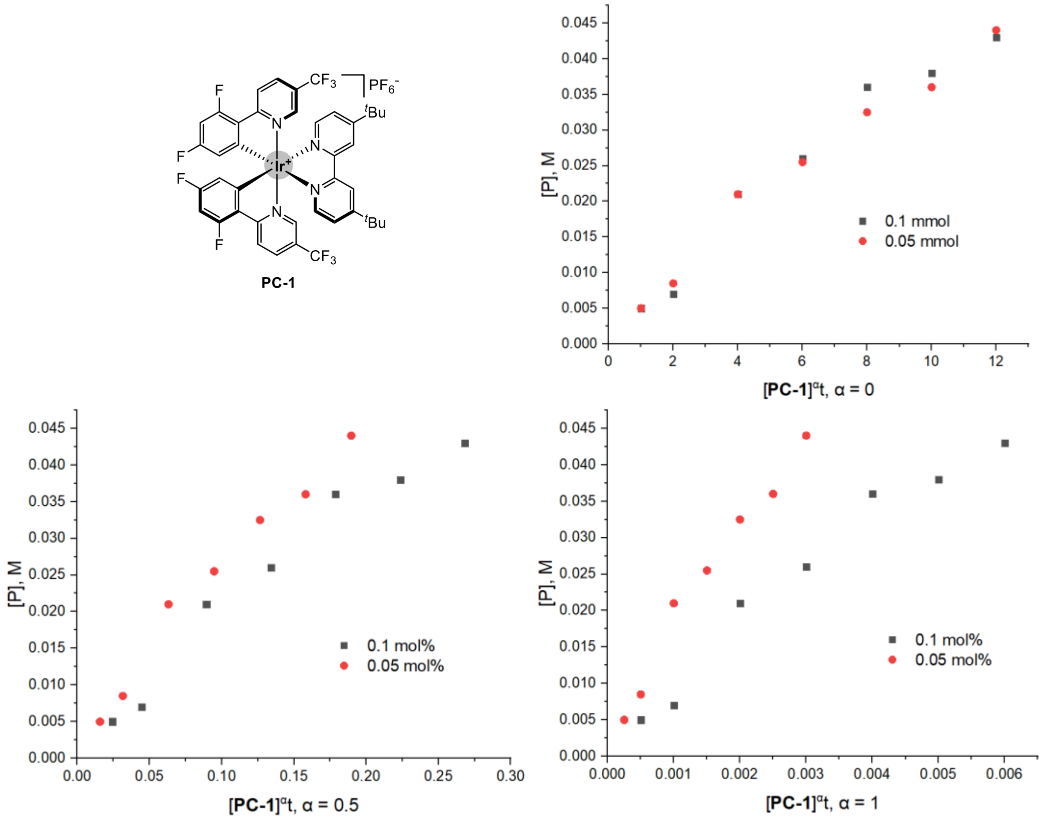

Supplementary Figure 6. The data suggests the reaction has ***0 order*** in **PC-1**

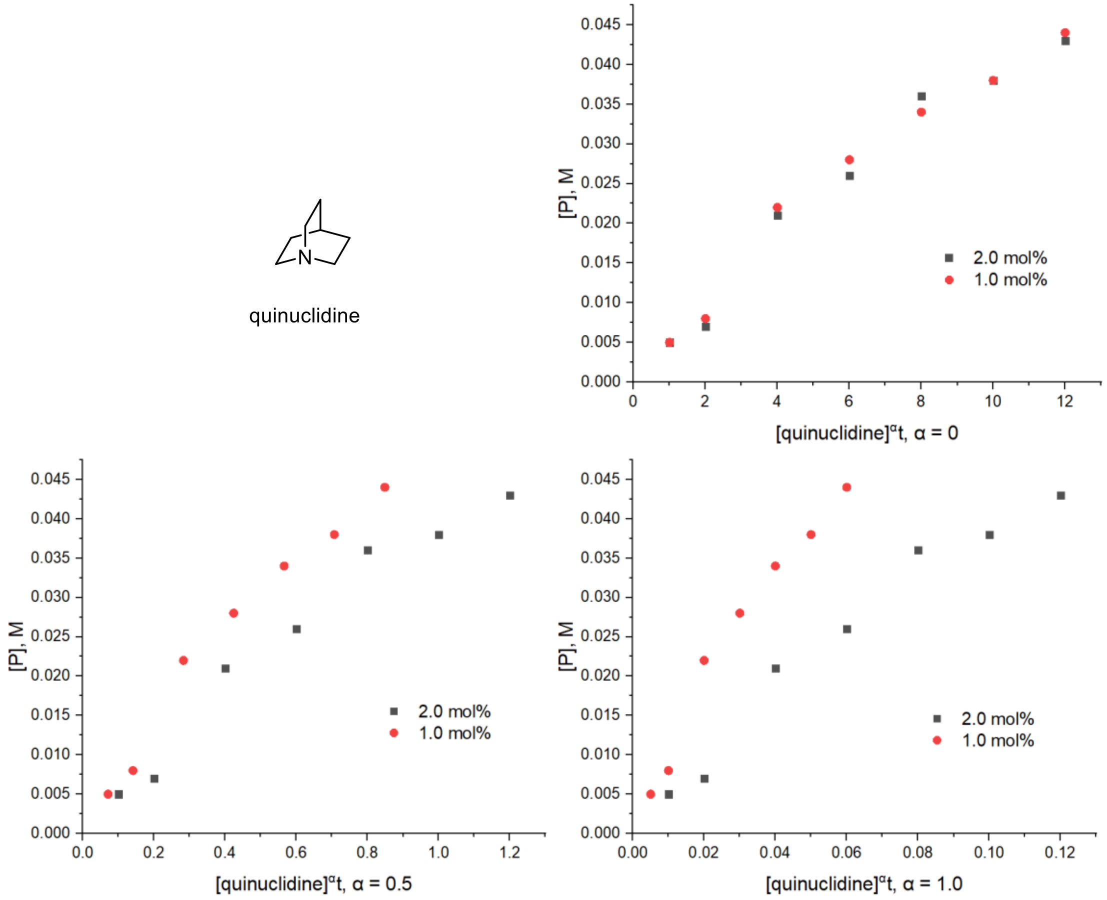

Supplementary Figure 7. The data suggests the reaction has ***0 order*** in quinuclidine

(4) The GPC traces and MALDI-TOF spectra using PEG 2000 with 2.

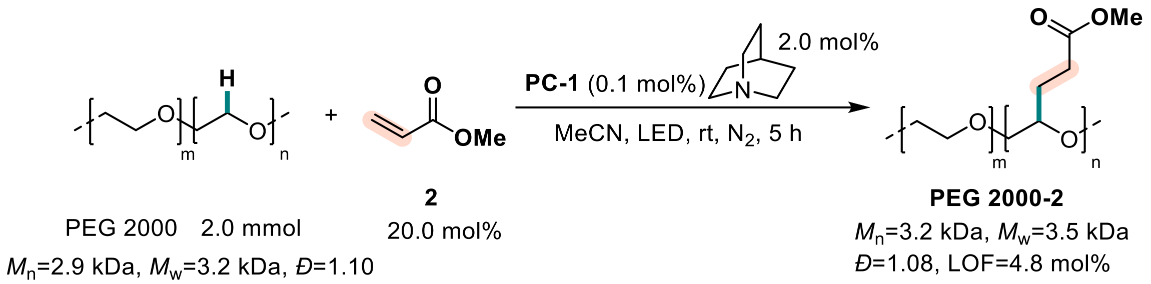

To a 4 mL vial were added **PC-1** (2.3 mg, 0.002 mmol), quinuclidine (4.6 mg, 0.04 mmol), PEG 2000 (88.5 mg, 2.0 mmol), **2** (36 µL, *d* = 0.956 g/mL, 34.4 mg, 0.4 mmol), and MeCN (4.0 mL) in an N_2 glovebox. The vial was then sealed and transferred out of the glovebox. Under irradiation at 460 nm LEDs, the resulting mixture was stirred for 5 hours at rt. Evaporation and Flash chromatography on silica gel (DCM to DCM/MeOH = 10/1) afforded **PEG 2000-2**: yellow oil (55.5 mg, LOF = 4.8 mol%). The level of functionalization was determined by ^{1}H NMR. The M_w, M_n, and $Đ$ values were determined by GPC.

^{1}H NMR (400 MHz, $CDCl_3$) δ 4.05-3.25 (m, **82.95 H**), 2.51-2.30 (m, 2.15 H), 2.00-1.69 (m, **2.00 H**), The ^{13}C NMR (151 MHz, $CDCl_3$) spectrum is very complicated, which is attached in the spectra part directly. **IR ν (neat, cm^{-1})** 3343, 2873, 1731, 1104.

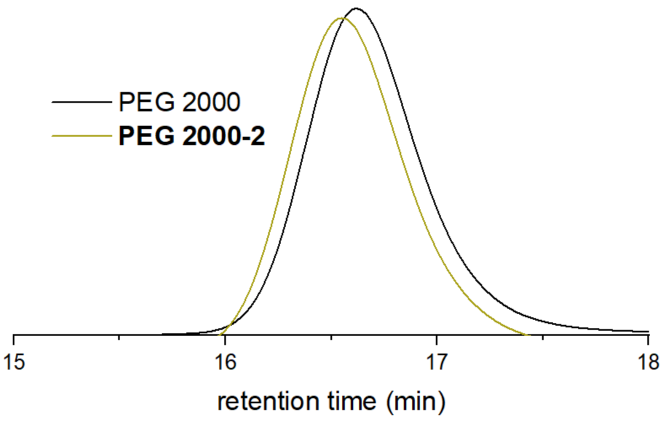

Entry	LOF (mol%)	M_w (g/mol)	M_n (g/mol)	$Đ$
1	0	3213	2913	1.10
2	4.8	3464	3195	1.08

Supplementary Figure 8. GPCs of **PEG 2000-2** (0.5 M) using 20 mol% of **2**.

We chose PEG 2000 and **PEG 2000-2** to analyze the details of molecule weight change before and after. When PEG_{45} was chosen as the example, we can find $[PEG_{45}+Na]^+$ = 2024.17 in starting material (black, below). After reacting with **2**, we did not find 2024.17, instead, some higher molecular weights could be obtained, assigning to the di-functionalized PEG_{45}.

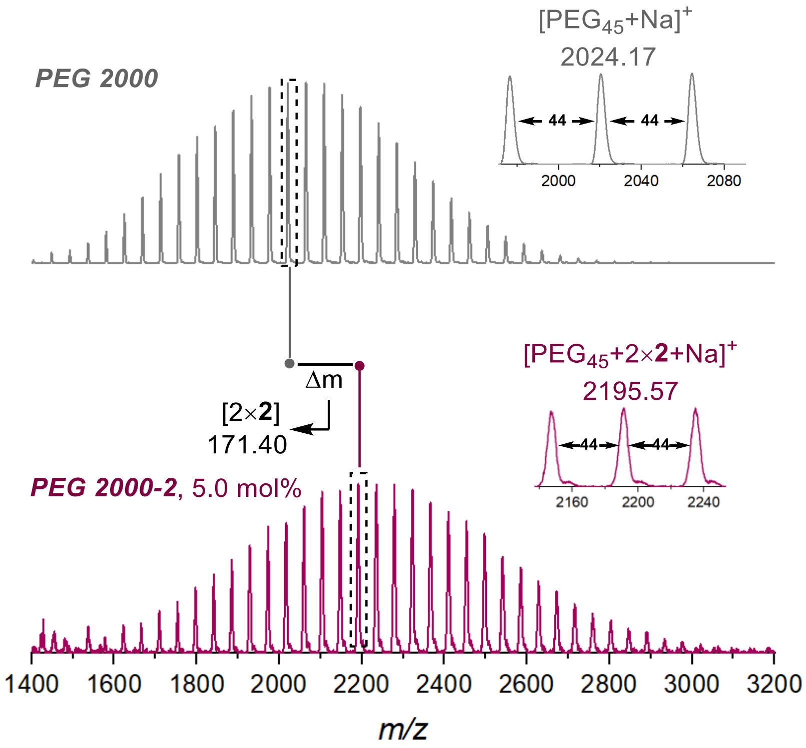

Supplementary Figure 9. Details of MALDI-TOF MS spectra for PEG 2000 and **PEG 2000-2**.

(5) Concern on the oligomerization

As for graftpolymerization and homopolymerization during reaction, we have conducted and examined the reaction of DME with acrylics **1** as a model reaction under the conditions. The reaction of DME, acrylics **1**, **PC-1**, quinuclidine and MeCN in an N_2 glovebox was conducted under irradiation at 460 nm LEDs for 24 hours at rt. The homopolymerization, the two molecules, as well as more than two molecules reaction were not observed by GC-MS.

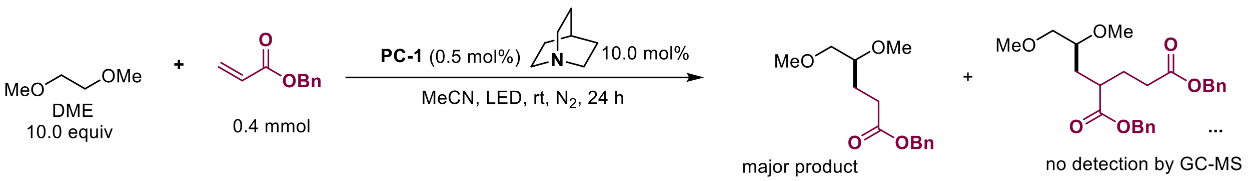

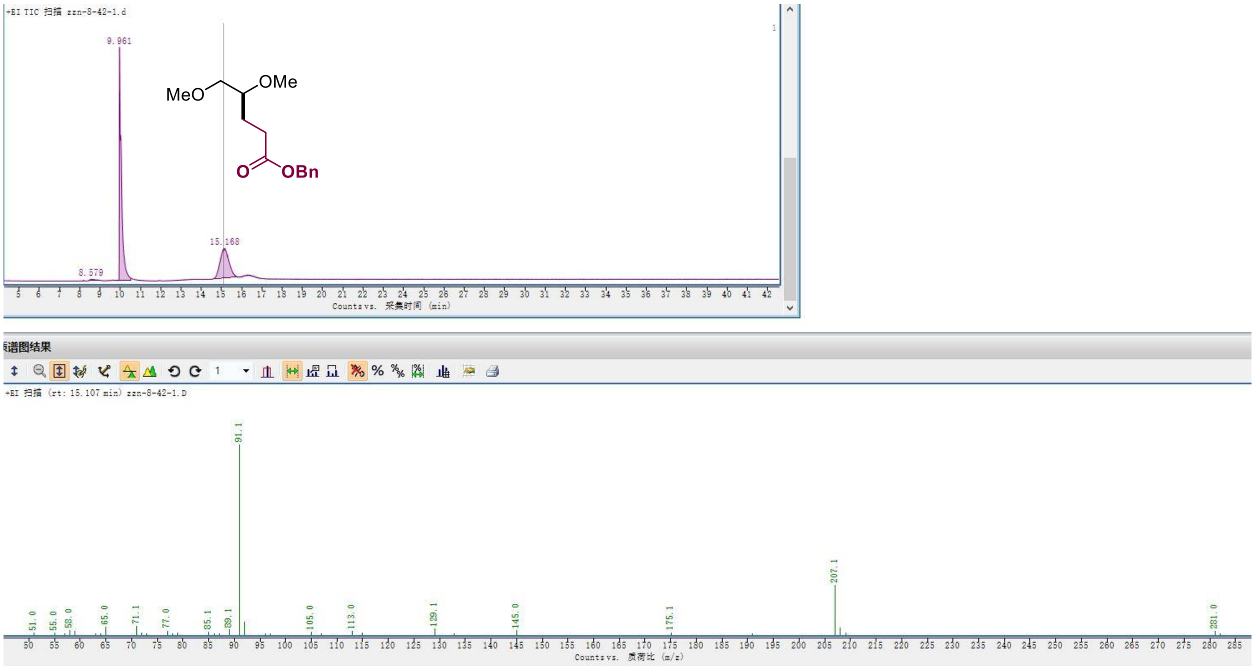

Supplementary Figure 10. Details of GC-MS spectra for **DME-1**.

(6) The C–H bond functionalization of the PEGs with various deficient alkenes

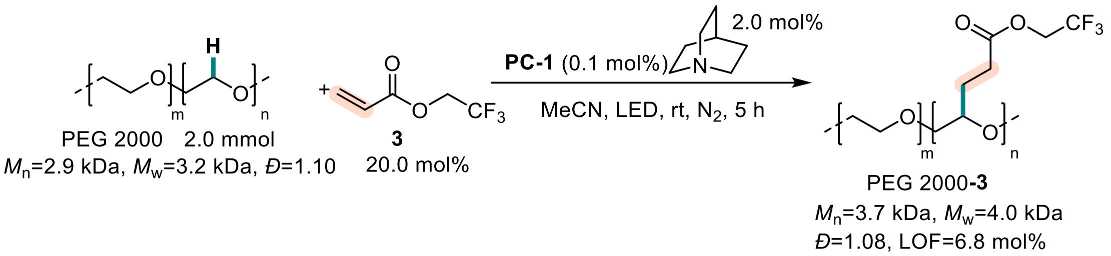

To a 4 mL vial were added **PC-1** (2.4 mg, 0.002 mmol), quinuclidine (4.5 mg, 0.04 mmol), PEG 2000 (88.5 mg, 2.0 mmol), **3** (52 μL, *d* = 1.216 g/mL, 61.6 mg, 0.4 mmol), and MeCN (4.0 mL) in an N_2 glovebox. The vial was then sealed and transferred out of the glovebox. Under irradiation at 460 nm LEDs, the resulting mixture was stirred for 5 hours at rt. Evaporation and Flash chromatography on silica gel (DCM to DCM/MeOH = 10/1) afforded **PEG 2000-3**: yellow oil (79.8 mg, LOF = 6.8 mol%). The level of functionalization was determined by ^{1}H NMR. The M_n and $Đ$ values were determined by GPC.

^{1}H NMR (400 MHz, $CDCl_3$) δ 4.50-4.26 (m, **2.00 H**), 3.80-3.28 (m, **59.24 H**), 2.67-2.45 (m, 2.02 H), 1.90-1.60 (m, 2.00 H). The ^{13}C NMR (151 MHz, $CDCl_3$) spectrum is very complicated, which is attached in the spectra part directly. **^{19}F NMR (376 MHz, $CDCl_3$)** δ 62.9, 72.2, 73.6, 74.1 **IR ν (neat, cm^{-1})** 3499, 2881, 1755, 1642, 1158.

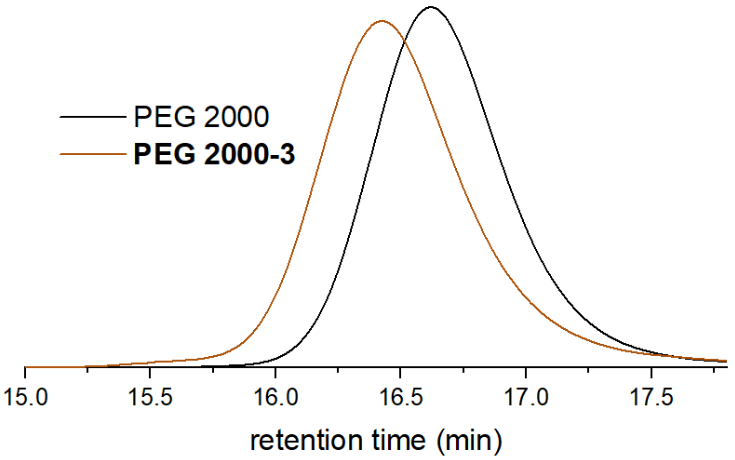

Entry	LOF (mol%)	M_w (g/mol)	M_n (g/mol)	$Đ$
1	0	3213	2913	1.10
2	6.8	3949	3659	1.08

Supplementary Figure 11. GPC of PEG 2000 and **PEG 2000-3**

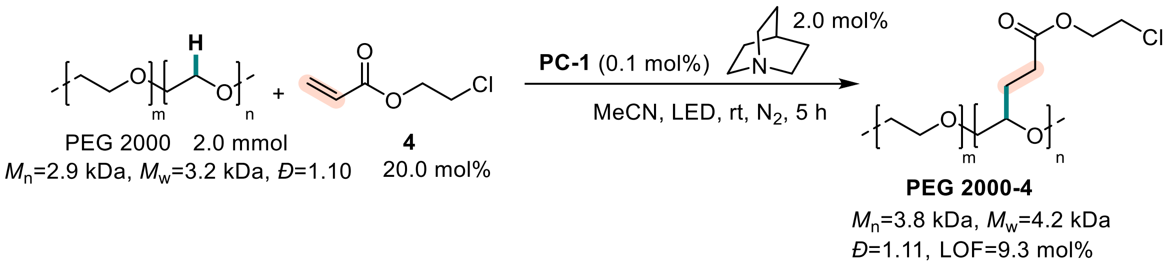

To a 4 mL vial were added **PC-1** (2.1 mg, 0.002 mmol), quinuclidine (4.2 mg, 0.04 mmol), PEG 2000 (88.7 mg, 2.0 mmol), **4** (48 μL, *d* = 1.125 g/mL, 53.60 mg, 0.4 mmol), and MeCN (4.0 mL) in an N_2 glovebox. The vial was then sealed and transferred out of the glovebox. Under irradiation at 460 nm LEDs, the resulting mixture was stirred for 5 hours at rt. Evaporation and Flash chromatography on silica gel (DCM to DCM/MeOH = 10/1) afforded PEG 2000-**4**: yellow oil (70.3 mg, LOF = 9.3 mol%). The level of functionalization was determined by ^{1}H NMR.

^{1}H NMR (400 MHz, $CDCl_3$) δ 4.45-4.23 (m, **1.00 H**), 3.82-3.35 (m, **21.45 H**), 2.57-2.38 (m, 0.90 H), 1.92-1.69 (m, 0.75 H). The ^{13}C NMR (151 MHz, $CDCl_3$) spectrum is very complicated, which is attached in the spectra part directly. **IR ν (neat, cm^{-1})** 2876, 1736, 1102.

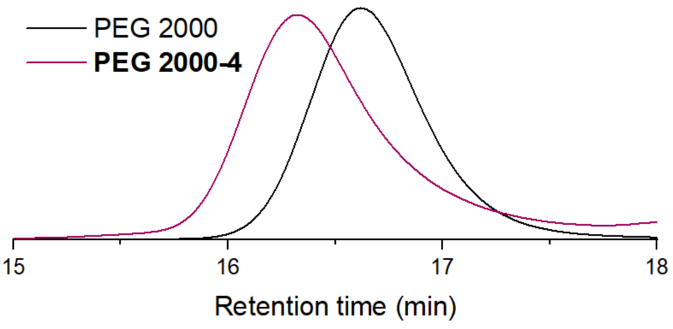

Entry	LOF (mol%)	M_w (g/mol)	M_n (g/mol)	*Đ*
1	0	3213	2913	1.10
2	9.3	4187	3760	1.11

Supplementary Figure 12. GPC of PEG 2000 and **PEG 2000-4**

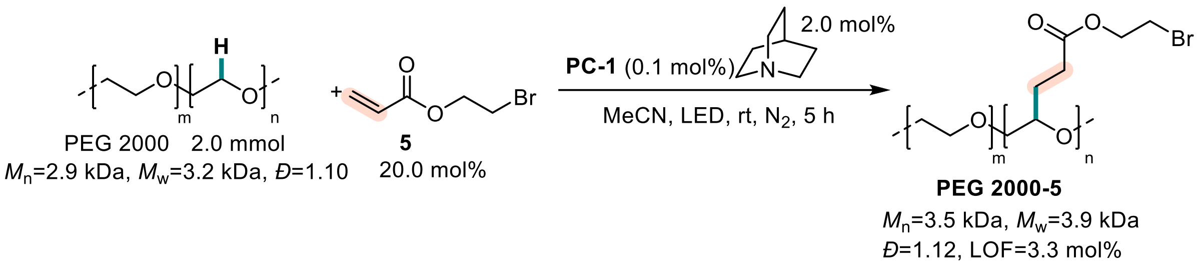

To a 4 mL vial were added **PC-1** (2.3 mg, 0.002 mmol), quinuclidine (4.5 mg, 0.04 mmol), PEG 2000 (88.2 mg, 2.0 mmol), **4** (49 μL, *d* = 1.458 g/mL, 71.6 mg, 0.4 mmol), and MeCN (4.0 mL) in an N_2 glovebox. The vial was then sealed and transferred out of the glovebox. Under irradiation at 460 nm LEDs, the resulting mixture was stirred for 5 hours at rt. Evaporation and Flash chromatography on silica gel (DCM to DCM/MeOH = 10/1) afforded **PEG 2000-5**: yellow oil (52.4 mg, LOF = 3.3 mol%). The level of functionalization was determined by ^{1}H NMR.

^{1}H NMR (400 MHz, $CDCl_3$) δ 4.50-4.25 (m, **1.00 H**), 3.82-3.35 (m, **61.23 H**). The ^{13}C NMR (151 MHz, $CDCl_3$) spectrum is very complicated, which is attached in the spectra part directly. **IR ν (neat, cm^{-1})** 3484, 2876, 1735, 1103.

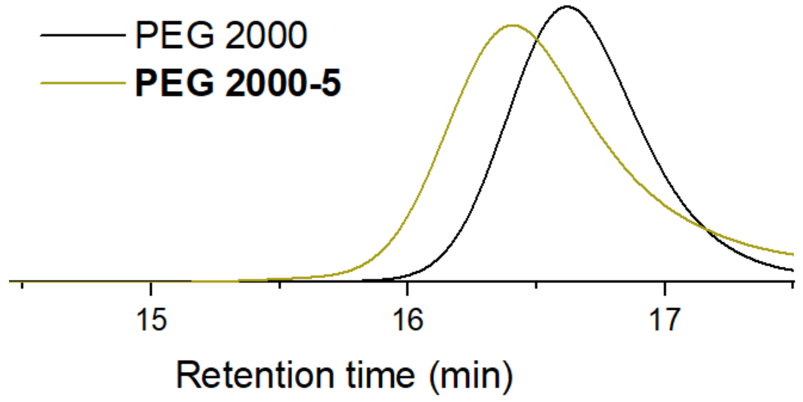

Entry	LOF (mol%)	M_w (g/mol)	M_n (g/mol)	*Đ*
1	0	3213	2913	1.10
2	3.3	3879	3454	1.12

Supplementary Figure 13. GPC of PEG 2000 and **PEG 2000-5**

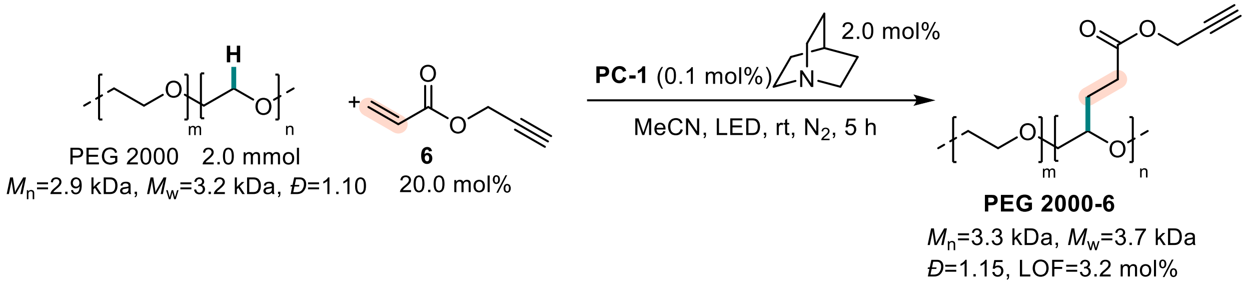

To a 4 mL vial were added **PC-1** (2.3 mg, 0.002 mmol), quinuclidine (4.6 mg, 0.04 mmol), PEG 2000 (88.6 mg, 2.0 mmol), **6** (45 μL, d = 0.997 g/mL, 44.0 mg, 0.4 mmol), and MeCN (4.0 mL) in an N_2 glovebox. The vial was then sealed and transferred out of the glovebox. Under irradiation at 460 nm LEDs, the resulting mixture was stirred for 5 hours at rt. Evaporation and Flash chromatography on silica gel (DCM to DCM/MeOH = 10/1) afforded **PEG 2000-6**: yellow oil (79.8 mg, LOF = 3.2 mol%). The level of functionalization was determined by ^{1}H NMR. The M_n and $Đ$ values were determined by GPC.

^{1}H NMR (400 MHz, $CDCl_3$) δ 4.70-4.62 (m, **2.00 H**), 3.82-3.35 (m, **24.02 H**), 2.56-2.45 (m, 2.31 H), 1.93-1.70 (m, 1.59 H). The ^{13}C NMR (151 MHz, $CDCl_3$) spectrum is very complicated, which is attached in the spectra part directly. **IR ν (neat, cm^{-1})** 2906, 2357, 1745, 1115.

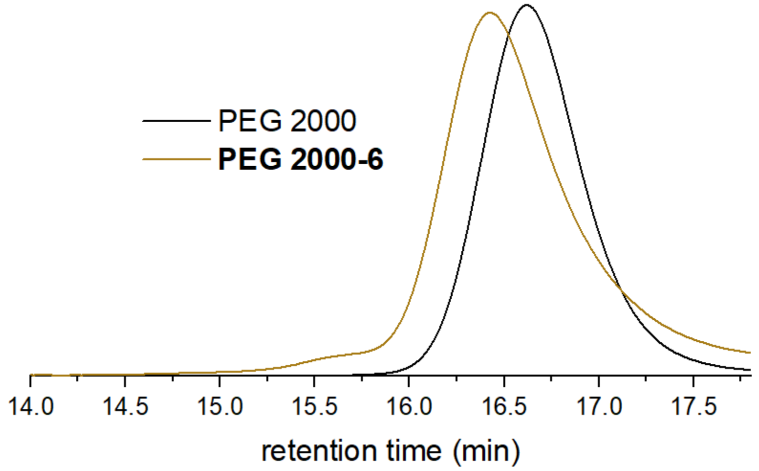

Entry	LOF (mol%)	M_w (g/mol)	M_n (g/mol)	$Đ$
1	0	3213	2913	1.10
2	3.2	3739	3254	1.15

Supplementary Figure 14. GPC of PEG 2000 and **PEG 2000-6**

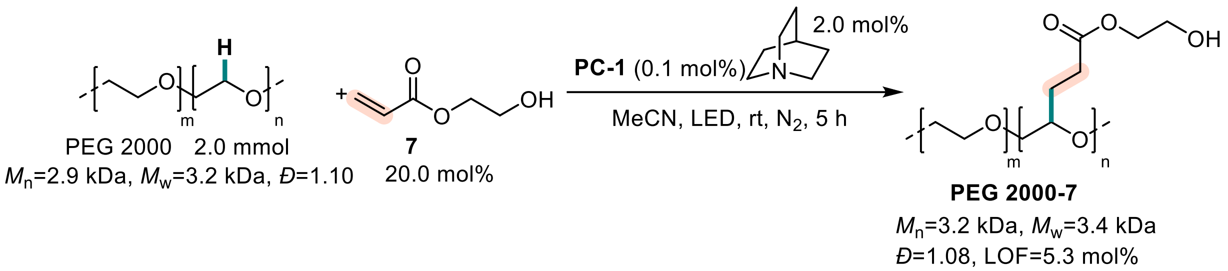

To a 4 mL vial were added **PC-1** (2.3 mg, 0.002 mmol), quinuclidine (4.3 mg, 0.04 mmol), PEG 2000 (88.6 mg, 2.0 mmol), **7** (42 μL, *d* = 1.106 g/mL, 46.4 mg, 0.4 mmol), and MeCN (4.0 mL) in an N_2 glovebox. The vial was then sealed and transferred out of the glovebox. Under irradiation at 460 nm LEDs, the resulting mixture was stirred for 5 hours at rt. Evaporation and Flash chromatography on silica gel (DCM to DCM/MeOH = 10/1) afforded **PEG 2000-7**: yellow oil (91.6 mg, LOF = 5.3 mol%). The level of functionalization was determined by ^{1}H NMR. The M_n and *Đ* values were determined by GPC.

^{1}H NMR (400 MHz, $CDCl_3$) δ 4.45-4.10 (m, **2.00 H**), 3.75-3.39 (m, **77.15 H**), 2.4-2.25 (m, **2.00 H**), 2.00-1.42 (m, **1.65 H**). The ^{13}C NMR (151 MHz, $CDCl_3$) spectrum is very complicated, which is attached in the spectra part directly. **IR ν (neat, cm^{-1})** 3422, 2920, 1733, 1648, 1108.

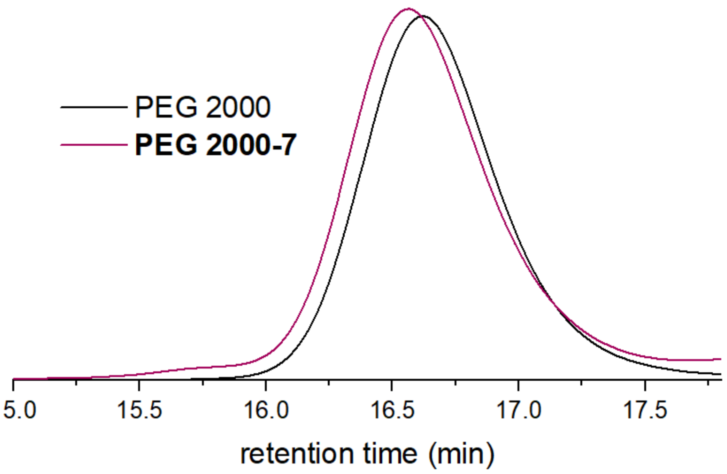

Entry	LOF (mol%)	M_w (g/mol)	M_n (g/mol)	*Đ*
1	0	3213	2913	1.10
2	5.3	3419	3168	1.08

Supplementary Figure 15. GPC of PEG 2000 and **PEG 2000-7**

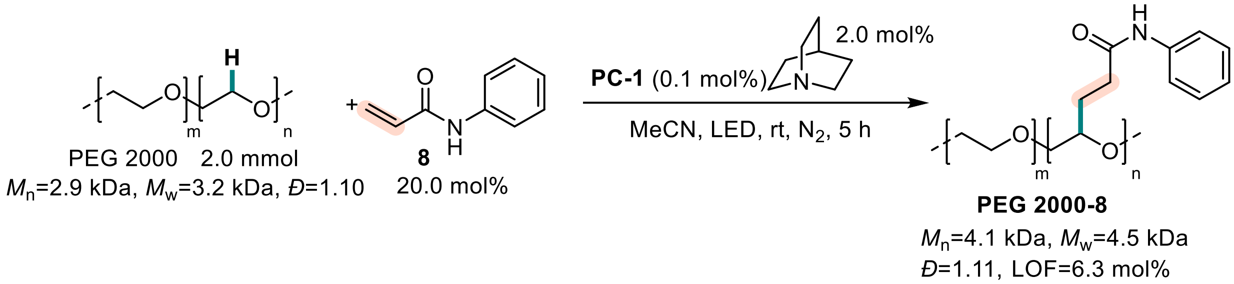

To a 4 mL vial were added **PC-1** (2.5 mg, 0.002 mmol), quinuclidine (4.6 mg, 0.04 mmol), PEG 2000 (88.7 mg, 2.0 mmol), **8** (58.2 mg, 0.4 mmol), and MeCN (4.0 mL) in an N_2 glovebox. The vial was then sealed and transferred out of the glovebox. Under irradiation at 460 nm LEDs, the resulting mixture was stirred for 5 hours at rt. Evaporation and the resulting mixture was precipitated 3 times using DCM and Et_2O (DCM/Et_2O = 1:8) until the small molecules were removed completely. The compound **PEG 2000-8** was obtained as a yellow solid (82.4 mg, LOF = 6.3 mol%). The level of functionalization was determined by ^{1}H NMR. The M_n and Đ values were determined by GPC.

^{1}H NMR (400 MHz, $CDCl_3$) δ 8.75-7.52 (m, **2.00 H**), 7.28-7.18 (m, 2.75 H), 7.06-6.92(m, 1.10 **H**), 4.03-3.22 (m, **63.23 H**). The ^{13}C NMR (151 MHz, $CDCl_3$) spectrum is very complicated, which is attached in the spectra part directly. **IR ν (neat, cm^{-1})** 3451, 2871, 1680, 1103.

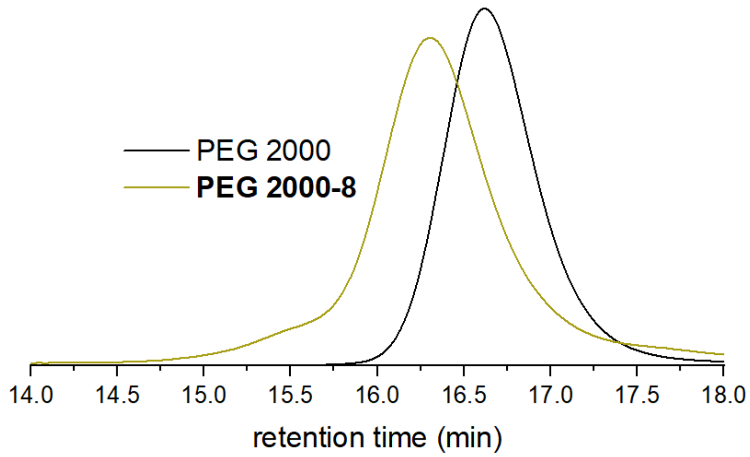

Entry	LOF (mol%)	M_w (g/mol)	M_n (g/mol)	Đ
1	0	3213	2913	1.10
2	6.3	4535	4071	1.11

Supplementary Figure 16. GPC of PEG 2000 and **PEG 2000-8**

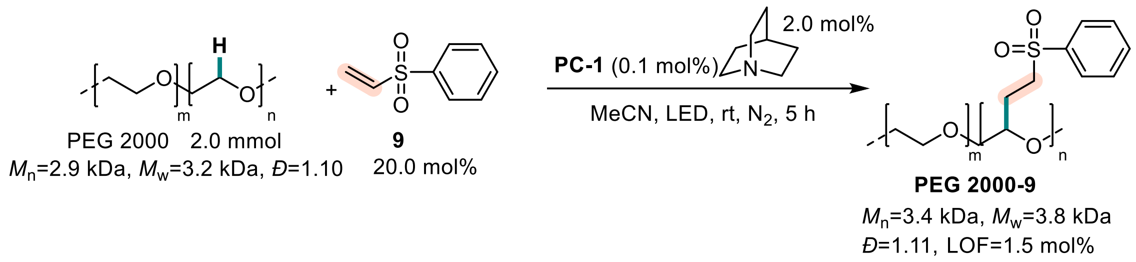

To a 4 mL vial were added **PC-1** (2.3 mg, 0.002 mmol), quinuclidine (4.5 mg, 0.04 mmol), PEG 2000 (88.6 mg, 2.0 mmol), **9** (67.8 mg, 0.4 mmol), and MeCN (4.0 mL) in an N_2 glovebox. The vial was then sealed and transferred out of the glovebox. Under irradiation at 460 nm LEDs, the resulting mixture was stirred for 5 hours at rt. Evaporation and the resulting mixture was precipitated 3 times using DCM and Et_2O (DCM/Et_2O = 1:8) until the small molecules were removed completely. The compound **PEG-2000-9** was obtained as a yellow solid (81.7 mg, LOF = 1.5 mol%). The level of functionalization was determined by ^{1}H NMR. The M_n and $Đ$ values were determined by GPC.

^{1}H NMR (400 MHz, $CDCl_3$) δ 7.93-7.78 (m, 1.58 H), 7.60-7.50 (m, 3.28 H), 3.95-3.45(m, **259.83 H**), 2.85-2.76 (m, **2.00 H**). The ^{13}C NMR (151 MHz, $CDCl_3$) spectrum is very complicated, which is attached in the spectra part directly. **IR ν (neat, cm^{-1})** 3421, 2872, 1636, 1457, 1104.

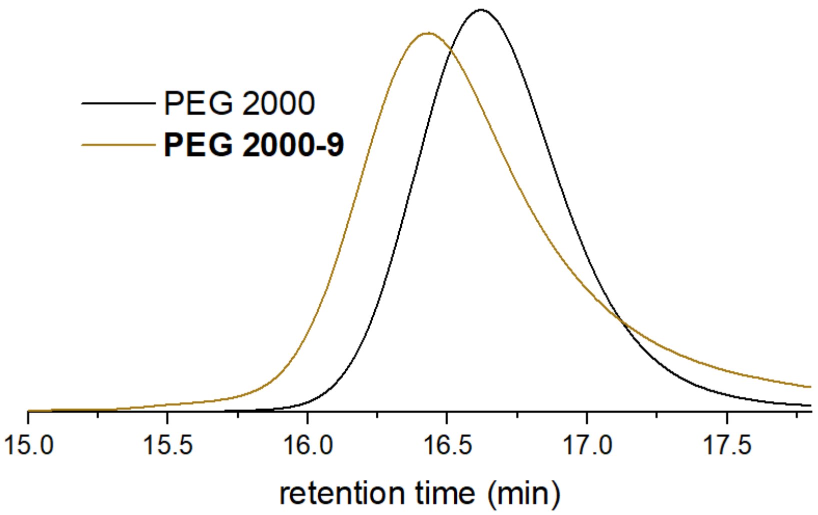

Entry	LOF (mol%)	M_w (g/mol)	M_n (g/mol)	$Đ$
1	0	3213	2913	1.10
2	1.5	3786	3404	1.11

Supplementary Figure 17. GPC of PEG 2000 and **PEG 2000-9**

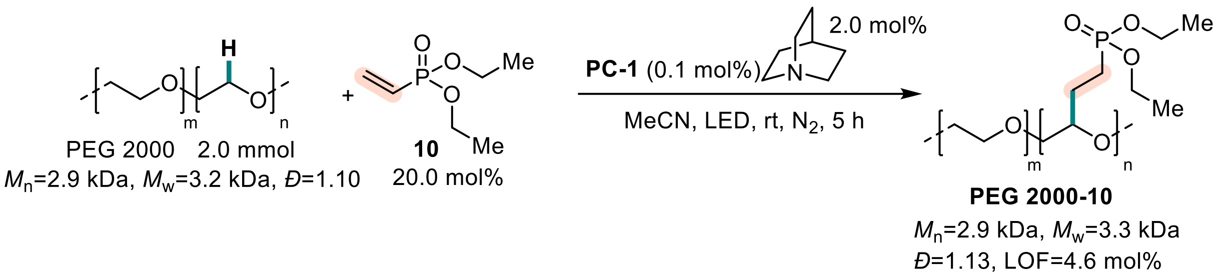

To a 4 mL vial were added **PC-1** (2.1 mg, 0.002 mmol), quinuclidine (4.2 mg, 0.04 mmol), PEG 2000 (88.2 mg, 2.0 mmol), **10** (62 μL, *d* = 1.068 g/mL, 65.6 mg 0.4 mmol), and MeCN (4.0 mL) in an N_2 glovebox. The vial was then sealed and transferred out of the glovebox. Under irradiation at 460 nm LEDs, the resulting mixture was stirred for 5 hours at rt. Evaporation and Flash chromatography on silica gel (DCM to DCM/MeOH = 10/1) afforded **PEG 2000-10**: yellow oil (79.0 mg, LOF = 4.6 mol%). The level of functionalization was determined by ^{1}H NMR. The M_n and *Đ* values were determined by GPC.

^{1}H NMR (400 MHz, $CDCl_3$) δ 4.20-4.12 (m, **4.00 H**), 3.72-3.35 (m, **86.56 H**), 1.40-1.20 (m, **6.23 H**).^{13}C NMR (151 MHz, $CDCl_3$) spectrum is very complicated, which is attached in the spectra part directly. **^{31}P NMR (162 MHz, $CDCl_3$)** δ 33.3. **IR ν (neat, cm^{-1})** 3435, 2872, 1643, 1351, 1110.

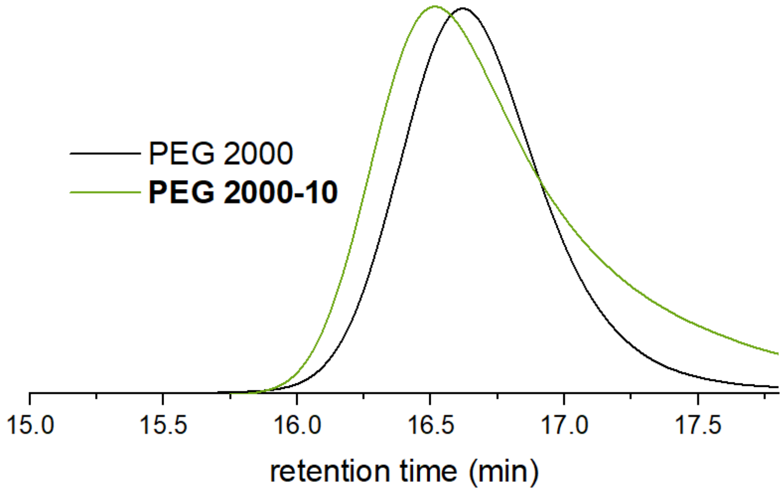

Entry	LOF (mol%)	M_w (g/mol)	M_n (g/mol)	*Đ*
1	0	2808	2913	1.10
2	4.6	3134	3404	1.12

Supplementary Figure 18. GPC of PEG 2000 and **PEG 2000-10**

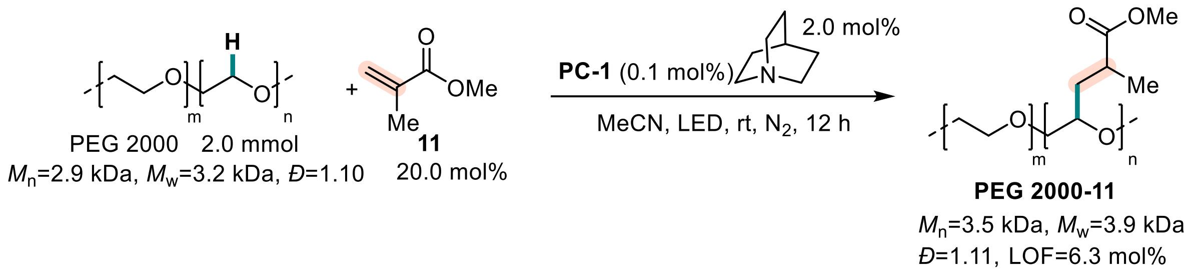

To a 4 mL vial were added **PC-1** (2.3 mg, 0.002 mmol), quinuclidine (4.4 mg, 0.04 mmol), PEG 2000 (88.5 mg, 2.0 mmol), **11** (43 μL, *d* = 0.936 g/mL, 40.0 mg, 0.4 mmol), and MeCN (4.0 mL) in an N_2 glovebox. The vial was then sealed and transferred out of the glovebox. Under irradiation at 460 nm LEDs, the resulting mixture was stirred for 12 hours at rt. Evaporation and Flash chromatography on silica gel (DCM to DCM/MeOH = 10/1) afforded **PEG 2000-11**: yellow oil (69.0 mg, LOF = 6.3 mol%). The level of functionalization was determined by ^{1}H NMR. The M_n and *Đ* values were determined by GPC.

^{1}H NMR (400 MHz, $CDCl_3$) δ 4.00-3.47 (m, **63.72 H**), 2.75-2.55 (m, 0.92 H), 1.98-1.75 (m, **1.00 H**), 1.60-1.45 (m, 1.04 H), 1.22-1.15 (m, 3.07 H). ^{13}C NMR (151 MHz, $CDCl_3$) spectrum is very complicated, which is attached in the spectra part directly. **IR ν (neat, cm^{-1})** 2873, 1731, 1458, 1111.

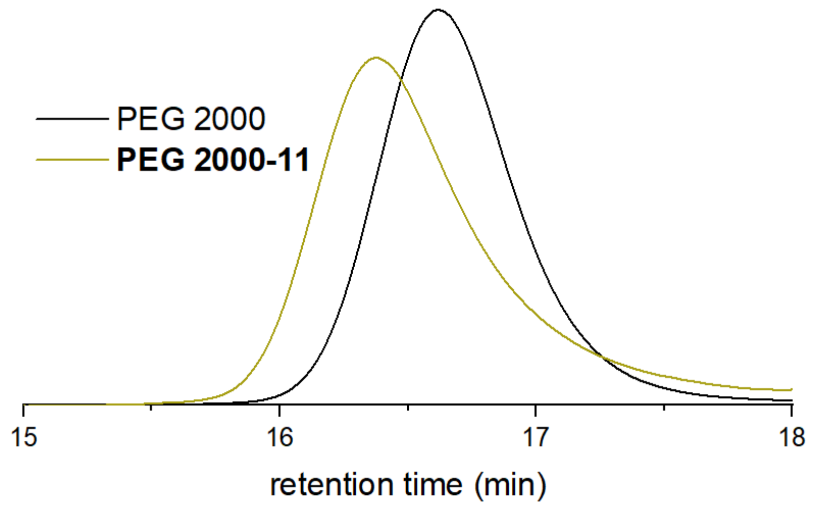

Entry	LOF (mol%)	M_w (g/mol)	M_n (g/mol)	*Đ*
1	0	2808	2913	1.10
2	6.3	3904	3528	1.11

Supplementary Figure 19. GPC of PEG 2000 and **PEG 2000-11**

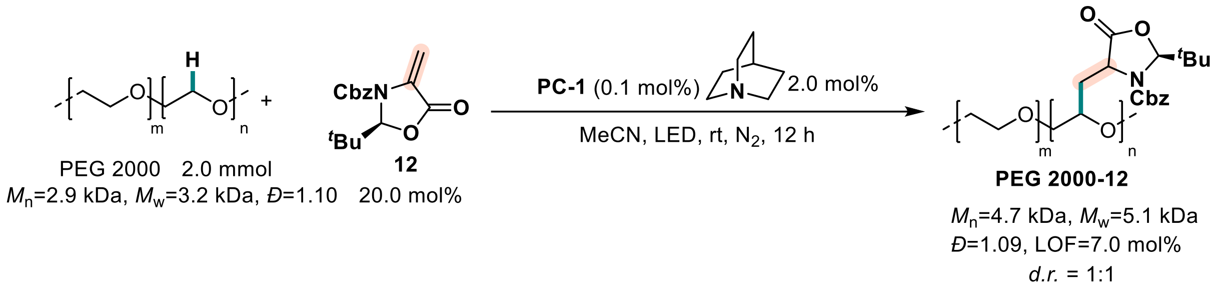

To a 4 mL vial were added **PC-1** (2.3 mg, 0.002 mmol), quinuclidine (4.6 mg, 0.04 mmol), PEG 2000 (88.2 mg, 2.0 mmol), **12** (115.2mg, 0.4 mmol), and MeCN (4.0 mL) in an N_2 glovebox. The vial was then sealed and transferred out of the glovebox. Under irradiation at 460 nm LEDs, the resulting mixture was stirred for 12 hours at rt. Evaporation and Flash chromatography on silica gel (DCM to DCM/MeOH = 10/1) afforded **PEG 2000-12**: yellow oil (89.3 mg, LOF = 7.0 mol%, *d.r.* = 1:1). The level of functionalization was determined by ^{1}H NMR. The M_n and *Đ* values were determined by GPC.

^{1}H NMR (400 MHz, $CDCl_3$) δ 7.50-7.27 (m, 5.71 H), 5.65-5.50 (m, **1.00** H), 5.25-5.05 (m, 2.16 H), 4.75-4.50 (m, 0.92 H), 3.85-3.28 (m, **57.25 H**), 2.24-1.82 (m, 2.13 H), 1.12-0.85 (m, 10.41 H).^{13}C NMR (151 MHz, $CDCl_3$) spectrum is very complicated, which is attached in the spectra part directly. **IR ν (neat, cm^{-1})** 2873, 1789, 1716, 1115.

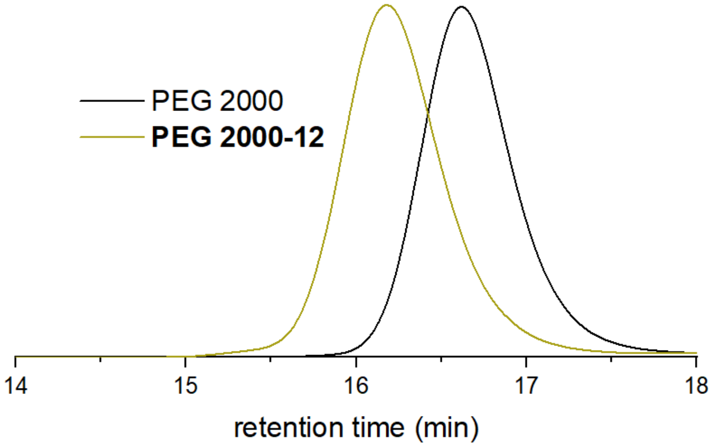

Entry	LOF (mol%)	M_w (g/mol)	M_n (g/mol)	*Đ*
1	0	2808	2913	1.10
2	7.0	5086	4663	1.09

Supplementary Figure 20. GPC of PEG 2000 and **PEG 2000-12**

(7) PEGylation of pharmaceuticals with PEG 12000

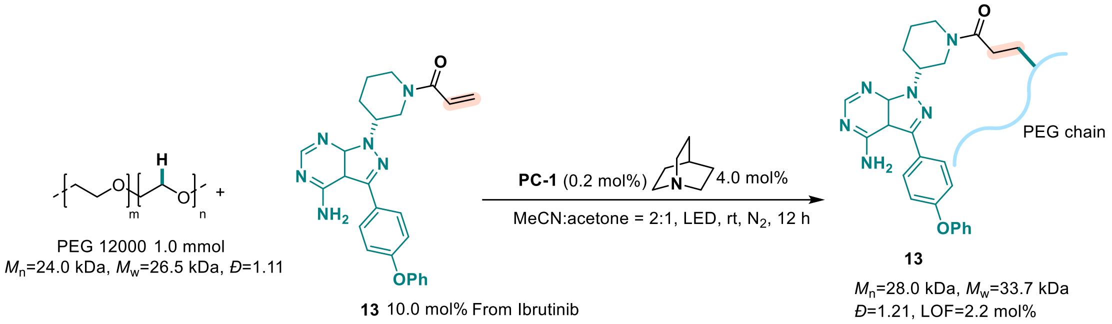

To a 4 mL vial were added **PC-1** (2.3 mg, 0.002 mmol), quinuclidine (4.6 mg, 0.04 mmol), PEG 12000 (44.3 mg, 1.0 mmol), **13** (44.5 mg, 0.1 mmol), and MeCN:acetone = 2:1 (3.0 mL) in an N_2 glovebox. The vial was then sealed and transferred out of the glovebox. Under irradiation at 460 nm LEDs, the resulting mixture was stirred for 12 hours at rt. Evaporation and the resulting mixture was precipitated 3 times using DCM and Et_2O (DCM/Et_2O = 1:8) until the small molecules were removed completely. The compound **PEG 12000-13** was obtained as a yellow solid (54.4 mg, LOF = 2.2 mol%). The level of functionalization was determined by ^{1}H NMR. The M_n and *Đ* values were determined by GPC.

^{1}H NMR (400 MHz, $CDCl_3$) δ 7.75-7.50 (m, **2.00 H**), 3.85-3.28 (m, **184.43 H**).^{13}C NMR (151 MHz, $CDCl_3$) spectrum is very complicated, which is attached in the spectra part directly. **IR ν (neat, cm^{-1})** 2872, 1634, 1443, 1108.

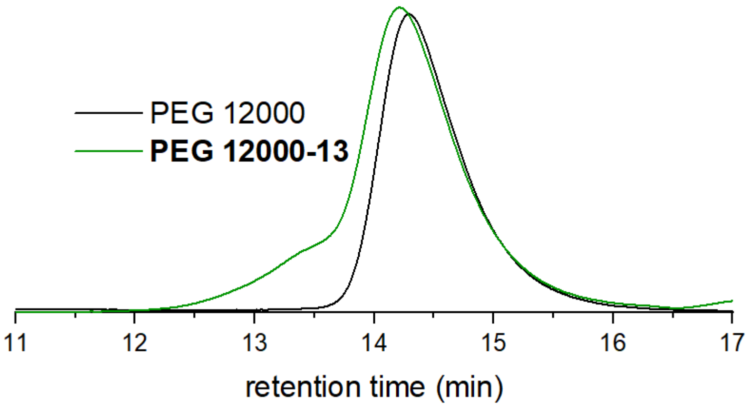

Entry	LOF (mol%)	M_w (g/mol)	M_n (g/mol)	*Đ*
1	0	26529	23981	1.10
2	2.2	33726	27964	1.21

Supplementary Figure 21. GPC of PEG 12000 and **PEG 12000-13**

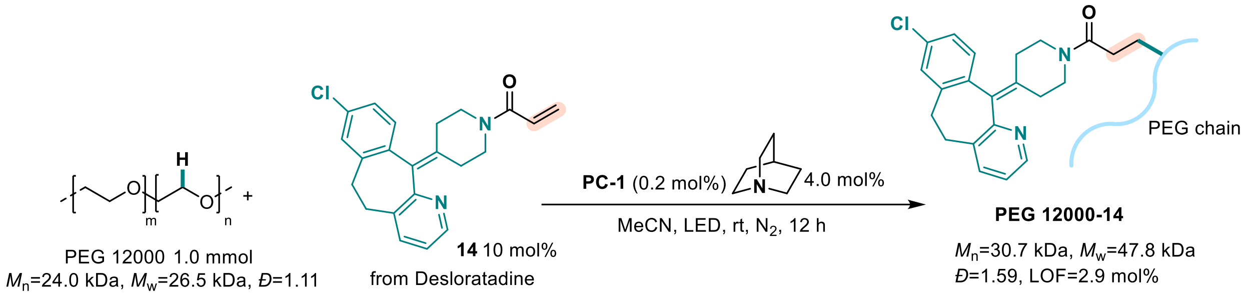

To a 4 mL vial were added **PC-1** (2.4 mg, 0.002 mmol), quinuclidine (4.2 mg, 0.04 mmol), PEG 12000 (44.2 mg, 1.0 mmol), **14** (36.8 mg, 0.1 mmol), and MeCN (2.0 mL) in an N_2 glovebox. The vial was then sealed and transferred out of the glovebox. Under irradiation at 460 nm LEDs, the resulting mixture was stirred for 12 hours at rt. Evaporation and the resulting mixture was precipitated 3 times using DCM and Et_2O (DCM/Et_2O = 1:8) until the small molecules were removed completely. The compound **PEG 12000-13** was obtained as a red solid (85.2 mg, LOF = 2.9 mol%). The level of functionalization was determined by ^{1}H NMR. The M_n and $Đ$ values were determined by GPC.

^{1}H NMR (400 MHz, $CDCl_3$) δ 8.50-8.25 (m, **1.00 H**), 7.50-7.27 (m, 1.23 H), 7.25-7.00 (m, 5.15 H), 3.85-3.28 (m, **138.78 H**).^{13}C NMR (151 MHz, $CDCl_3$) spectrum is very complicated, which is attached in the spectra part directly. **IR ν (neat, cm^{-1})** 3481, 2871, 1633, 1103.

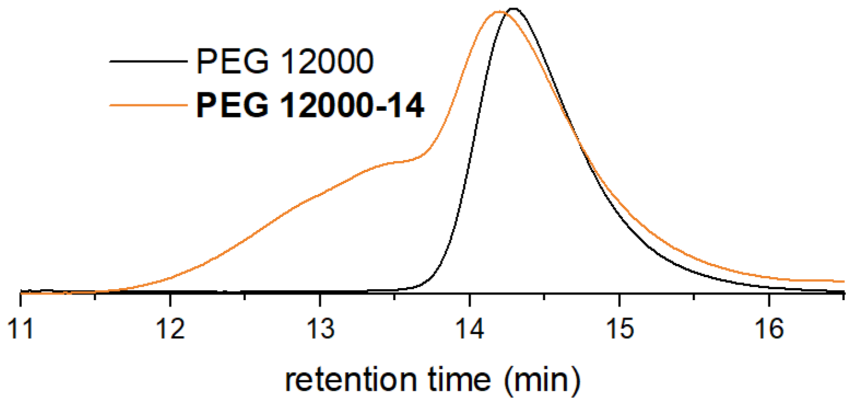

Entry	LOF (mol%)	M_w (g/mol)	M_n (g/mol)	$Đ$
1	0	26529	23981	1.10
2	2.9	48788	30690	1.59

Supplementary Figure 22. GPC of PEG 12000 and **PEG 12000-14**

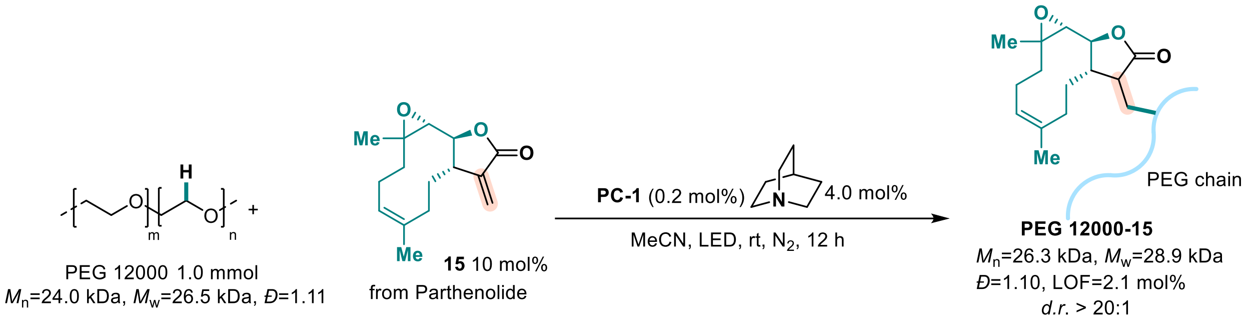

To a 4 mL vial were added **PC-1** (2.2 mg, 0.002 mmol), quinuclidine (4.6 mg, 0.04 mmol), PEG 12000 (44.2 mg, 1.0 mmol), **15** (24.9 mg, 0.1 mmol), and MeCN (2.0 mL) in an N_2 glovebox. The vial was then sealed and transferred out of the glovebox. Under irradiation at 460 nm LEDs, the resulting mixture was stirred for 12 hours at rt. Evaporation and the resulting mixture was precipitated 3 times using DCM and Et_2O (DCM/Et_2O = 1:8) until the small molecules were removed completely. The compound **PEG 12000-15** was obtained as a red solid (44.0 mg, LOF = 2.9 mol%, *d.r.* > 20:1). The level of functionalization was determined by ^{1}H NMR. The M_n and $Đ$ values were determined by GPC.

^{1}H NMR (400 MHz, $CDCl_3$) δ 5.25-5.50 (m, **1.00 H**), 3.85-3.28 (m, **182.00 H**). ^{13}C NMR (151 MHz, $CDCl_3$) spectrum is very complicated, which is attached in the spectra part directly. **IR ν (neat, cm^{-1})** 2870, 1761, 1349, 1102.

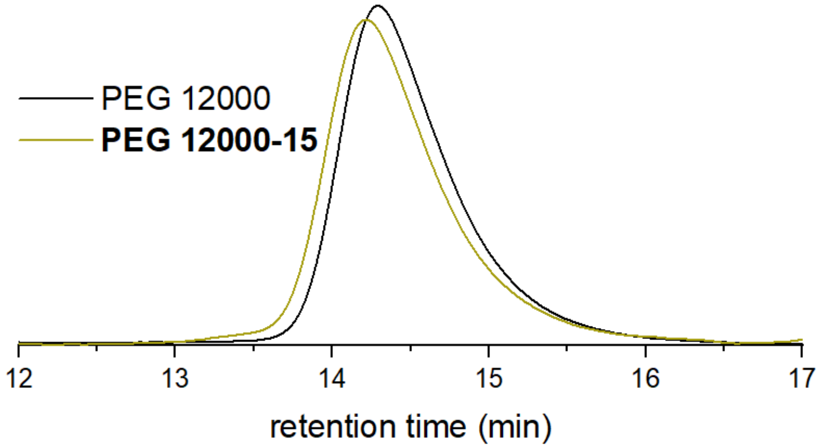

Entry	LOF (mol%)	M_w (g/mol)	M_n (g/mol)	$Đ$
1	0	26529	23981	1.10
2	2.1	28937	26335	1.10

Supplementary Figure 23. GPC of PEG 12000 and **PEG 12000-15**

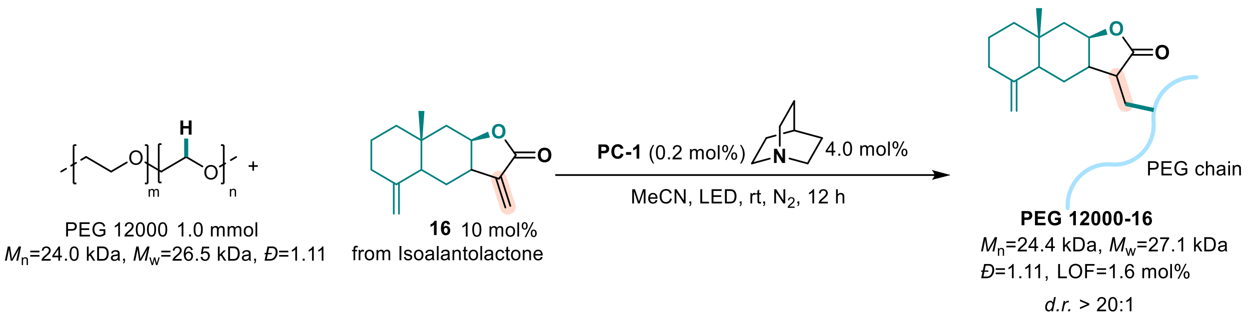

To a 4 mL vial were added **PC-1** (2.4 mg, 0.002 mmol), quinuclidine (4.3 mg, 0.04 mmol), PEG 12000 (44.3 mg, 1.0 mmol), **16** (23.5 mg, 0.1 mmol), and MeCN (2.0 mL) in an N_2 glovebox. The vial was then sealed and transferred out of the glovebox. Under irradiation at 460 nm LEDs, the resulting mixture was stirred for 12 hours at rt. Evaporation and the resulting mixture was precipitated 3 times using DCM and Et_2O (DCM/Et_2O = 1:8) until the small molecules were removed completely. The compound **PEG 12000-16** was obtained as a yellow solid (52.9 mg, LOF = 1.6 mol%, *d.r.* > 20:1). The level of functionalization was determined by ^{1}H NMR. The M_n and $Đ$ values were determined by GPC.

^{1}H NMR (400 MHz, $CDCl_3$) δ 4.78-4.70 (m, 0.94 H), 4.50-4.31 (m, **2.00 H**), 3.85-3.28 (m, **251.60 H**).^{13}C NMR (151 MHz, $CDCl_3$) spectrum is very complicated, which is attached in the spectra part directly. **IR ν (neat, cm^{-1})** 2871, 1759, 1351, 1104.

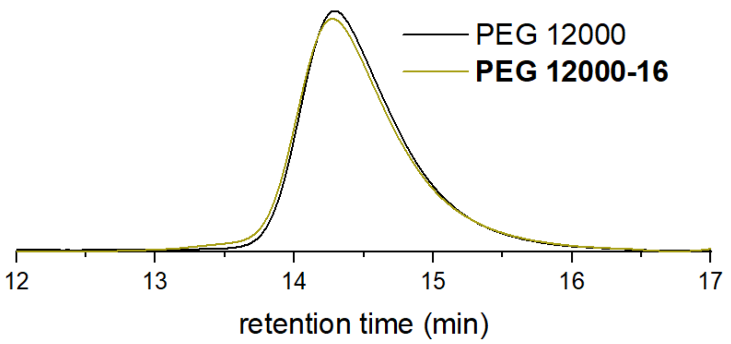

Entry	LOF (mol%)	M_w (g/mol)	M_n (g/mol)	$Đ$
1	0	26529	23981	1.10
2	1.6	27098	24373	1.11

Supplementary Figure 24. GPC of PEG 12000 and **PEG 12000-16**

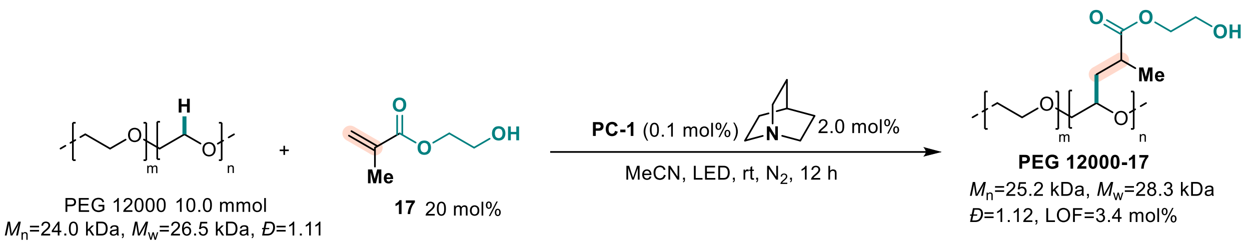

To a 100 mL vial were added **PC-1** (11.3 mg, 0.01 mmol), quinuclidine (22.3 mg, 0.2 mmol), PEG 12000 (441.2 mg, 10.0 mmol), **17** (242 μL, *d* = 1.073 g/mL, 260.0 mg, 2.0 mmol), and MeCN (20.0 mL) in an N_2 glovebox. The vial was then sealed and transferred out of the glovebox. Under irradiation at 460 nm LEDs, the resulting mixture was stirred for 12 hours at rt. Evaporation and the resulting mixture was precipitated 3 times using DCM and Et_2O (DCM/Et_2O = 1:8) until the small molecules were removed completely. The compound **PEG 12000-17** was obtained as a yellow solid (483.0 mg, LOF = 3.4 mol%). The level of functionalization was determined by ^{1}H NMR. The M_n and $Đ$ values were determined by GPC.

^{1}H NMR (400 MHz, $CDCl_3$) δ 4.28-4.00 (m, **2.00 H**), 3.85-3.28 (m, **117.67 H**), 1.25-1.12 (m, 3.32 H).^{13}C NMR (151 MHz, $CDCl_3$) spectrum is very complicated, which is attached in the spectra part directly. **IR ν (neat, cm^{-1})** 2884, 1723, 1457, 1104.

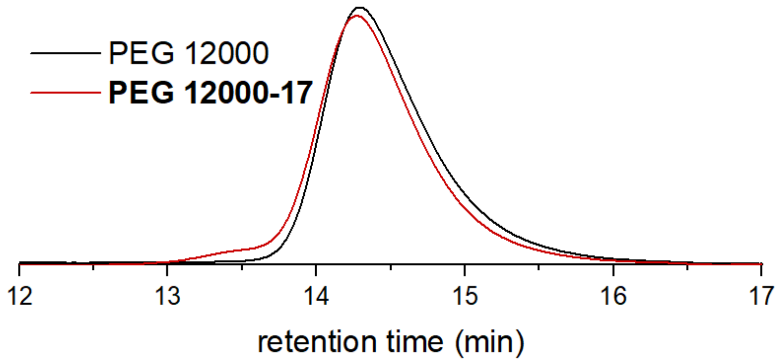

Entry	LOF (mol%)	M_w (g/mol)	M_n (g/mol)	$Đ$
1	0	26529	23981	1.10
2	3.4	28310	25190	1.12

Supplementary Figure 25. GPC of PEG 12000 and **PEG 12000-17**

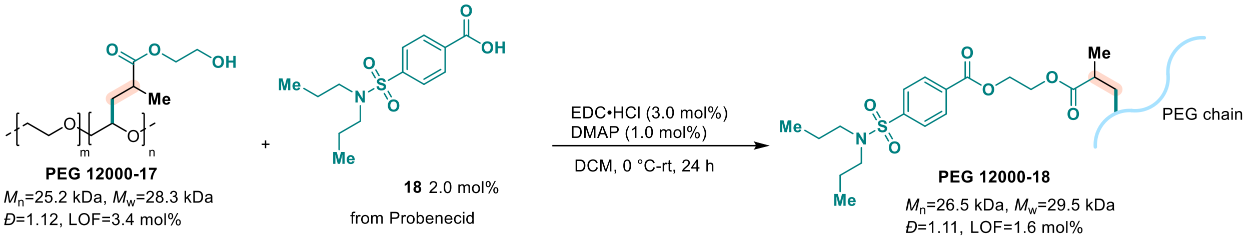

To a 20 mL vial were added **PEG 12000-17** (88.6 mg, 2.0 mmol), **18** (11.5 mg, 0.04 mmol), DMAP (2.7 mg, 0.02 mmol), and DCM (3.0 mL) at 0 °C. And then, 1-(3-dimethylaminopropyl)-3-ethylcarbodiimide hydrochloride EDC·HCl (11.8 mg, 0.06 mmol) in 1 mL DCM was dropwise added to solution. Worming to room temperature, the resulting mixture was stirred for 24 hours. Evaporation and the resulting mixture was precipitated 3 times using DCM and Et_2O (DCM/Et_2O = 1:8) until the small molecules were removed completely. The compound **PEG 12000-18** was obtained as a yellow solid (106.2 mg, LOF = 1.6 mol%). The level of functionalization was determined by ^{1}H NMR. The M_n and $Đ$ values were determined by GPC.

^{1}H NMR (400 MHz, $CDCl_3$) δ 8.25-8.10 (m, **1.00 H**), 7.80-7.75 (m, 0.84 H), 3.85-3.28 (m, **124.42 H**).^{13}C NMR (151 MHz, $CDCl_3$) spectrum is very complicated, which is attached in the spectra part directly. **IR ν (neat, cm^{-1})** 3434, 2878, 1731, 1107.

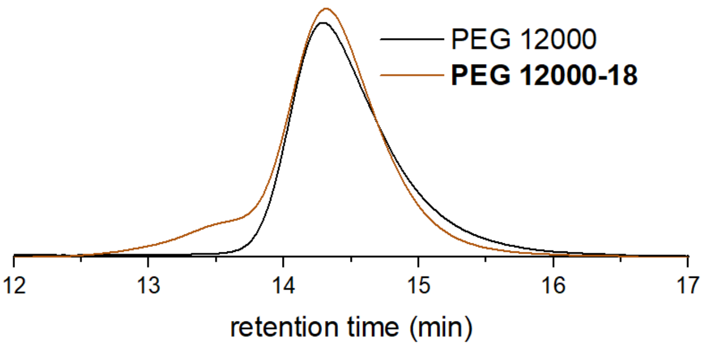

Entry	LOF (mol%)	M_w (g/mol)	M_n (g/mol)	$Đ$
1	0	26529	23981	1.10
2	1.6	29515	26493	1.11

Supplementary Figure 26. GPC of **PEG 12000-17** and **PEG 12000-18**

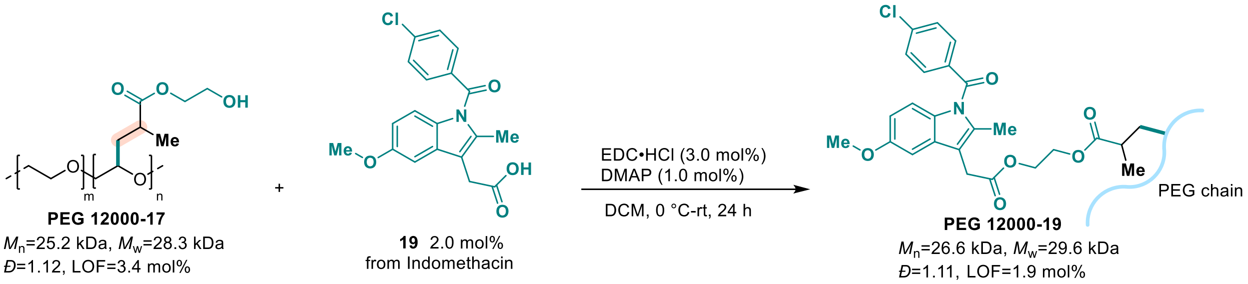

To a 20 mL vial were added **PEG 12000-17** (88.3 mg, 2.0 mmol), **19** (14.5 mg, 0.04 mmol), DMAP (2.7 mg, 0.02 mmol), and DCM (3.0 mL) at 0 °C. And then, 1-(3-dimethylaminopropyl)-3-ethylcarbodiimide hydrochloride EDC·HCl (11.2 mg, 0.06 mmol) in 1 mL DCM was dropwise added to solution. Worming to room temperature, the resulting mixture was stirred for 24 hours. Evaporation and the resulting mixture was precipitated 3 times using DCM and Et_2O (DCM/Et_2O = 1:8) until the small molecules were removed completely. The compound **PEG 12000-19** was obtained as a yellow solid (95.5 mg, LOF = 1.9 mol%). The level of functionalization was determined by ^{1}H NMR. The M_n and $Đ$ values were determined by GPC.

^{1}H NMR (400 MHz, $CDCl_3$) δ 7.75-7.61 (m, **1.00 H**), 7.50-7.35 (m, 0.93 H), 3.85-3.28 (m, **104.96 H**).^{13}C NMR (151 MHz, $CDCl_3$) spectrum is very complicated, which is attached in the spectra part directly. **IR ν (neat, cm^{-1})** 3406, 2871, 1731, 1114.

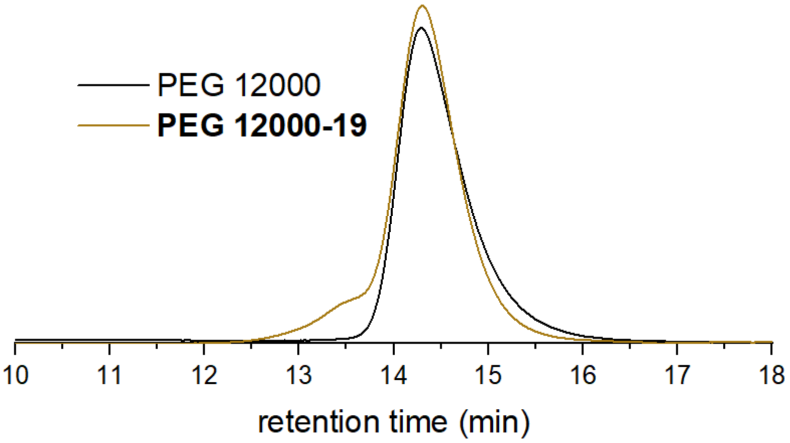

Entry	LOF (mol%)	M_w (g/mol)	M_n (g/mol)	$Đ$
1	0	26529	23981	1.10
2	1.9	29630	26621	1.11

Supplementary Figure 27. GPC of PEG 12000 and **PEG 12000-19**

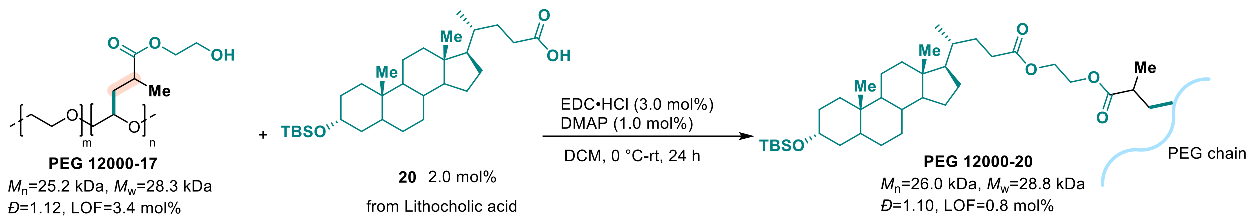

To a 20 mL vial were added **PEG 12000-17** (88.1 mg, 2.0 mmol), **20** (19.7 mg, 0.04 mmol), DMAP (2.7 mg, 0.02 mmol), and DCM (3.0 mL) at 0 °C. And then, 1-(3-dimethylaminopropyl)-3-ethylcarbodiimide hydrochloride EDC·HCl (11.3 mg, 0.06 mmol) in 1 mL DCM was dropwise added to solution. Worming to room temperature, the resulting mixture was stirred for 24 hours. Evaporation and the resulting mixture was precipitated 3 times using DCM and Et_2O (DCM/Et_2O = 1:8) until the small molecules were removed completely. The compound **PEG 12000-20** was obtained as a yellow solid (124.9 mg, LOF = 0.8 mol%). The level of functionalization was determined by ^{1}H NMR. The M_n and $Đ$ values were determined by GPC.

^{1}H NMR (400 MHz, $CDCl_3$) δ 3.85-3.28 (m, **479.03 H**), 0.83-0.70 (m, **14.67 H**), 0.65-0.50 (m, **6.00 H**).^{13}C NMR (151 MHz, $CDCl_3$) spectrum is very complicated, which is attached in the spectra part directly. **IR ν (neat, cm^{-1})** 3437, 2871, 1731, 1108.

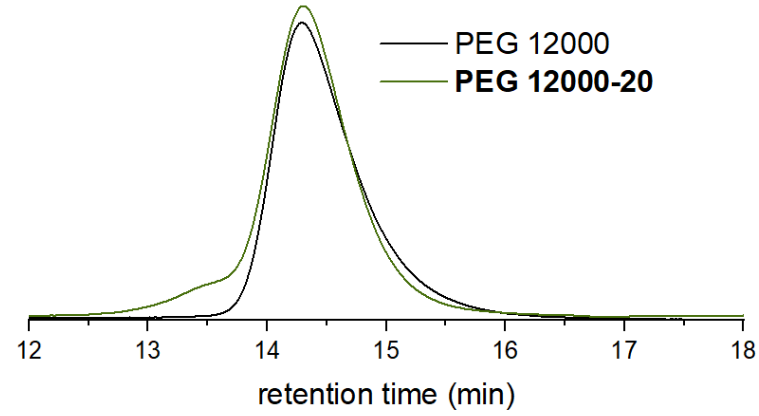

Entry	LOF (mol%)	M_w (g/mol)	M_n (g/mol)	$Đ$
1	0	26529	23981	1.10
2	0.8	28755	26036	1.10

Supplementary Figure 28. GPC of PEG 12000 and **PEG 12000-20**

(8) Modular synthesis of multifunctional polymeric carrier template

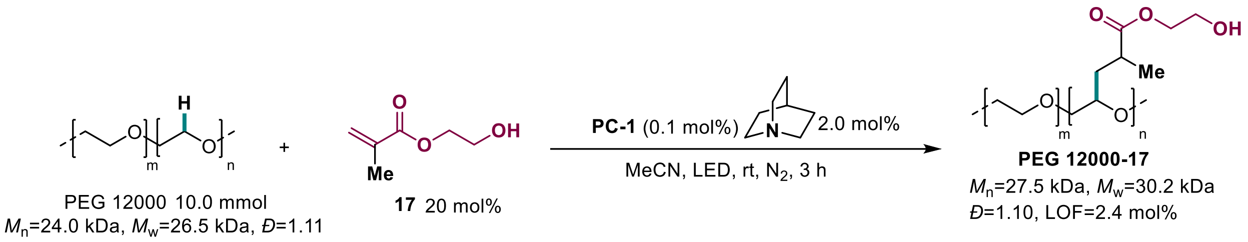

To a 100 mL vial were added **PC-1** (11.2 mg, 0.01 mmol), quinuclidine (22.3 mg, 0.2 mmol), PEG 12000 (444.2 mg, 10.0 mmol), **17** (242 µL, d = 1.073 g/mL, 260.0 mg, 2.0 mmol), and MeCN (20.0 mL) in an N_2 glovebox. The vial was then sealed and transferred out of the glovebox. Under irradiation at 460 nm LEDs, the resulting mixture was stirred for 3 hours at rt. Evaporation and the resulting mixture was precipitated 3 times using DCM and Et_2O (DCM/Et_2O = 1:8) until the small molecules were removed completely. The compound **PEG 12000-17** was obtained as a yellow solid (444.1 mg, LOF = 2.4 mol%, for **PEG 12000-17**). The level of functionalization was determined by ^{1}H NMR. The M_n and $Đ$ values were determined by GPC.

^{1}H NMR (400 MHz, $CDCl_3$) δ 3.85-3.28 (m, **170.00 H**), 1.25-1.12 (m, **3.00 H**).

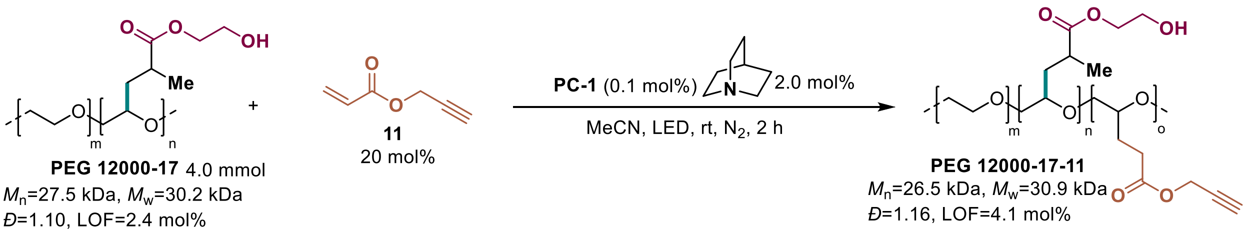

To a 25 mL vial were added **PC-1** (4.4 mg, 0.004 mmol), quinuclidine (8.8 mg, 0.08 mmol), **PEG 12000-17** (176.2 mg, 4.0 mmol), **11** (90 µL, d = 0.977 g/mL, 88.0 mg, 0.8 mmol), and MeCN (8.0 mL) in an N_2 glovebox. The vial was then sealed and transferred out of the glovebox. Under irradiation at 460 nm LEDs, the resulting mixture was stirred for 2 hours at rt. Evaporation and the resulting mixture was precipitated 3 times using DCM and Et_2O (DCM/Et_2O = 1:8) until the small molecules were removed completely. The compound **PEG 12000-17-11** was obtained as a yellow solid (195.9 mg, LOF = 4.1 mol%, for **PEG 12000-11**). The level of functionalization was determined by ^{1}H NMR. The M_n and $Đ$ values were determined by GPC.

^{1}H NMR (400 MHz, $CDCl_3$) δ 4.75-4.65 (m, **2.00 H**), 3.85-3.28 (m, **96.48 H**).

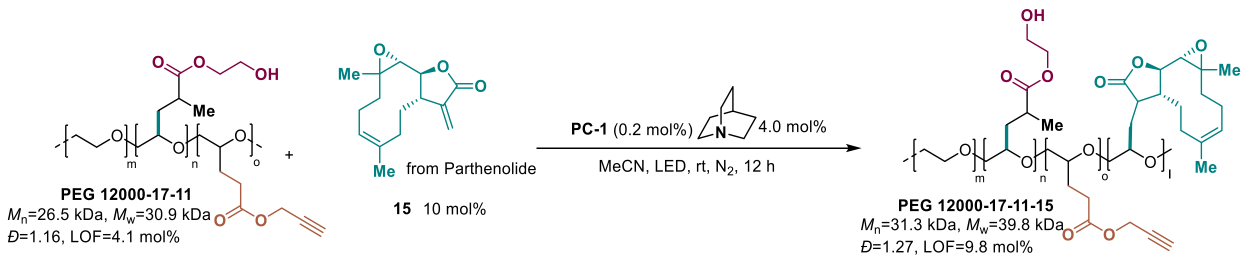

To a 25 mL vial were added **PC-1** (2.2 mg, 0.002 mmol), quinuclidine (4.6 mg, 0.04 mmol), **PEG 12000-17-11** (44.0 mg, 1.0 mmol), **15** (24.9 mg, 0.1 mmol), and MeCN (2.0 mL) in an N_2 glovebox. The vial was then sealed and transferred out of the glovebox. Under irradiation at 460 nm LEDs, the resulting mixture was stirred for 12 hours at rt. Evaporation and the resulting mixture was precipitated 3 times using DCM and Et_2O (DCM/Et_2O = 1:8) until the small molecules were removed completely. The compound **PEG 12000-17-11** was obtained as a yellow solid (45.7 mg, LOF = 9.8 mol%, for **PEG 12000-15**). The level of functionalization was determined by ^{1}H NMR. The M_n and $Đ$ values were determined by GPC.

^{1}H NMR (400 MHz, CDCl$_3$) δ 5.30-5.12 (m, **1.00 H**), 3.85-3.28 (m, **40.98 H**).

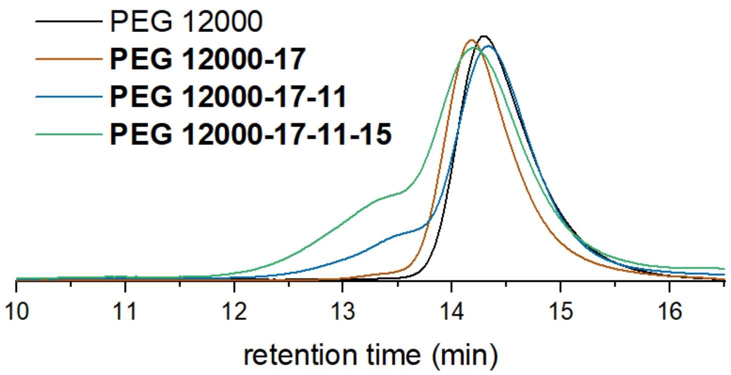

Entry	LOF (mol%)	M_w (g/mol)	M_n (g/mol)	$Đ$
1	0	26529	23981	1.10
2	2.4	30203	27509	1.10
3	4.1	30931	26471	1.16
4	9.8	39769	31332	1.27

Supplementary Figure 29. GPC of PEG 12000, **PEG 12000-17**, **PEG 12000-17-11**, and **PEG 12000-17-11-15**

(9) PEGylation of BSA with MeO-PEG-OMe 2000-17

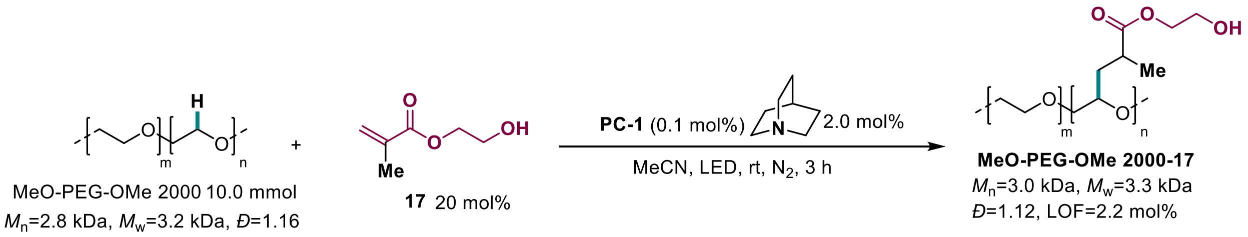

To a 100 mL vial were added **PC-1** (11.2 mg, 0.01 mmol), quinuclidine (22.5 mg, 0.2 mmol), MeO-PEG-OMe 2000 (444.0 mg, 10.0 mmol), **17** (242 μL, *d* = 1.073 g/mL, 260.0 mg, 2.0 mmol), and MeCN (20.0 mL) in an N_2 glovebox. The vial was then sealed and transferred out of the glovebox. Under irradiation at 460 nm LEDs, the resulting mixture was stirred for 3 hours at rt. Evaporation and Flash chromatography on silica gel (DCM to DCM/MeOH = 10/1) afforded **MeO-PEG-OMe 2000-17**: yellow solid (390.9 mg, LOF = 2.2 mol%). The level of functionalization was determined by ^{1}H NMR. The M_w, M_n, and $Đ$ values were determined by GPC.

^{1}H NMR (400 MHz, $CDCl_3$) δ 4.62-4.00 (m, **2.00 H**), 4.05-3.25 (m, **183.74 H**), 1.24-1.05 (m, 3.05 H), The ^{13}C NMR (151 MHz, $CDCl_3$) spectrum is very complicated, which is attached in the spectra part directly. **IR ν (neat, cm^{-1})** 3472, 2872, 1727, 1118.

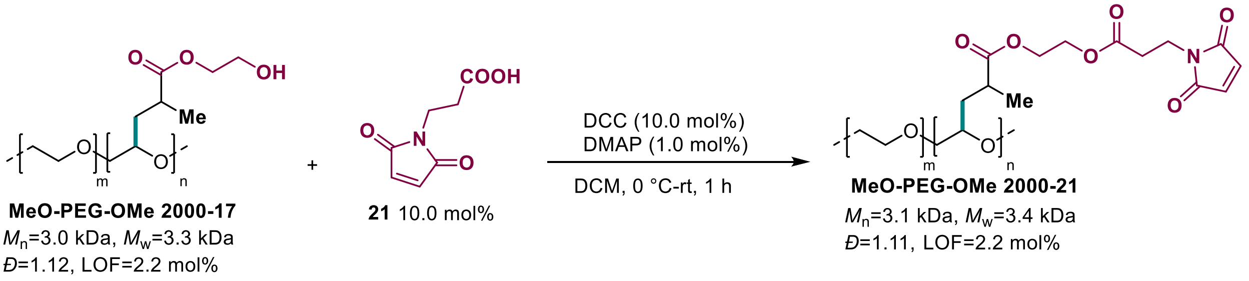

To a 20 mL vial were added, **21** (19.7 mg, 0.04 mmol), dicyclohexylcarbodiimide DCC (82.8 mg, 0.4 mmol), DMAP (5.3 mg, 0.04 mmol), and DCM (10.0 mL) at 0 °C. And then, **MeO-PEG-OMe 2000-17** (176.3 mg, 4.0 mmol) in 5 mL DCM was dropwise added to solution. Worming to room temperature, the resulting mixture was stirred for 1 hours. To this reaction vessel was added 20 mL a saturated aqueous solution of Na_2CO_3, and the aqueous layer was extracted with DCM (20 mL × 2) and washed with a saturated aqueous solution of Na_2CO_3 (20 mL × 2). The combined organic layer was dried over anhydrous Na_2SO_4, filtered, and evaporated. Flash chromatography on silica gel (DCM to DCM/MeOH = 10/1) afforded **MeO-PEG-OMe 2000-21**: yellow solid (83.6 mg, LOF = 2.2 mol%). The level of functionalization was determined by ^{1}H NMR. The M_n and $Đ$ values were determined by GPC.

^{1}H NMR (400 MHz, CDCl$_3$) δ 6.75-5.60 (m, **2.00 H**), 4.25-4.00 (m, 4.18 H), 4.95-3.32 (m, **179.84 H**), 2.68-2.54 (m, 2.71 H), 1.24-1.05 (m, 3.12 H). The ^{13}C NMR (151 MHz, CDCl$_3$) spectrum is very complicated, which is attached in the spectra part directly. **IR ν (neat, cm^{-1})** 2871, 1734, 1710, 1102.

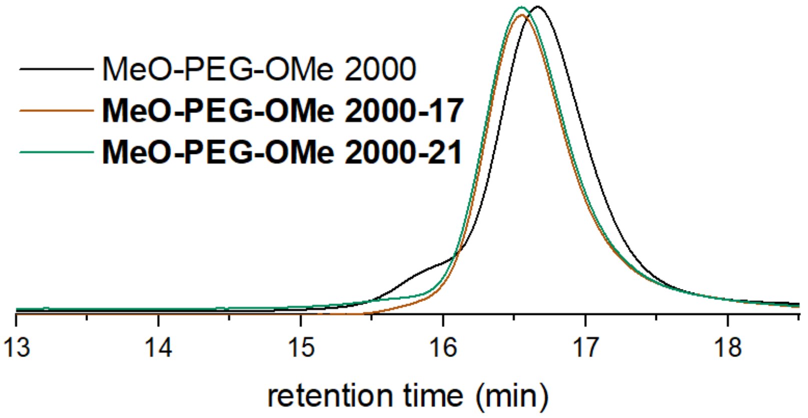

Entry	LOF (mol%)	M_w (g/mol)	M_n (g/mol)	$Đ$
1	0	3220	2775	1.16
2	2.2	3335	2965	1.12
3	2.2	3402	3068	1.11

Supplementary Figure 30. GPC of MeO-PEG-OMe 2000, **MeO-PEG-OMe 2000-17**, and **MeO-PEG-OMe 2000-21**

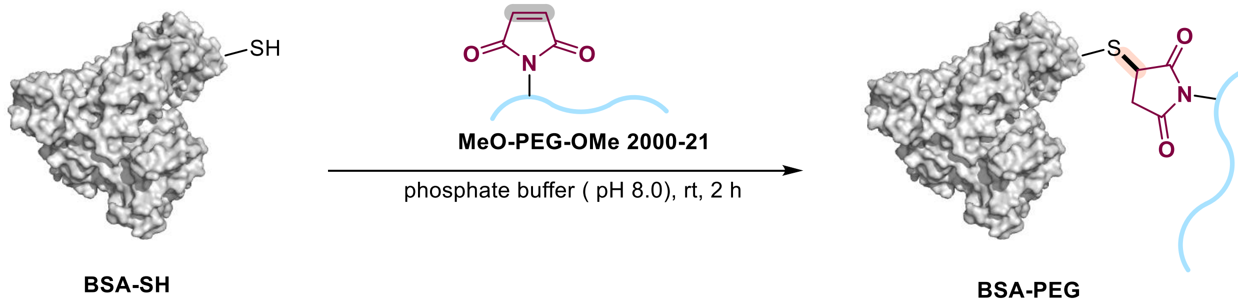

To a 2.0 mL vial were added 0.20 mL BSA-SH (4.2 mg, 0.00006 mmol, 20.86 mg/mL in H_2O), **MeO-PEG-OMe 2000-21** (1.4 mg, 0.0006 mmol, 0.2 mL phosphate buffer, pH 8.0) at rt. The resulting mixture was stirred for 2 hours. The product BSA-PEG was detected by SDS-PAGE. And the band of conjugate is reasonable by comparing to reported literature (*J. Am. Chem. Soc.* **2012**, *134*, 8474−8479).

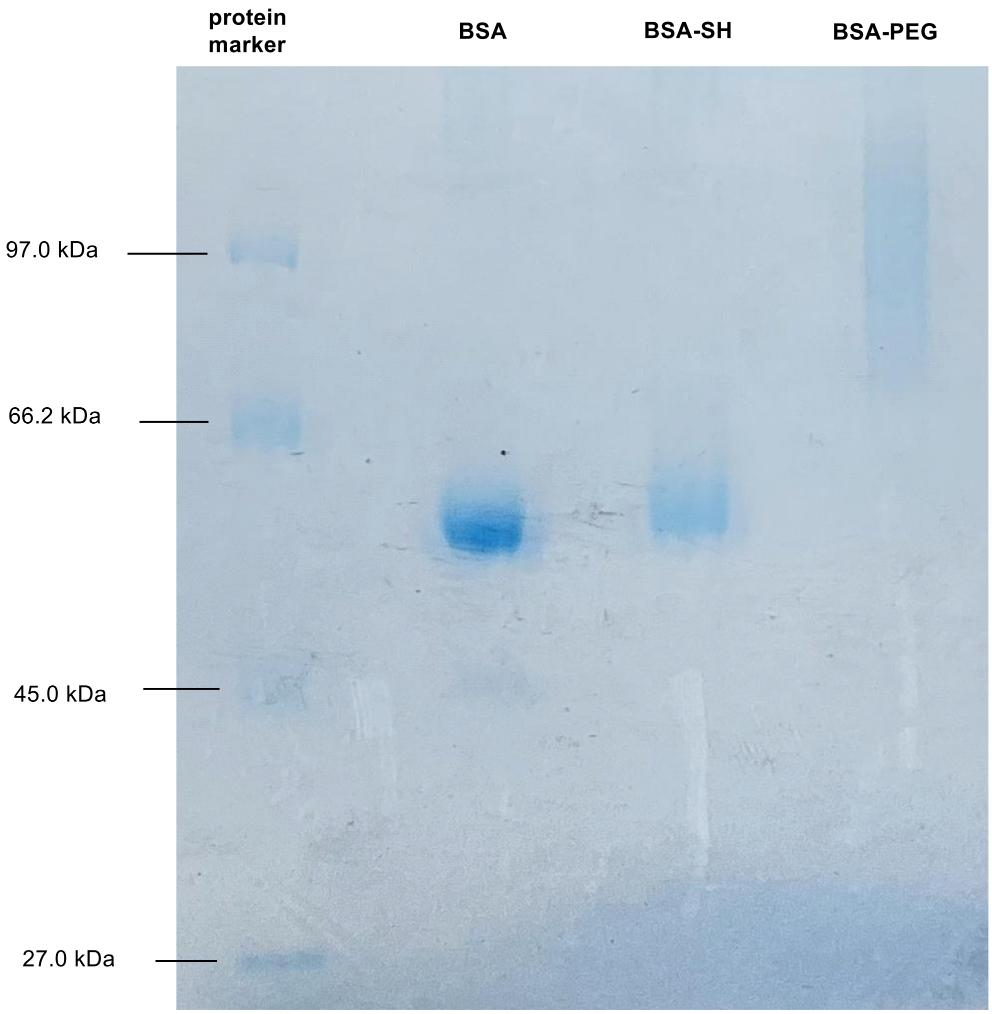

Supplementary Figure 31. SDS-PAGE of protein marker, BSA, **BSA-SH**, and **BSA-PEG**.

(10) Mechanism investigation of C–H bond functionalization of PEGs with acylate.

(10-1) Controlled reaction of dioxane, DME, PEG 2000, and 18-crown-6 with acylate **1**.

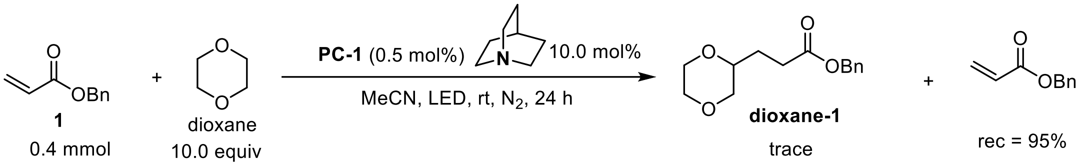

To a 4 mL vial were added **PC-1** (2.2 mg, 0.002 mmol), quinuclidine (4.4 mg, 0.04 mmol), **1** (60.0 μL, *d* = 1.080 g/mL, 64.8 mg, 0.4 mmol), dioxane (0.34 mL, *d* = 1.034 g/mL, 352.0 mg, 4.0 mmol), and MeCN (4.0 mL) in an N_2 glovebox. The vial was then sealed and transferred out of the glovebox. Under irradiation at 460 nm LEDs, the resulting mixture was stirred for 24 hours at rt. Evaporation and flash chromatography on silica gel afforded **dioxane-1** (**trace**), **1** (61.6 mg, recovery = 95%) (eluent: PE/EA = 10/1).

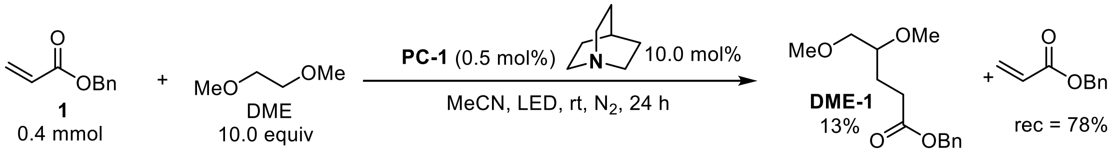

To a 4 mL vial were added **PC-1** (2.2 mg, 0.002 mmol), quinuclidine (4.4 mg, 0.04 mmol), **1** (60.0 μL, *d* = 1.080 g/mL, 64.8 mg, 0.4 mmol), **DME-1** (0.42 mL, *d* = 0.867 g/mL, 360.0 mg, 4.0 mmol), and MeCN (4.0 mL) in an N_2 glovebox. The vial was then sealed and transferred out of the glovebox. Under irradiation at 460 nm LEDs, the resulting mixture was stirred for 24 hours at rt. Evaporation and flash chromatography on silica gel afforded **DME-1** (13.1 mg, **13%**), **1** (50.8 mg, recovery = 78%) (eluent: PE/EA = 10/1): **DME-1** colorless oil. **^{1}H NMR (400 MHz, Chloroform-*d*)** δ 7.42-7.28 (m, 5 H), 5.12 (s, 2 H), 3.59-3.44 (m, 2 H), 3.41-3.32 (m, 7 H), 2.46 (t, *J* = 7.5 Hz, 2 H), 2.02-1.77 (m, 2 H).**^{13}C NMR (101 MHz, Chloroform-*d*)** δ 173.4, 136.0, 128.5, 128.2, 128.2, 78.8, 74.1, 71.9, 70.1 (d, *J* = 12.5 Hz), 59.2, 59.0, 57.6, 30.9, 30.0, 26.5, 24.9. **IR ν (neat, cm^{-1})** 2923, 1736, 1455, 1114. **HRMS (ESI) m/z**: $[M + Na]^+$ Calcd for $C_{14}H_{20}O_4Na$ 275.1254; found 275.1258.

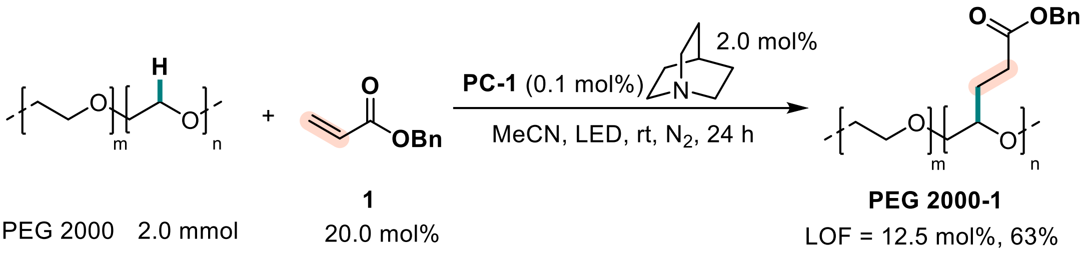

To a 4 mL vial were added **PC-1** (2.2 mg, 0.002 mmol), quinuclidine (4.4 mg, 0.04 mmol), PEG 2000 (88.0 mg, 2.0 mmol), **1** (60.0 μL, *d* = 1.080 g/mL, 64.8 mg, 0.4 mmol), and MeCN (4.0 mL) in an N_2 glovebox. The vial was then sealed and transferred out of the glovebox. Under irradiation at 460 nm LEDs, the resulting mixture was stirred for 24 hours at rt. Evaporation and flash chromatography on silica gel (DCM to DCM/MeOH = 10/1) afforded **PEG 2000-1**: yellow oil (72.5 mg, LOF = 12.5 mol%, **63%**). The level of functionalization was determined by ^{1}H NMR.

^{1}H NMR (400 MHz, $CDCl_3$) δ 7.50-7.27 (m, 5.36 H), 5.25-5.00 (m, **2.00 H**), 4.05-3.25 (m, **32.25 H**). The ^{13}C NMR (151 MHz, $CDCl_3$) spectrum is very complicated, which is attached in the spectra part directly. **IR ν (neat, cm^{-1})** 3439, 2872, 1730, 1104.

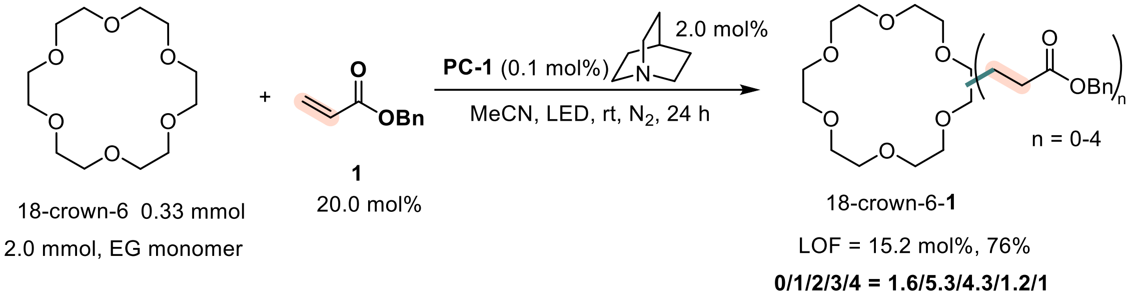

To a 4 mL vial were added **PC-1** (2.3 mg, 0.002 mmol), quinuclidine (4.6 mg, 0.04 mmol), 18-crown-6 (88.6 mg, 0.33 mmol), **1** (60.0 μL, *d* = 1.080 g/mL, 64.8 mg, 0.4 mmol), and MeCN (4.0 mL) in an N_2 glovebox. The vial was then sealed and transferred out of the glovebox. Under irradiation at 460 nm LEDs, the resulting mixture was stirred for 24 hours at rt. Evaporation and flash chromatography on silica gel (DCM to DCM/MeOH = 20/1) afforded 18-crown-6-**1**: yellow oil (121.3 mg, LOF = 15.2 mol%, **76%**). The level of functionalization was determined by ^{1}H NMR.

^{1}H NMR (400 MHz, $CDCl_3$) δ 7.50-7.27 (m, 5.15 H), 5.25-5.00 (m, **2.00 H**), 3.82-3.25 (m, **26.27 H**). The ^{13}C NMR (151 MHz, $CDCl_3$) spectrum is very complicated, which is attached in the spectra part directly. **IR ν (neat, cm^{-1})** 2909, 1731, 1455, 1114. **HRMS (ESI) m/z**: n = 0, [M + Na]$^+$ Calcd for $C_{12}H_{24}O_6Na$ 287.1465; found 287.1446. n = 1, [M + Na]$^+$ Calcd for $C_{22}H_{34}O_8Na$

449.2146; found 449.2141. n = 2, $[M + Na]^+$ Calcd for $C_{32}H_{44}O_{10}Na$ 611.2827; found 611.2825. n = 3, $[M + Na]^+$ Calcd for $C_{42}H_{54}O_{12}Na$ 773.3507; found 773.3509. n = 4, $[M + Na]^+$ Calcd for $C_{52}H_{64}O_{14}Na$ 935.4188; found 935.4184. The ratio of n = 0, n = 1, n = 2, n = 3, and n = 4 C–H alkylation was determined as 1.6:5.3:4.3:1.2:1 by analyzing the LC-HRMS.

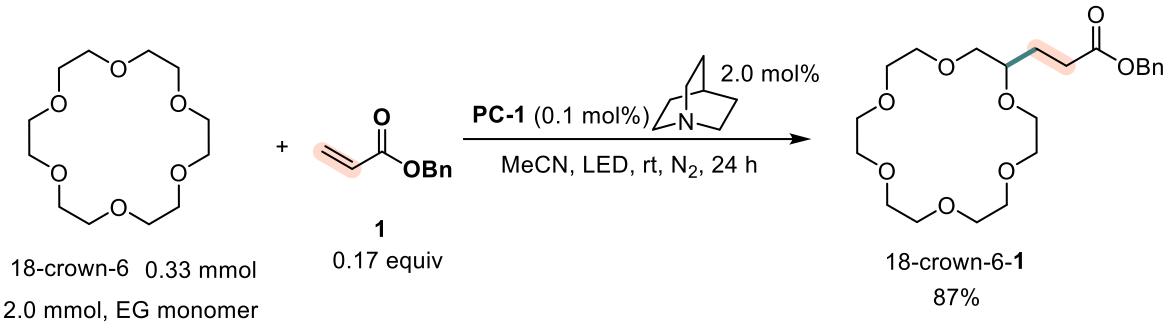

To a 4 mL vial were added **PC-1** (2.3 mg, 0.002 mmol), quinuclidine (4.5 mg, 0.04 mmol), 18-crown-6 (88.2 mg, 0.33 mmol), **1** (9.0 µL, *d* = 1.080 g/mL, 9.7 mg, 0.06 mmol), and MeCN (4.0 mL) in an N_2 glovebox. The vial was then sealed and transferred out of the glovebox. Under irradiation at 460 nm LEDs, the resulting mixture was stirred for 24 hours at rt. Evaporation and flash chromatography on silica gel (PE to PE/acetone = 3/1) afforded 18-crown-6-**1**: oil (22.3 mg, 87%).

^{1}H NMR (400 MHz, $CDCl_3$) δ 7.50-7.27 (m, 5 H), 5.11 (s, 2 H), 3.82-3.25 (m, 22 H), 2.48 (td, J_1 = 7.6, J_2 = 2.9 Hz, 2 H), 1.95-1.78 (m, 2 H). **The ^{13}C NMR (151 MHz, $CDCl_3$)** δ 173.4, 136.0, 128.5, 128.2, 128.2, 78.0, 73.9, 70.81, 70.78, 70.7, 70.63, 70.57, 70.55, 70.53, 70.49, 69.6, 66.1, 30.3, 26.9. **IR ν (neat, cm^{-1})** 2894, 1729, 1457, 1355. **HRMS (ESI) m/z**: $[M + Na]^+$ Calcd for $C_{22}H_{34}O_8Na$ 449.2146; found 449.2141.

(10-2) The reaction of different size crown ethers with acylate **1**.

The reaction of **PC-1** (2.3 mg, 0.002 mmol), quinuclidine (4.6 mg, 0.04 mmol), [12-crown-4 (88.0 mg, 2.0 mmol for mono), 15-crown-5 (88.0 mg, 2.0 mmol for mono), 18-crown-6 (88.0 mg, 2.0 mmol for mono), 21-crown-7 (88.0 mg, 2.0 mmol for mono),24-crown-8 (88.0 mg, 2.0 mmol for mono)], **1** (60.0 µL, *d* = 1.080 g/mL, 64.8 mg, 0.4 mmol), and MeCN (4.0 mL) in an N_2 glovebox. The vial was then sealed and transferred out of the glovebox. Under irradiation at 460 nm LEDs, the resulting mixture was stirred for 6 hours at rt. The level of functionalization was determined by crude ^{1}H NMR.

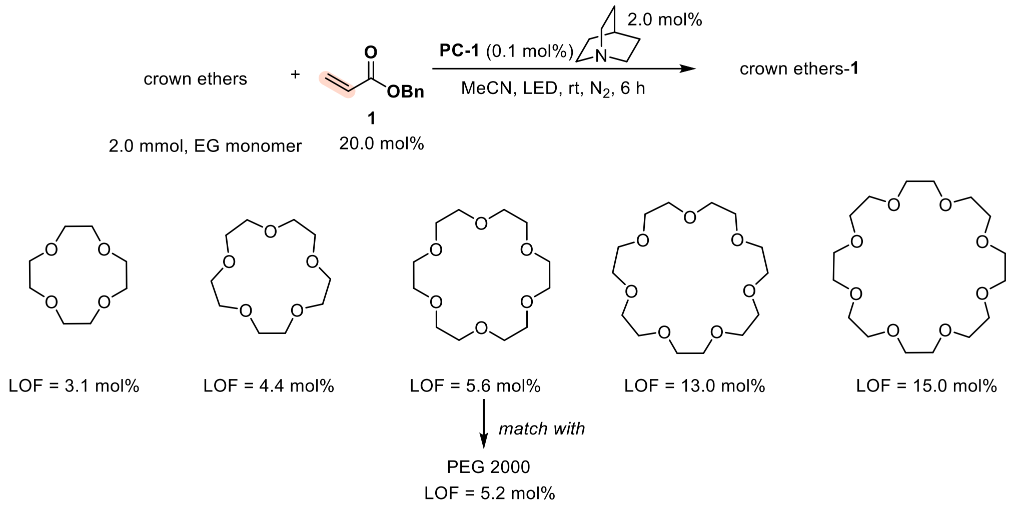

Supplementary Figure 32. The reaction of different size crown ethers with acylate **1**.

(10-3) The reaction of different dioxane and glymes with acylate **1**.

The reaction of **PC-1** (2.3 mg, 0.002 mmol), quinuclidine (4.6 mg, 0.04 mmol), [dioxane (170 μL, *d* = 1.034 g/mL, 176.0 mg, 2.0 mmol), DME (208 μL, *d* = 0.867 g/mL, 180.0 mg, 2.0 mmol), glyme-2 (284 μL, *d* = 0.944 g/mL, 268.0 mg, 2.0 mmol), glyme-3 (361 μL, *d* = 0.986 g/mL, 356.0 mg, 2.0 mmol), glyme-4 (444 μL, *d* = 1.000 g/mL, 444.0 mg, 2.0 mmol)], **1** (60.0 μL, *d* = 1.080 g/mL, 64.8 mg, 0.4 mmol), and MeCN (4.0 mL) in an N_2 glovebox. The vial was then sealed and transferred out of the glovebox. Under irradiation at 460 nm LEDs, the resulting mixture was stirred for 6 hours at rt. The level of functionalization was determined by crude ^{1}H NMR.

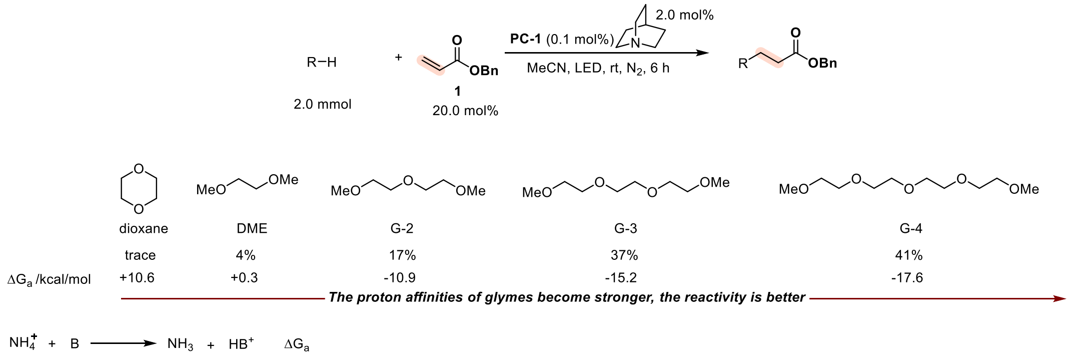

Supplementary Figure 33. The reaction of different dioxane and glymes with acylate **1**.

(10-4) Controlled reaction of dioxane, and PEG 2000 with benzylidenemalononitrile

Comparing with iron system, reaction of dioxane, and PEG 2000 with benzylidenemalononitrile had very poor reactivity under iridium-quinuclidine condition[3].

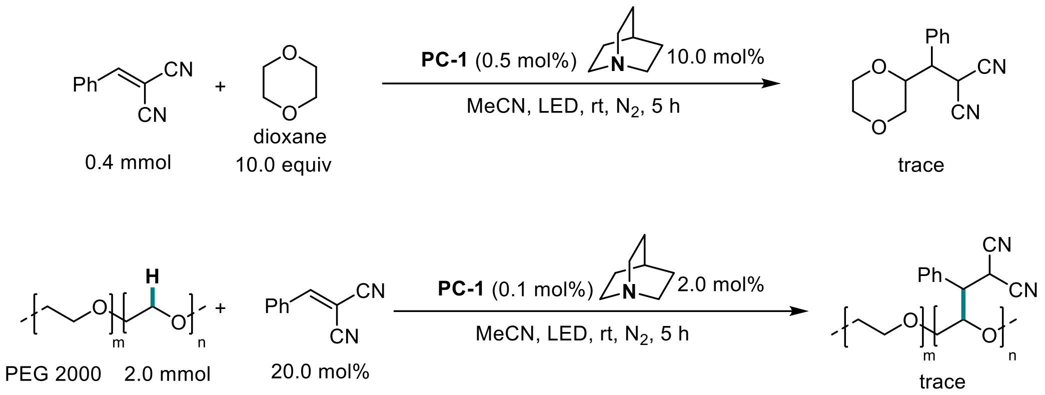

Supplementary Figure 34. The reactivity of dioxane, and PEG 2000 with benzylidenemalononitrile.

We have re-examined other tertiary amines with different alkaline. Interestingly, compared with quinuclidine **B1**, DABCO (1,4-diazabicyclo[2.2.2]octane, **B2**), triethylamine **B3**, and dimethylbenzylamine **B4** can observe the increase of LOF.

Notably, these results are compatible with the acidities of the corresponding conjugate acids (see below, details see *J. Org. Chem.* 1996, 61, 4778–4783). The stronger conjugate acids used, the higher LOF values produced. These results are also compatible with the conclusion of "the relatively lower basicity of the carbanion from 2-alkylmalononitrile might lead to the slower protonation, ….. to favorably protonate carbanion adjacent to nitrile groups."

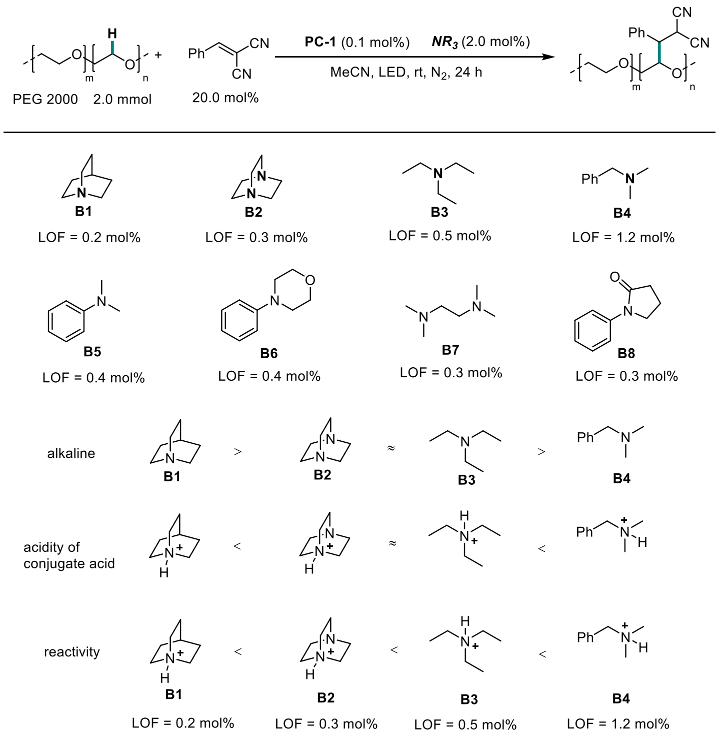

Supplementary Figure 35. The reactivity of PEG 2000 with benzylidenemalononitrile under other tertiary amines catalysis.

(10-5) pH values measure of PEG 2000, DME, dioxane, crown ethers and with quinuclidine trifluoroacetic acid salt.

To a 20 mL vial were added quinuclidine trifluoroacetic acid quinuclidine (18.0 mg, 0.08 mmol), PEG 2000 (88.0 mg, 2.0 mmol), DME (204 μL, *d* = 0.867 g/mL, 180.0 mg, 2.0 mmol), dioxane (172 μL, *d* = 1.034 g/mL, 176.0 mg, 2.0 mmol), 15-crown-5 (88.0 mg, 2.0 mmol, monomer), 18-crown-6 (88.0 mg, 2.0 mmol, monomer), 21-crown-7 (88.0 mg, 2.0 mmol, monomer), 24-crown-8 (88.0 mg, 2.0 mmol, monomer) and MeCN (4.0 mL). The pH values were measured by pH meter at 25 °C. the pH meter was calibrated by two buffers (pH 6.86 and 4.00). Each sample was detected 3 times in average.

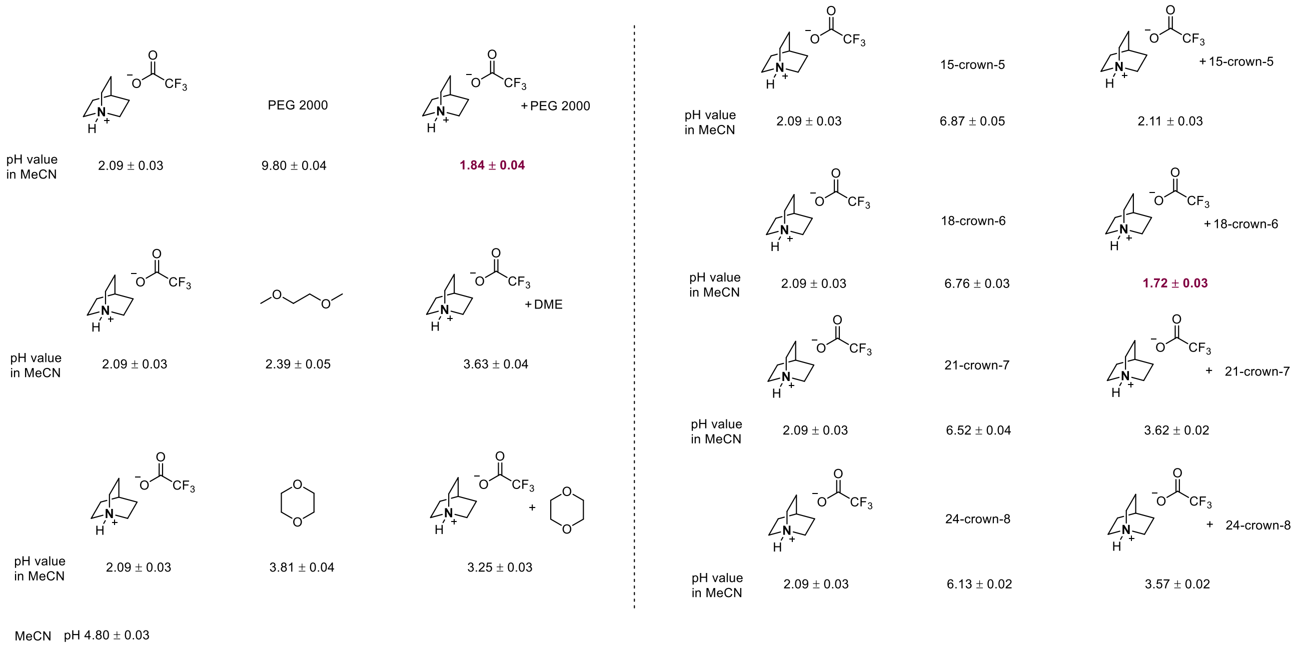

Supplementary Figure 36. pH values of various substrates.

(10-6) ^{1}H NMR of dioxane, DME, 18-crown-6, and PEG 2000 with quinuclidine trifluoroacetic acid salt.

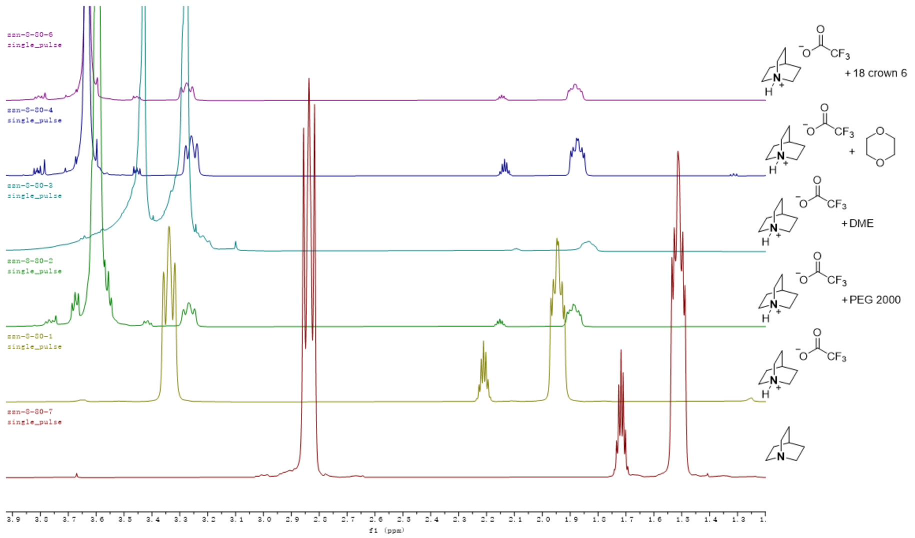

Supplementary Figure 37. ^{1}H NMR of dioxane, DME, 18-crown-6, and PEG 2000 with quinuclidine trifluoroacetic acid salt from 3.9-1.3 ppm in $CDCl_3$.

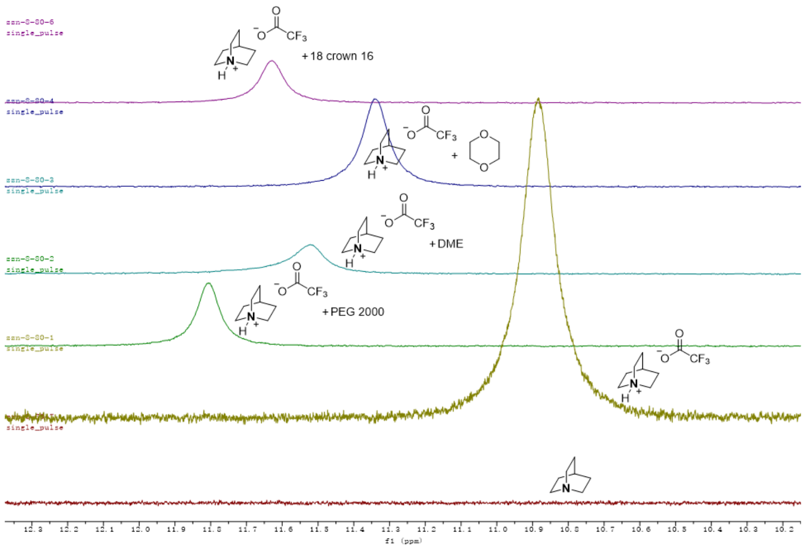

Supplementary Figure 38. ^{1}H NMR of dioxane, DME, 18-crown-6, and PEG 2000 with quinuclidine trifluoroacetic acid salt from 12.5-10.0 ppm in $CDCl_3$.

It is reasonable to predict that the interaction between [quinuclidine-H]$^{+}$(OTFA)$^{-}$ and PEG2000 or crown ether would enhance along with the increase of the relative amount of crown ether. Therefore, the experiments using different ratio of [quinuclidine-H]$^{+}$(OTFA)$^{-}$ and PEG2000 or 18-C-6 were conducted. And chemical shift value of N-H species indeed varied. **Along with the increase of amount of PEG2000 or 18-C-6, the proton at N-H bond would shifted to the**

<u>downfield of the spectra, indicating that interaction between [quinuclidine-H]$^{+}$(OTFA)$^{-}$ and PEG2000 or 18-C-6 was indeed existed and could be enhanced.</u>

This conclusion and protocol could be also proven by the known literature, such as *J. Am. Chem. Soc.* **2019**, *141*, 8868–8876.

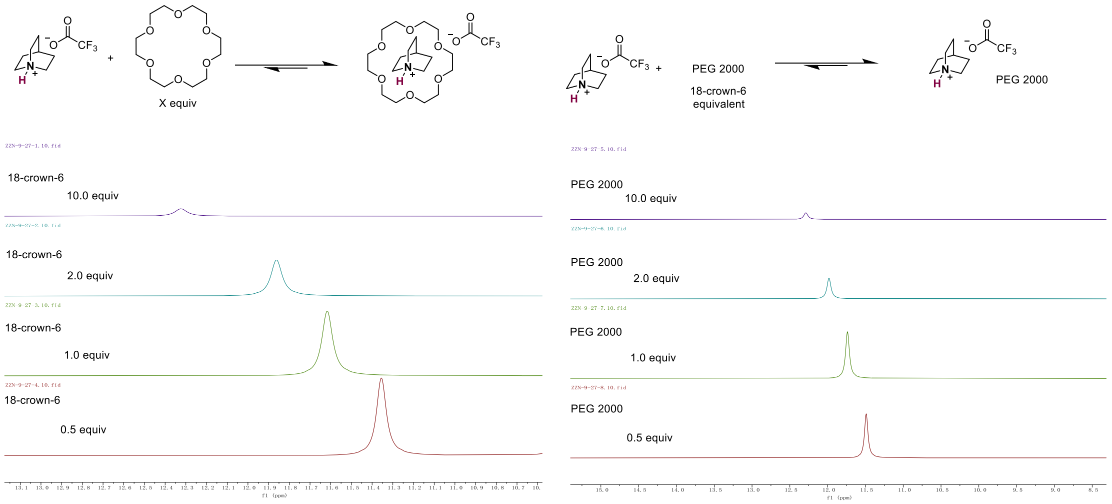

Supplementary Figure 39. ^{1}H NMR of 18-crown-6, PEG 2000 with quinuclidine trifluoroacetic acid salt in $CDCl_3$.

(10-7) Low temperature ^{1}H NMR of quinuclidine trifluoroacetic acid salt and PEG 2000 with quinuclidine trifluoroacetic acid salt.

The ionization ability of [quinuclidine-H]$^{+}$(OTFA)$^{-}$ decreased gradually along with the temperature decreased. And the chemical shift of N-H bond of [quinuclidine-H]$^{+}$(OTFA)$^{-}$ would shift to high field, which were recorded to be 12.17 (25 ℃), 12.05 (0 ℃), 11.99 (-10 ℃), and 11.95 (-20 ℃) ppm.

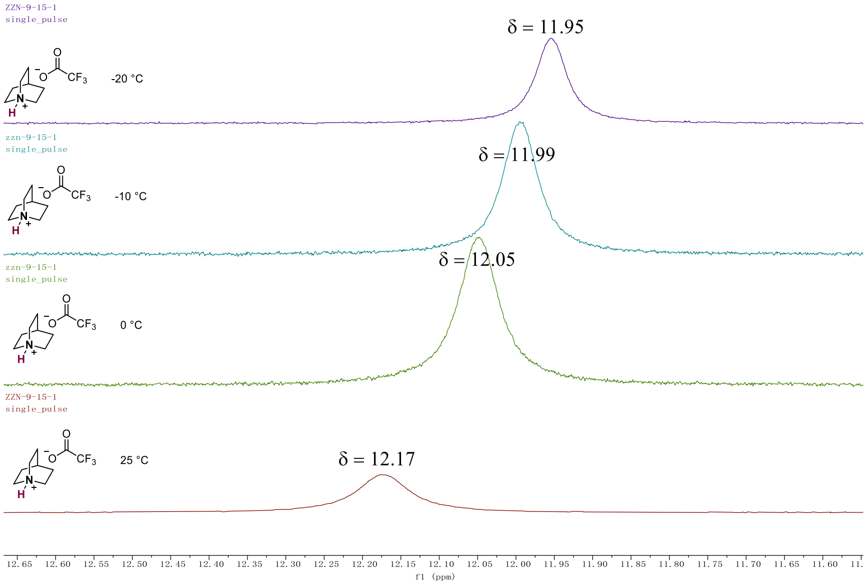

Supplementary Figure 40. Low temperature ^{1}H NMR of quinuclidine trifluoroacetic acid salt.

Alternatively, the chemical shift of N-H bond of [quinuclidine-H]$^+$(OTFA)$^-$ in the presence of PEG2000 were recorded as 12.36 (25 °C), 12.31 (0 °C), 12.29 (-10 °C), and 12.26 (-20 °C) ppm.

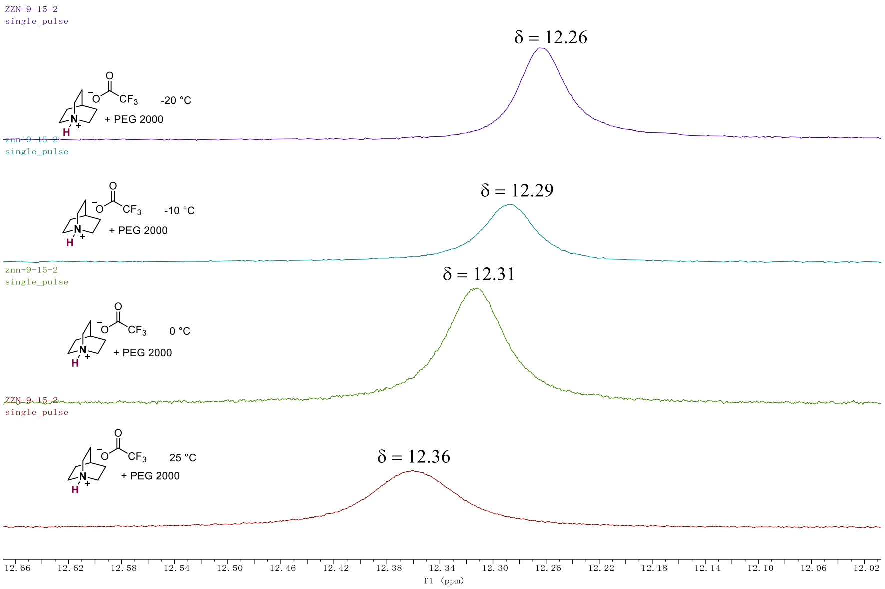

Supplementary Figure 41. Low temperature ^{1}H NMR of quinuclidine trifluoroacetic acid salt and PEG 2000.

These results clearly showed that,

(1) PEG2000 would indeed increase the ionization ability of [quinuclidine-H]$^+$(OTFA)$^-$ significantly, because the chemical shift value in the presence of PEG2000 at even -20 °C (12.26) is still larger than that in the absence of PEG2000 at room temperature (12.17).

(2) The temperature decrease affects the ionization ability of [quinuclidine-H]$^+$(OTFA)$^-$ significantly, because the chemical shift value decreased from 12.36 (25 °C) to 11.95 (-20 °C). The difference is 0.41 ppm. Alternatively, a smaller difference (0.10 ppm) is observed in the presence of PEG2000, which suggests the H-bond between PEG2000 and [quinuclidine-H]$^+$(OTFA)$^-$ might stabilize the proton significantly, resulting in that temperature decrease has less effect on the ionization ability of [quinuclidine-H]$^+$(OTFA)$^-$.

(3) The difference of chemical shift values between [quinuclidine-H]$^+$(OTFA)$^-$/PEG2000 and [quinuclidine-H]$^+$(OTFA)$^-$ increases along with the temperature decrease, which also supports the conclusion above.

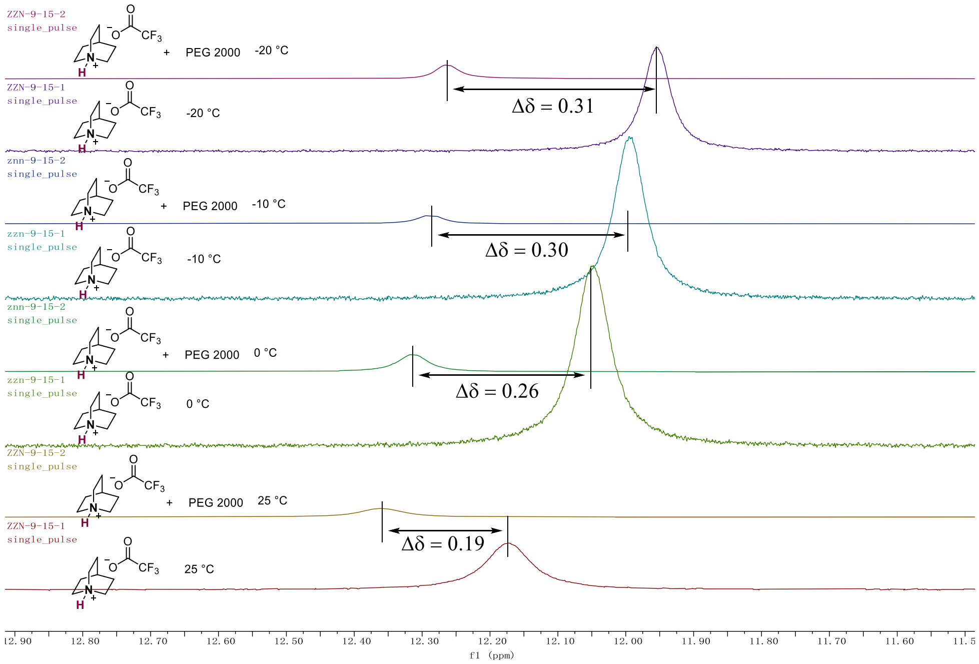

Supplementary Figure 42. Compared with low temperature ^{1}H NMR of quinuclidine trifluoroacetic acid salt and quinuclidine trifluoroacetic acid salt with PEG 2000.

Based on experiments above, we still insist on the conclusion that "**<u>the interaction between quinuclidinium ion and crown ether or polyether is weak, but it does exist</u>**".

(10-8) The HRMS analysis of 18-crown-6 and quinuclidinium ion

To further evidencing the interaction between 18-C-6 (and PEG) and quinuclidinium ion, a series of experiments were further conducted.

First, the high-resolution mass spectra (HRMS) of a mixture of 18-crown-6 and quinuclidinium ion was examined. Regretfully, there is no peak matching with [18-C-6/ quinuclidinium] ion (desired MW = 376), while quinuclidinium ion (calcd for $C_7H_{14}N^+$ 112.1121, found 112.1129), [18-crown-6 + Na^+] (calcd for $C_{12}H_{24}O_6Na^+$ 287.1465, found 287.1478), and [18-crown-6 + H^+] (calcd for $C_{12}H_{25}O_6^+$ 265.1646, found 265.1658) could be observed. This result indicated that the interaction between quinuclidinium ion and crown ether is indeed weak, which is quite compatible with the conclusion of "logK is very small".

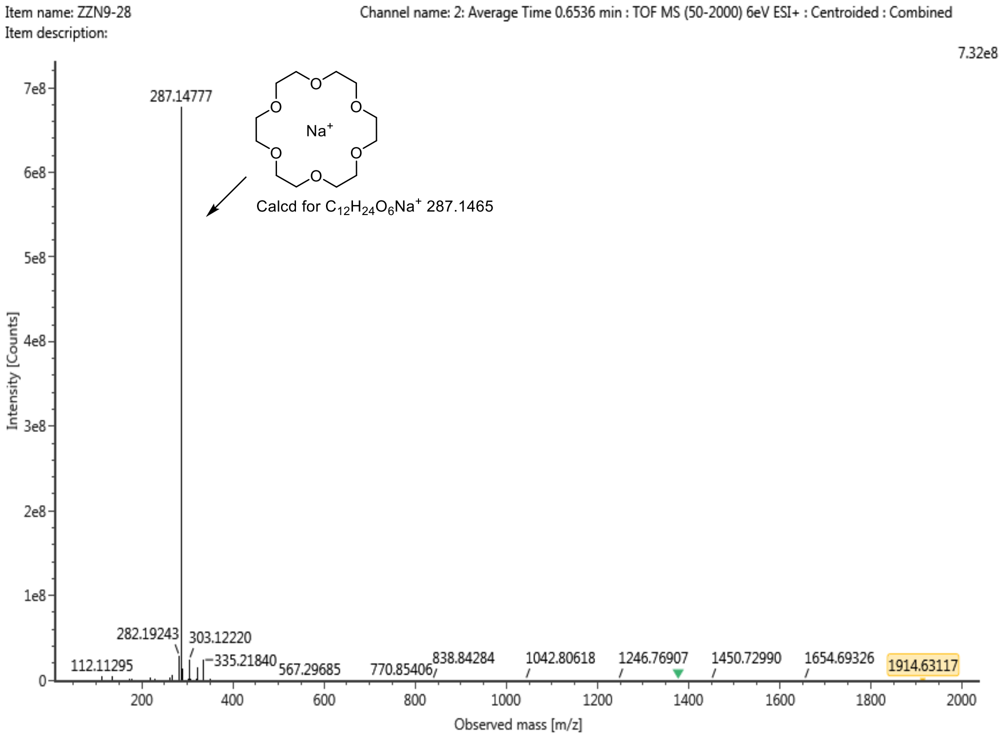

Supplementary Figure 43. The HRMS of 18-crown-6 and quinuclidinium ion.

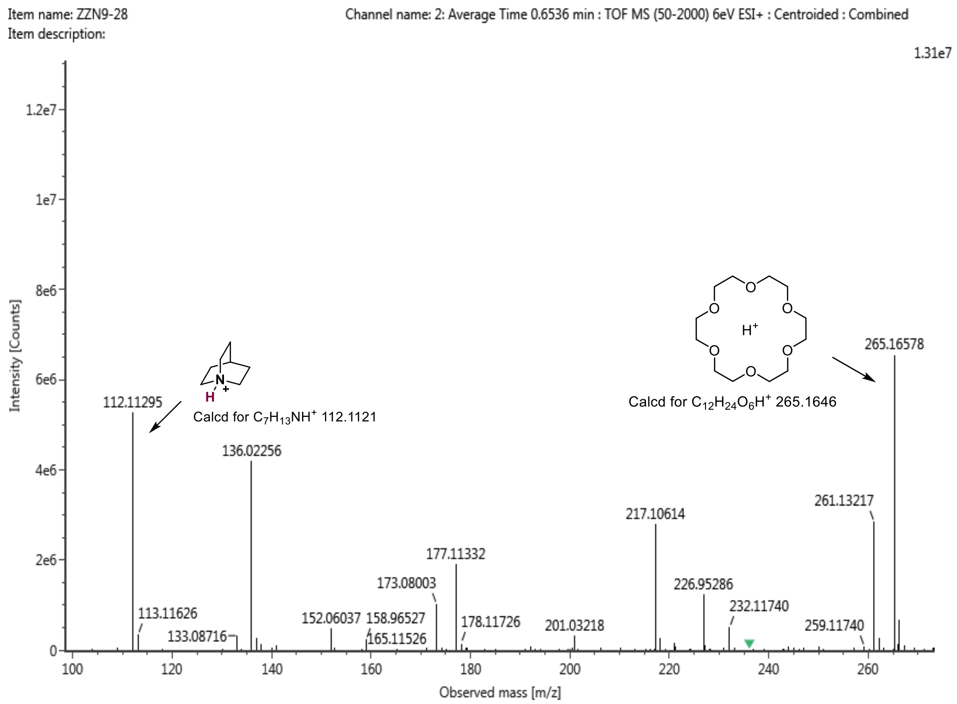

Supplementary Figure 44. The HRMS of 18-crown-6 and quinuclidinium ion.

Interestingly, as a parallel experiment, the HRMS of crown ether under the same condition observed only [18-crown-6 + Na^+] (calcd for $C_{12}H_{24}O_6Na^+$ 287.1465, found 287.1489) and [18-crown-6 + K^+] (calcd for $C_{12}H_{24}NK^+$ 303.1204, found 303.1225), while [18-crown-6 + H^+] is barely observed.

Although these experiments can not observe obvious interaction between quinuclidinium ion and crown ether, the interaction between crown ether and proton is indeed proven.

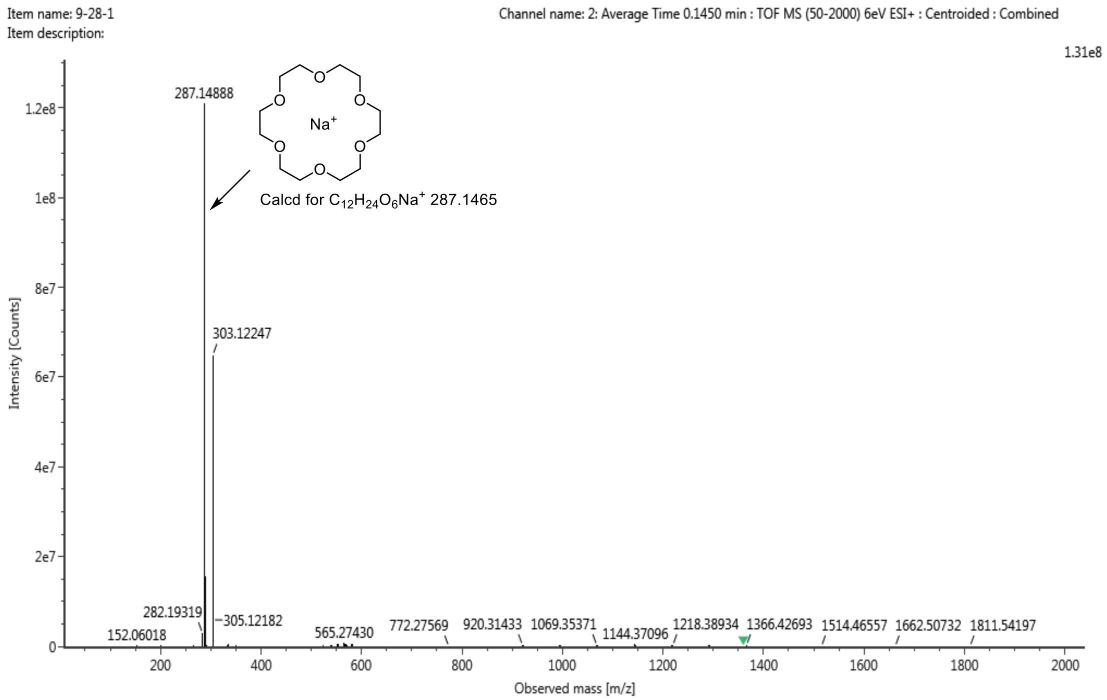

Supplementary Figure 45. The HRMS of 18-crown-6.

(10-9) Comparing the potential of Ir(II)/Ir(III) and Fe(II)/Fe(III)

Cyclic voltammograms were collected using 3-electrode cell consisting of a 3 mm glassy carbon working electrode, platinum wire as the counter electrode, and silver wire as the reference at ambient temperature in an N_2-filled atmosphere Sublimed ferrocene was added at the end of each experiment as the internal reference.

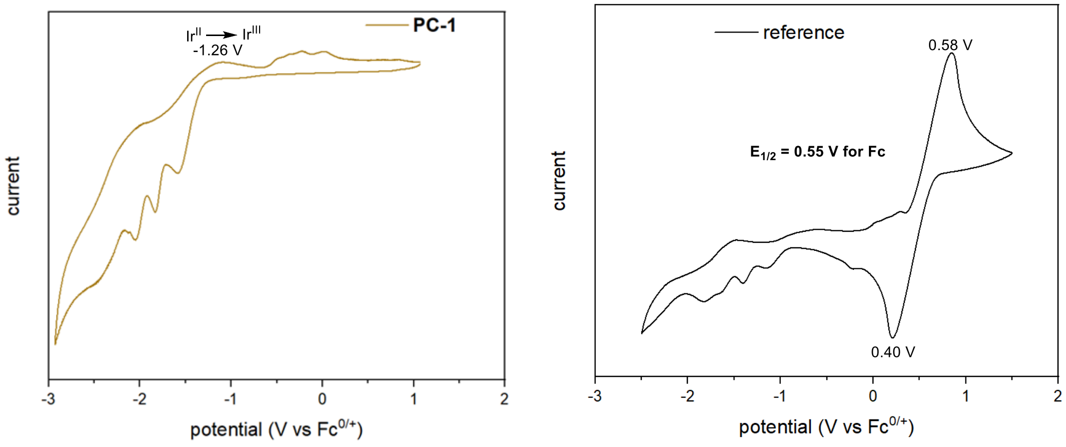

0.

Supplementary Figure 46 Cyclic voltammogram of **PC-1** in CH_3CN using 0.1 M $^nBu_4NPF_6$ as supporting electrolyte at ambient temperature; scan rate: 0.1 V/s, referenced to $Fc^{0/+}$.

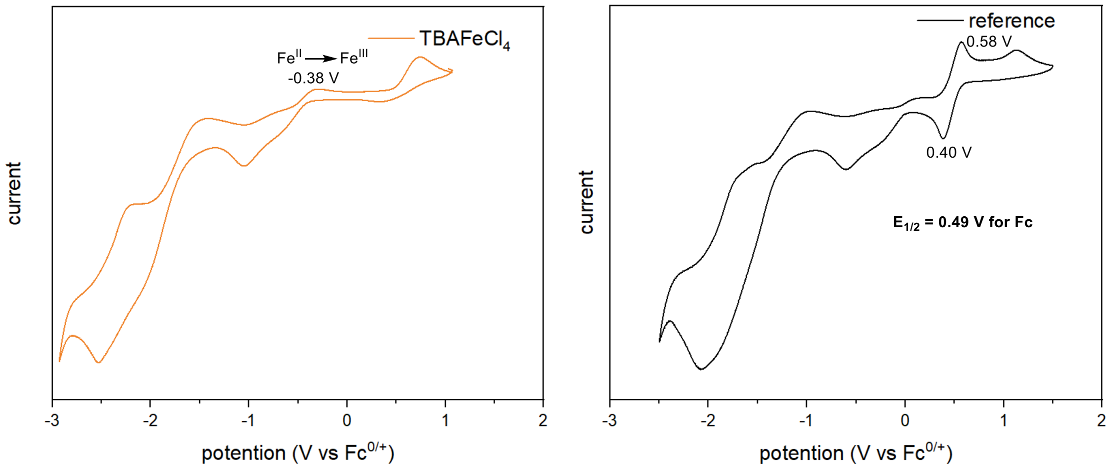

Supplementary Figure 47 Cyclic voltammogram of $TBAFeCl_4$ in CH_3CN using 0.1 M $^{n}Bu_4NPF_6$ as supporting electrolyte at ambient temperature; scan rate: 0.1 V/s, referenced to $Fc^{0/+}$.

(11) Computational details

All density functional theory (DFT) calculations were performed using the Gaussian 09 software package[4]. The geometry optimization was carried out using the B3LYP[5-6] functional with Grimme's dispersion correction methods of D3BJ versions[7]. A mixed basis set of SDD[8-9] (Ir, Fe) and 6-31G(d)[10-12] (C, H, O, N, P, Br, Cl) was utilized in the gas phase. Vibrational frequencies were calculated for all optimized structures at the same level of theory to confirm that they are either local minima (no imaginary frequency) or transition states (only one imaginary frequency) and to obtain the thermal correction to free energies at 298.15 K and 1 atm pressure. To obtain more accurate energies, solvation single-point energy calculations were performed at a higher level of theory using M06[13]/6-311+G(d,p)[14-15]-SDD in acetonitrile solvent with SMD continuum solvation model[16].

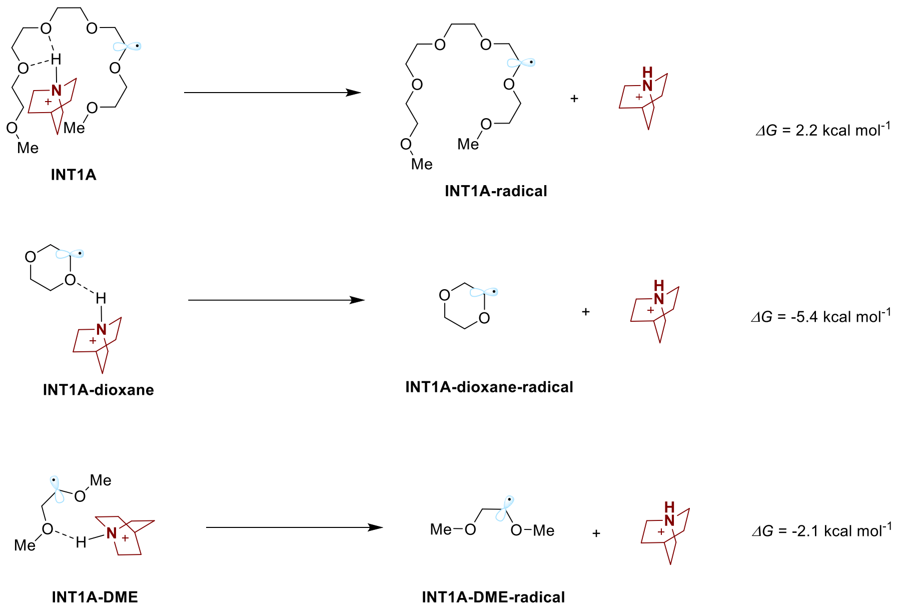

Supplementary Figure 48 The dissociation energy of **INT1A**.

(12) Spectra

^{1}H NMR (400 MHz, $CDCl_3$) of **PEG 2000-1**

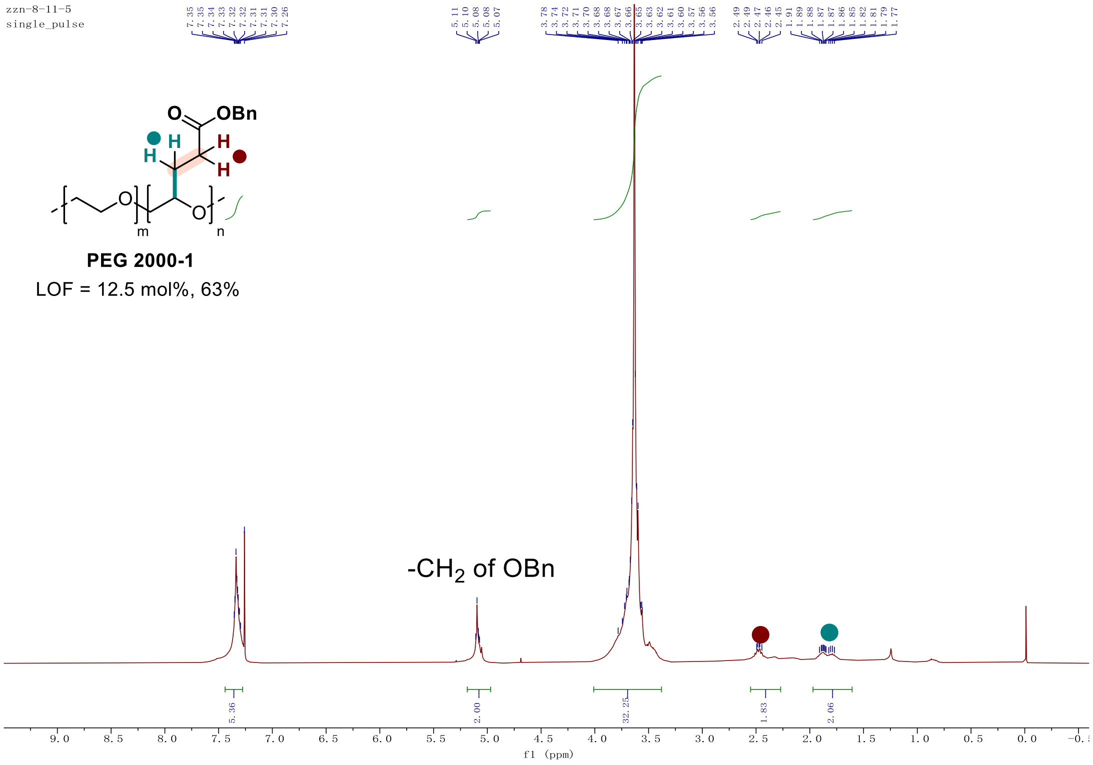

^{13}C NMR (151 MHz, $CDCl_3$) of **PEG 2000-1**

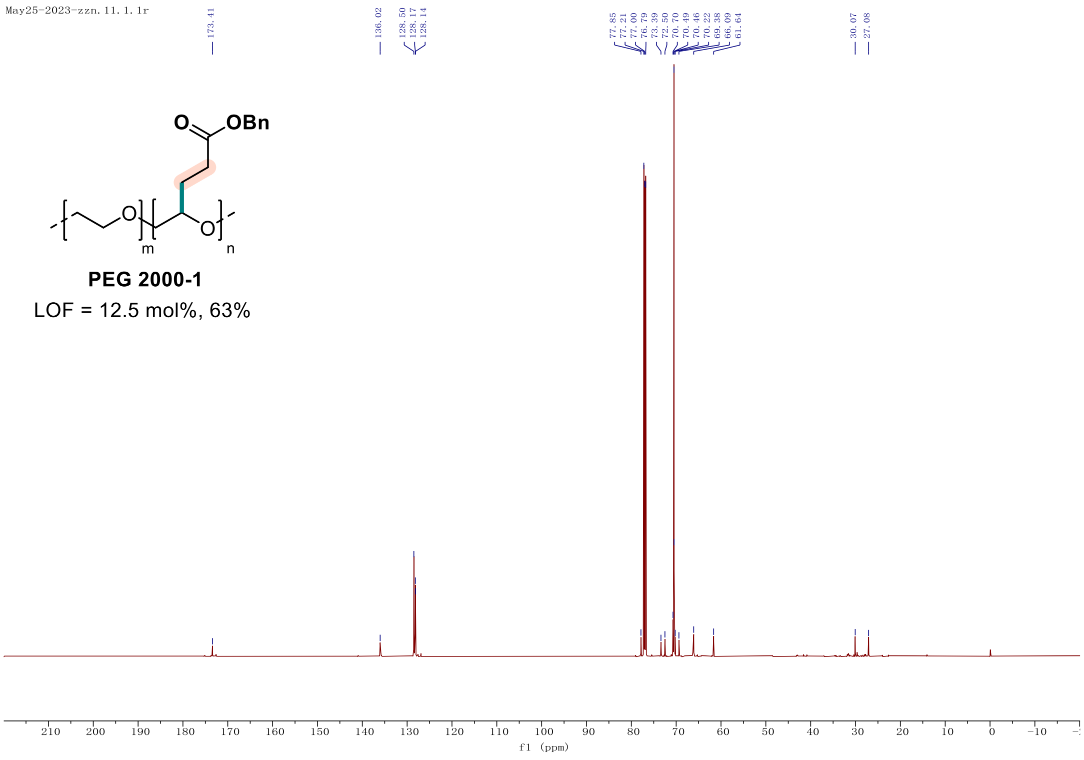

COSY of **PEG 2000-1**

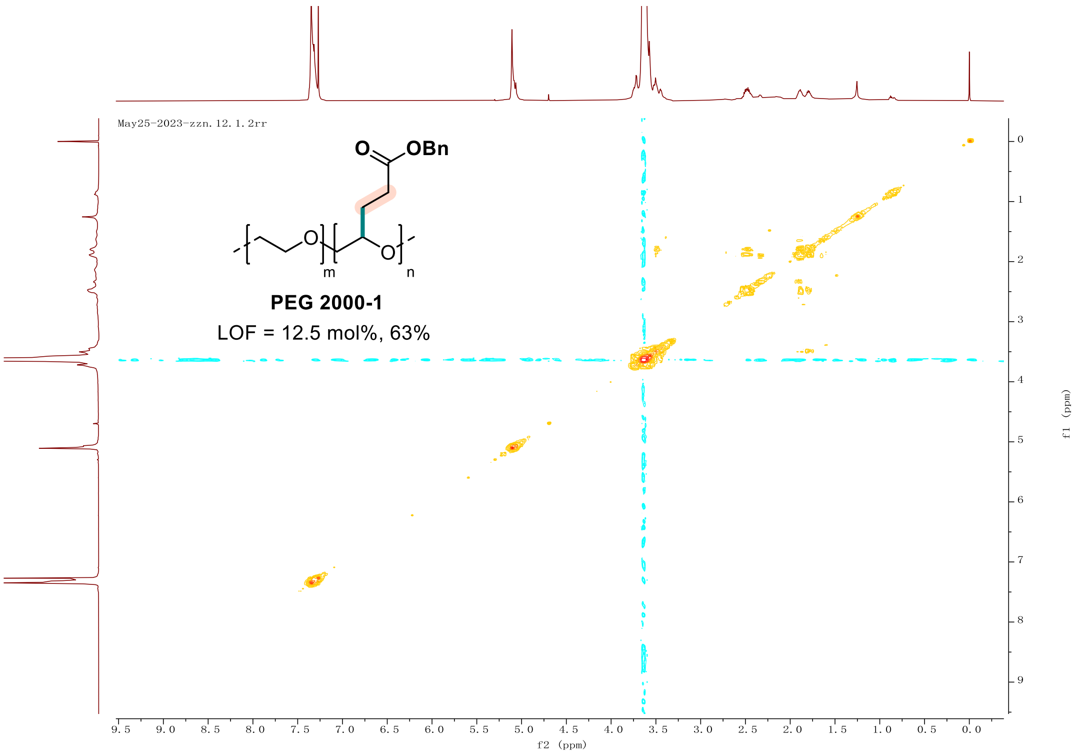

HMBC of **PEG 2000-1**

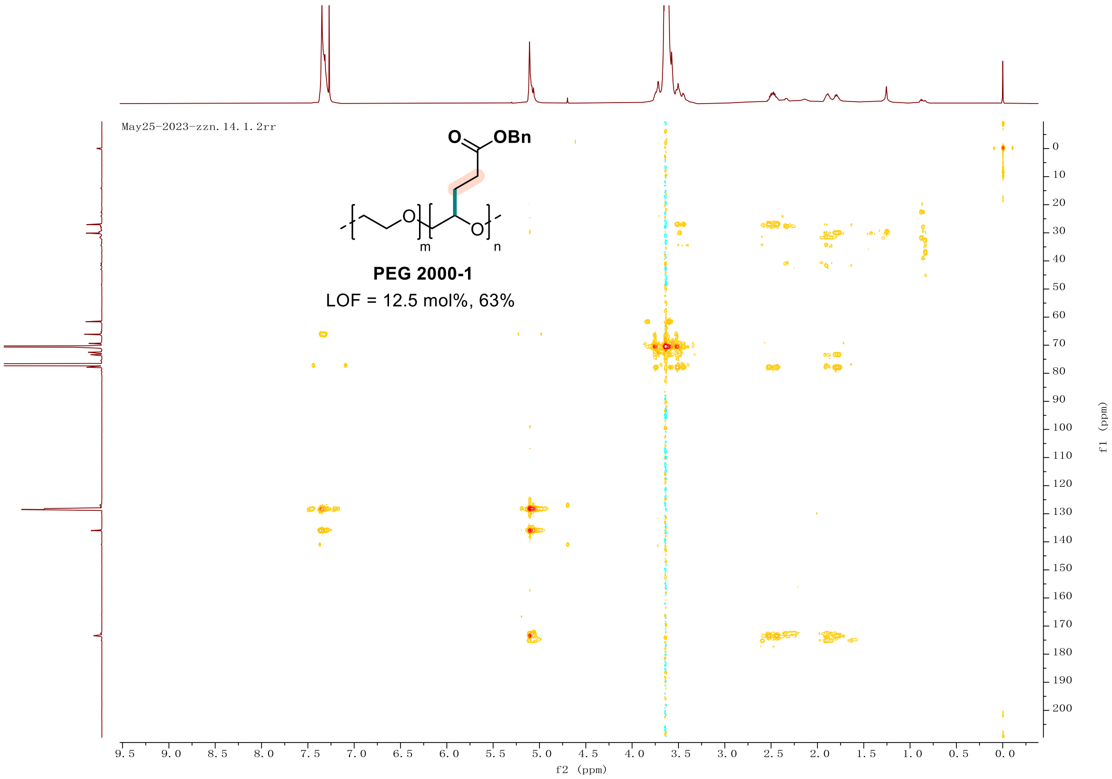

DOSY of **PEG 2000-1**

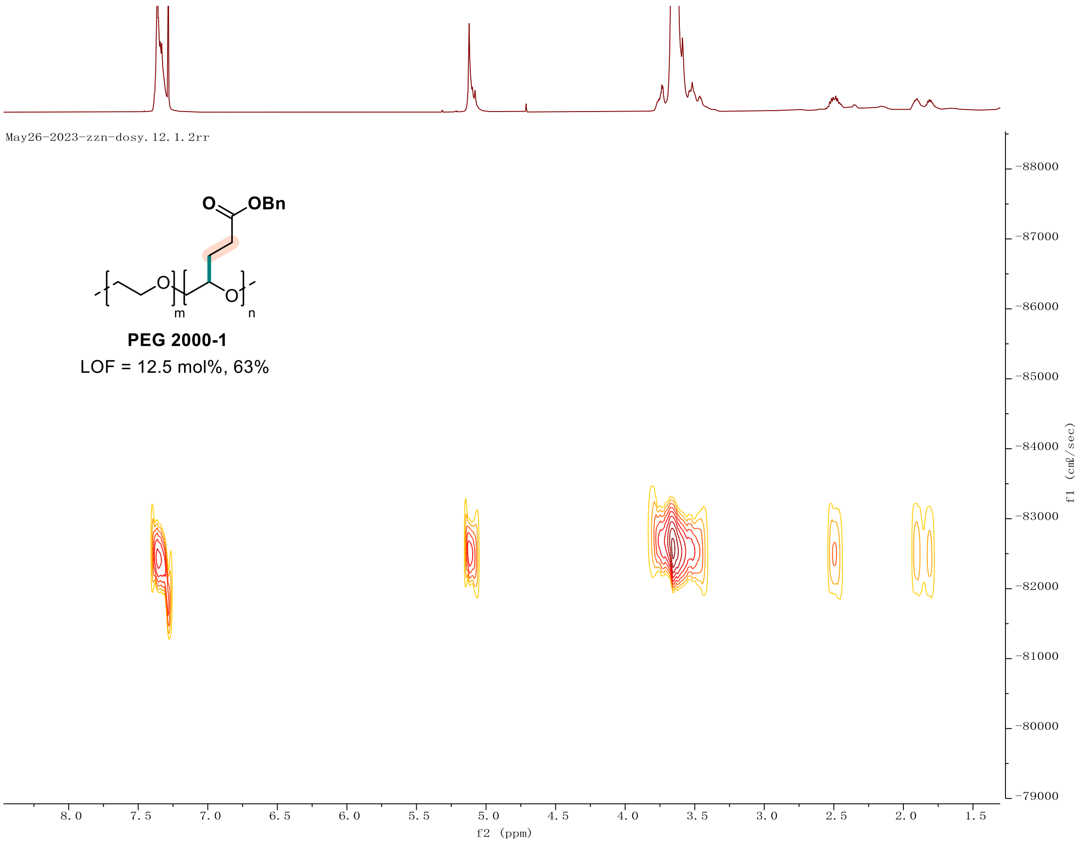

^{1}H NMR (400 MHz, $CDCl_3$) of **PEG 2000-2**

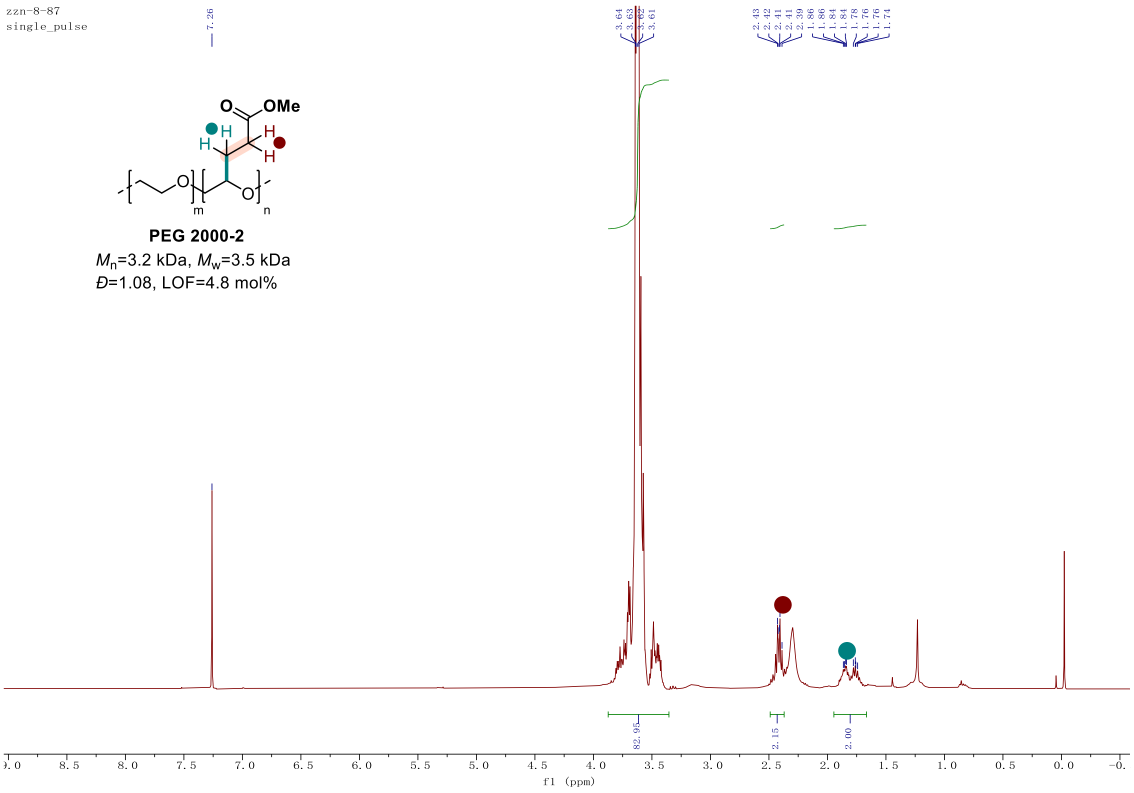

^{13}C NMR (151 MHz, $CDCl_3$) of **PEG 2000-2**

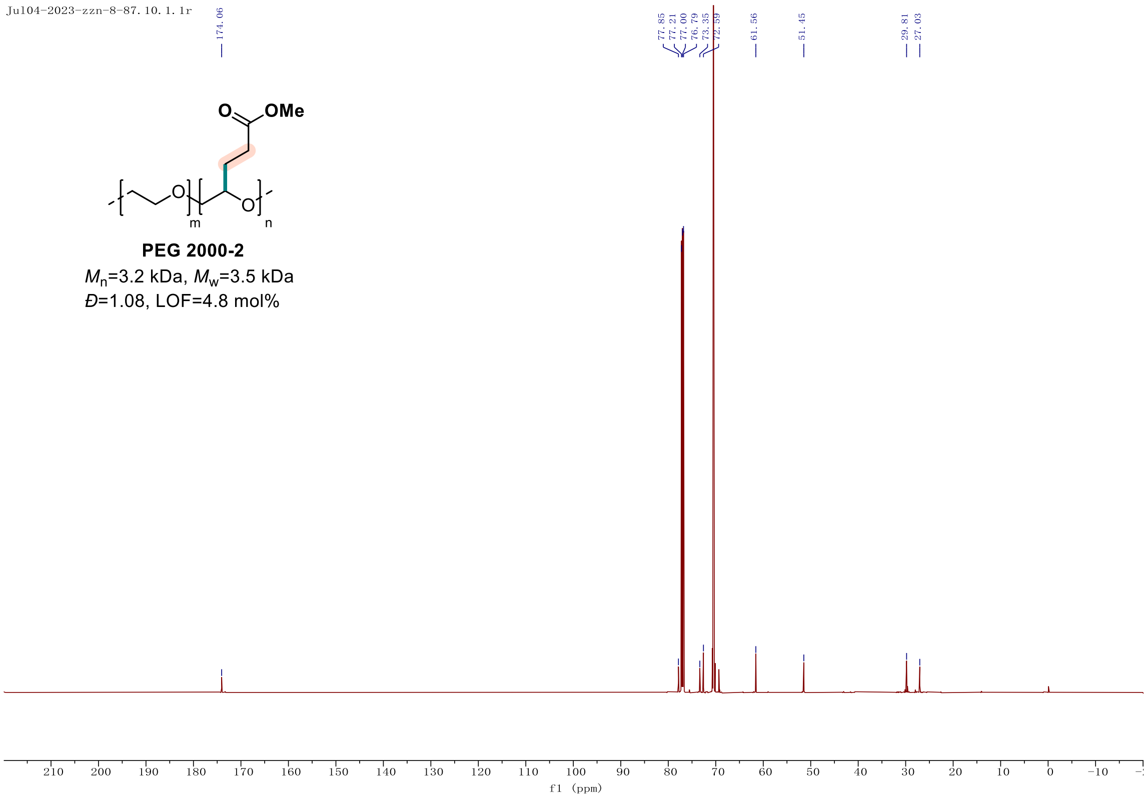

^{1}H NMR (400 MHz, $CDCl_3$) of **PEG 2000-3**

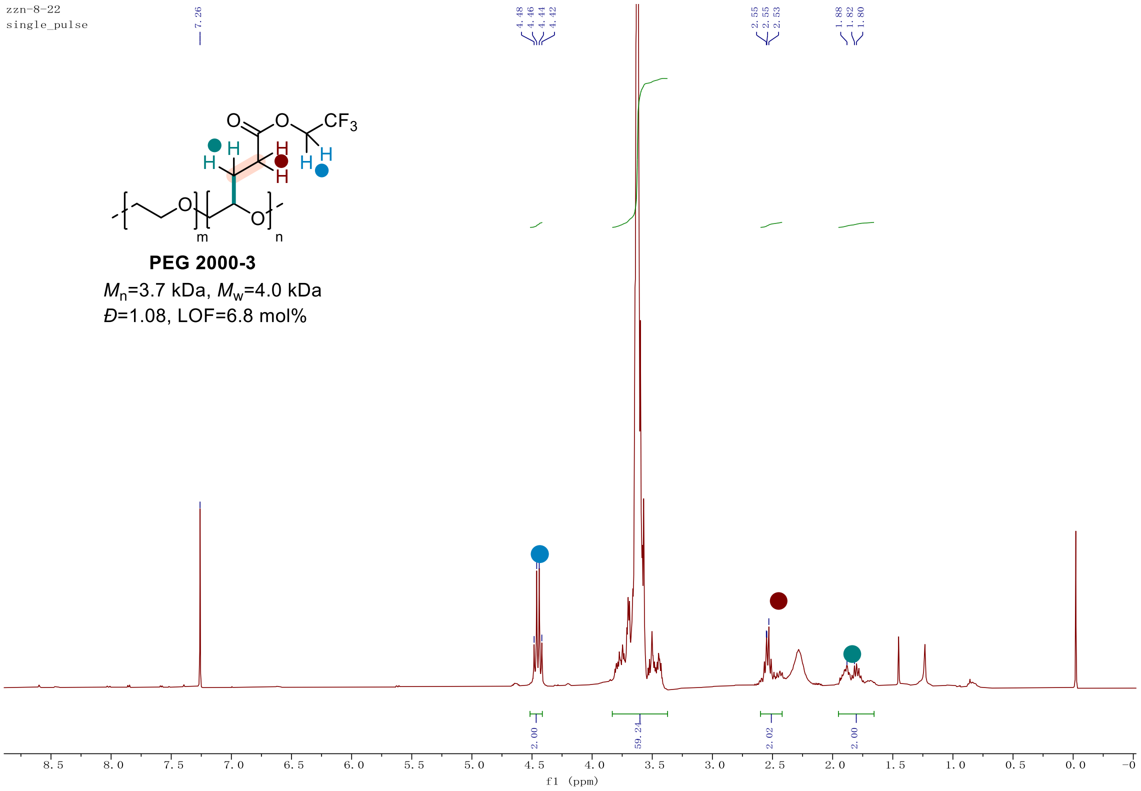

^{13}C NMR (151 MHz, $CDCl_3$) of **PEG 2000-3**

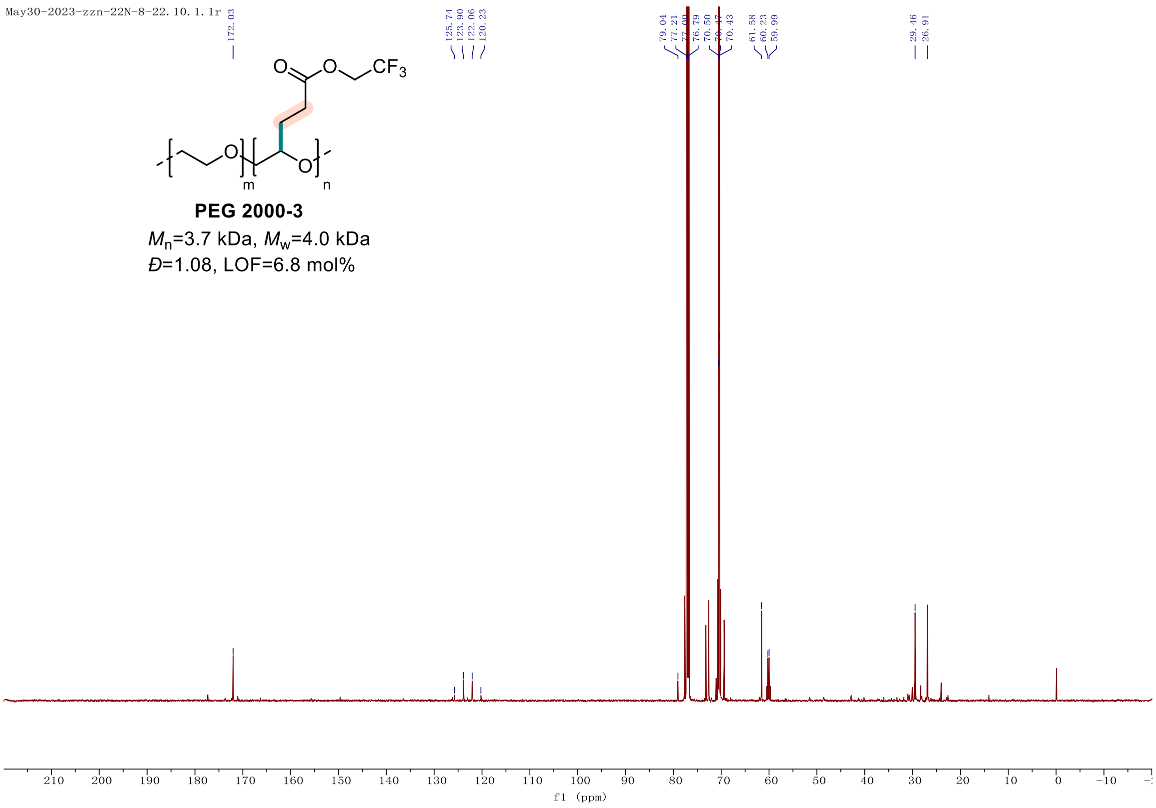

^{19}F NMR (376 MHz, $CDCl_3$) of **PEG 2000-3**

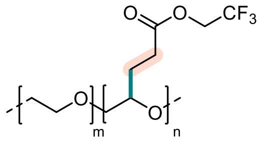

PEG 2000-3

M_n=3.7 kDa, M_w=4.0 kDa

$Đ$=1.08, LOF=6.8 mol%

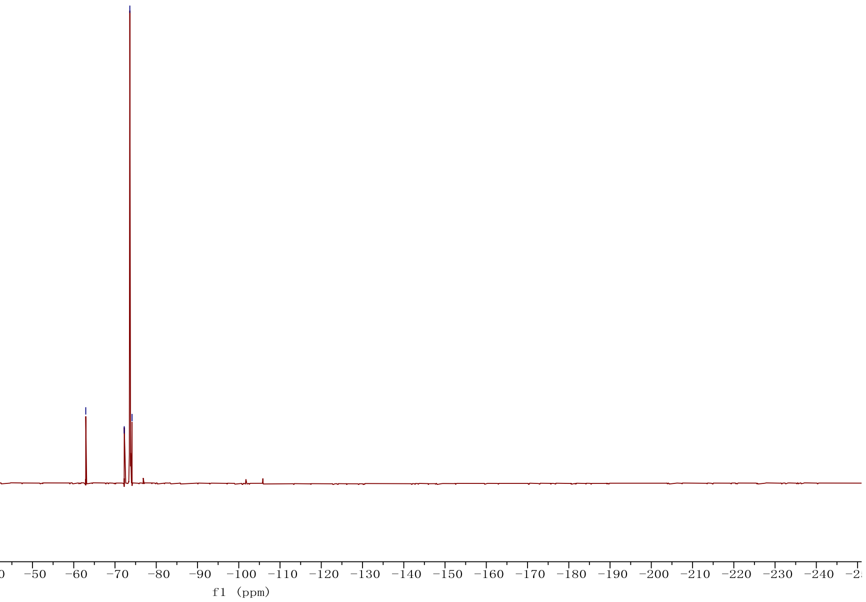

^{1}H NMR (400 MHz, $CDCl_3$) of **PEG 2000-4**

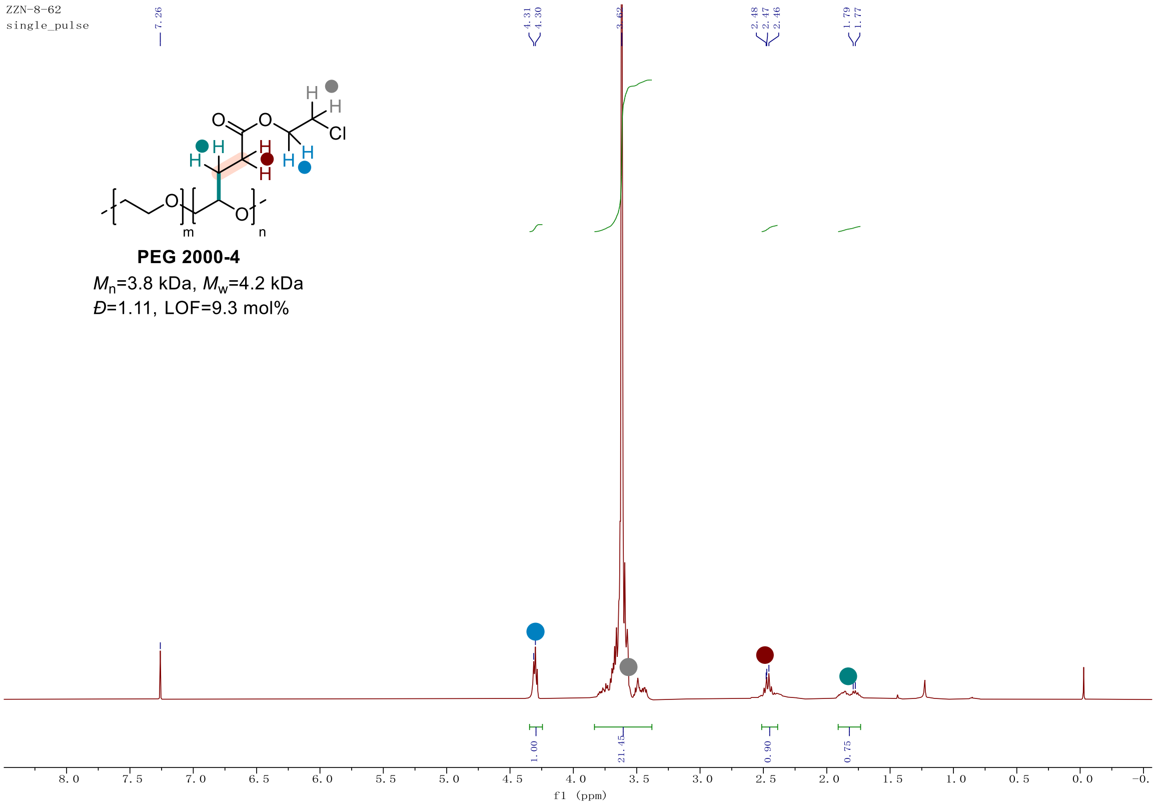

^{13}C NMR (151 MHz, $CDCl_3$) of **PEG 2000-4**

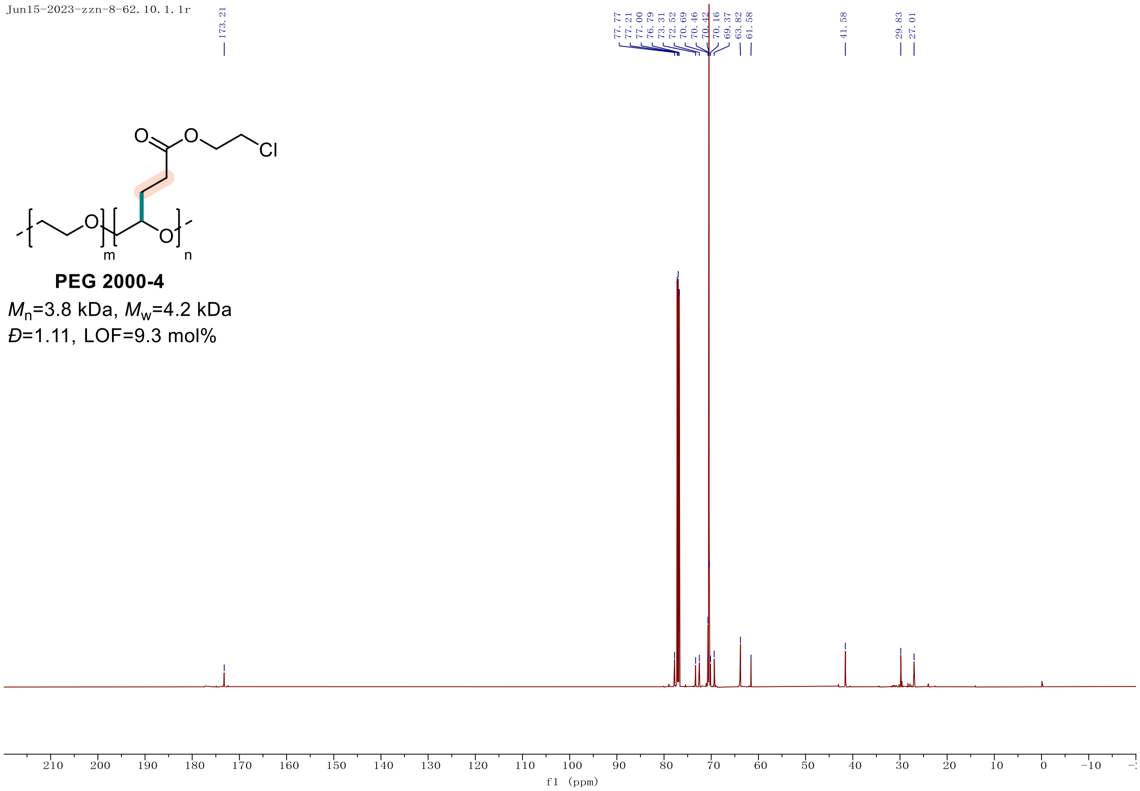

^{1}H NMR (400 MHz, $CDCl_3$) of **PEG 2000-5**

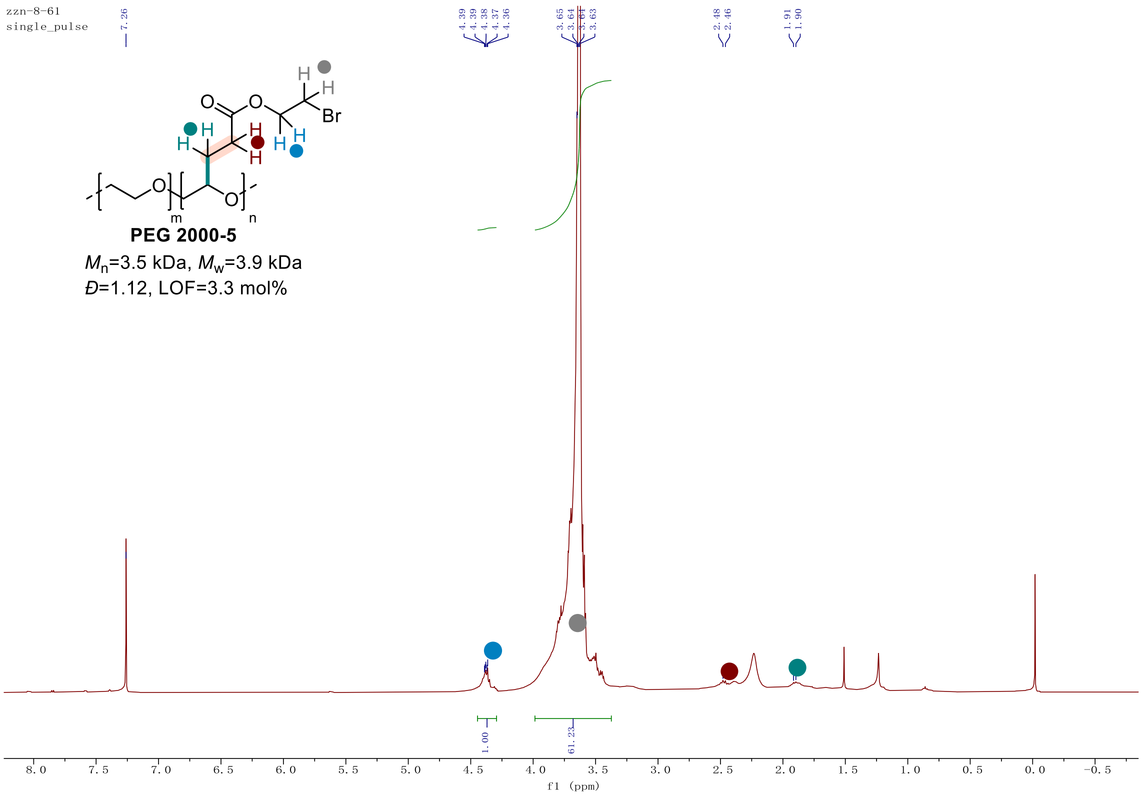

The ^{13}C NMR (151 MHz, $CDCl_3$) of **PEG 2000-5**

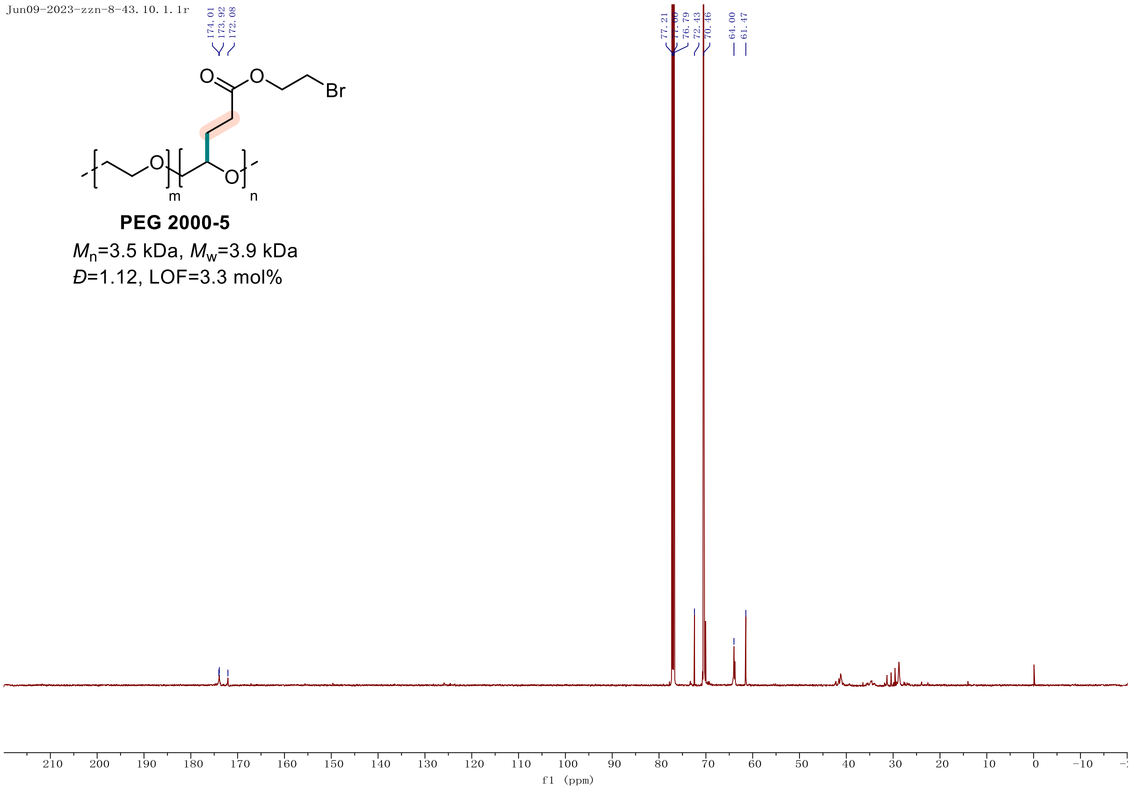

^{1}H NMR (400 MHz, $CDCl_3$) of **PEG 2000-6**

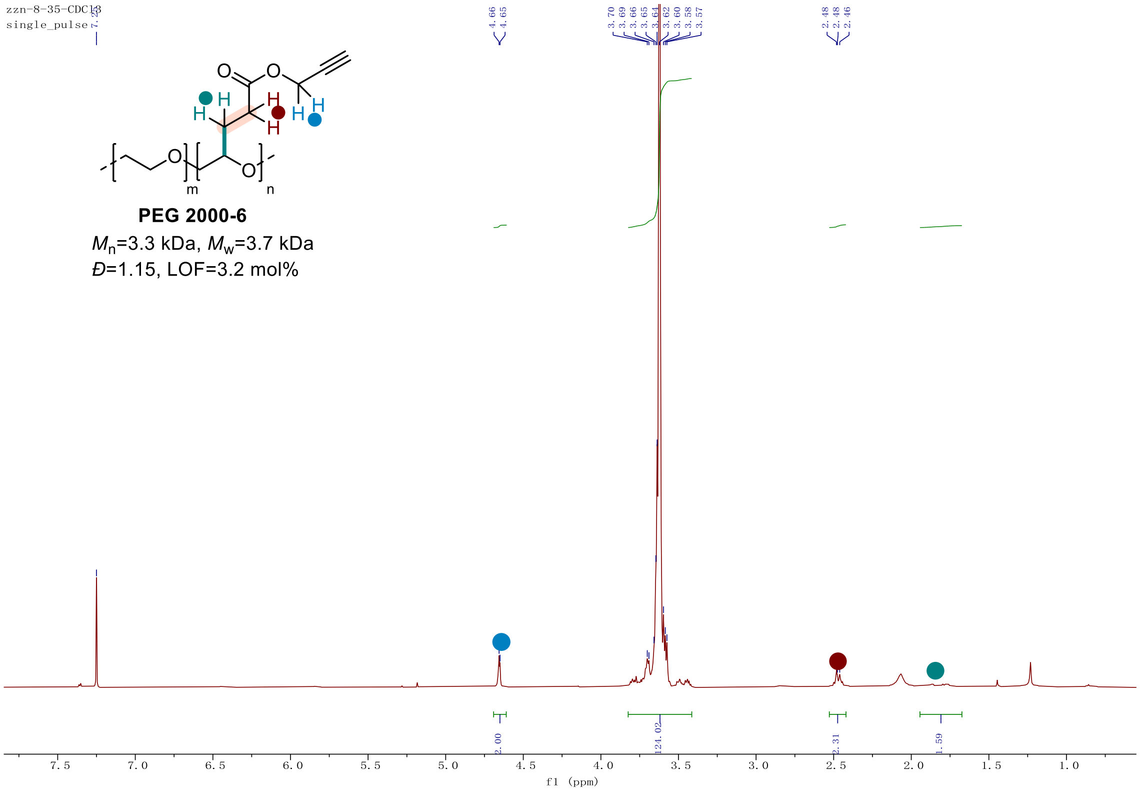

^{13}C NMR (151 MHz, $CDCl_3$) of **PEG 2000-6**

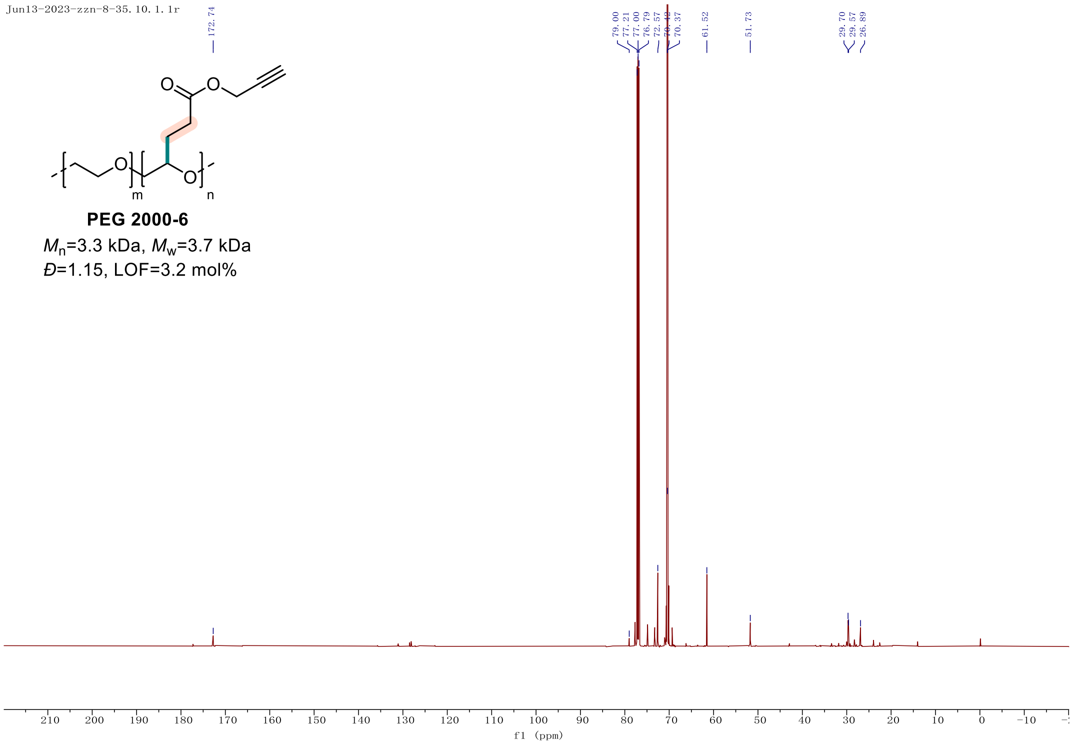

^{1}H NMR (400 MHz, $CDCl_3$) of **PEG 2000-7**

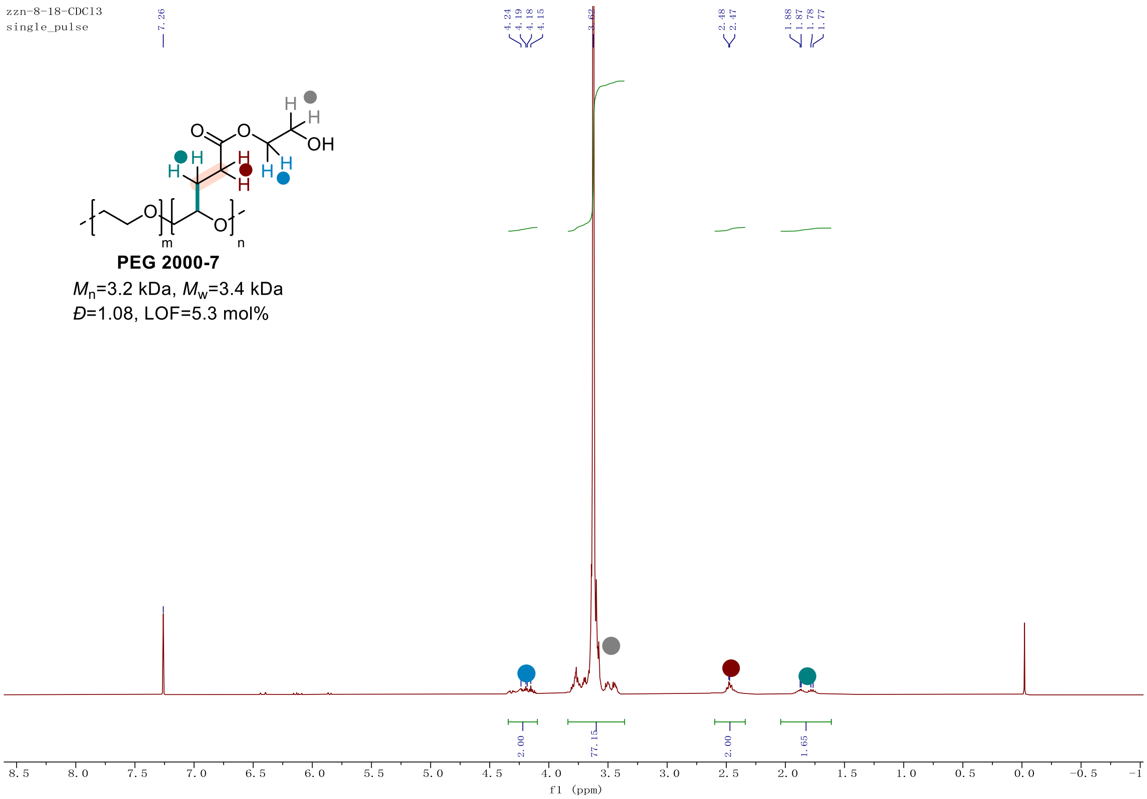

^{13}C NMR (151 MHz, $CDCl_3$) of **PEG 2000-7**

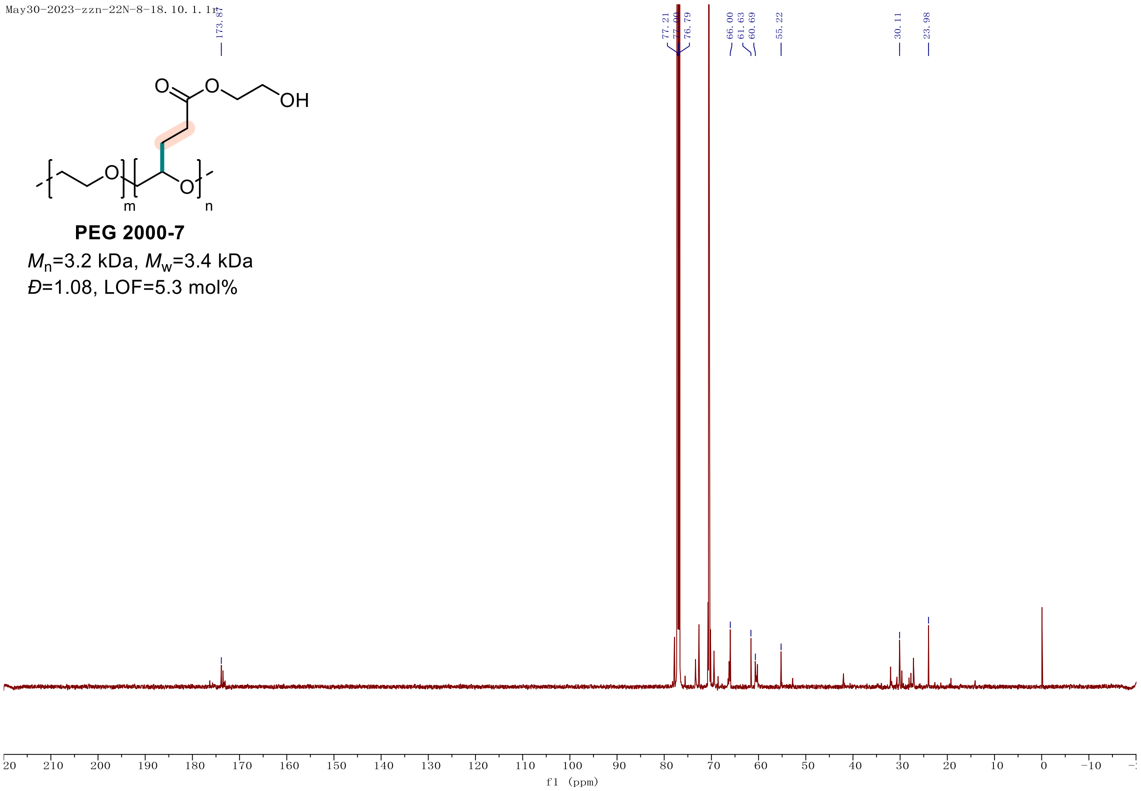

^{1}H NMR (400 MHz, $CDCl_3$) of **PEG 2000-8**

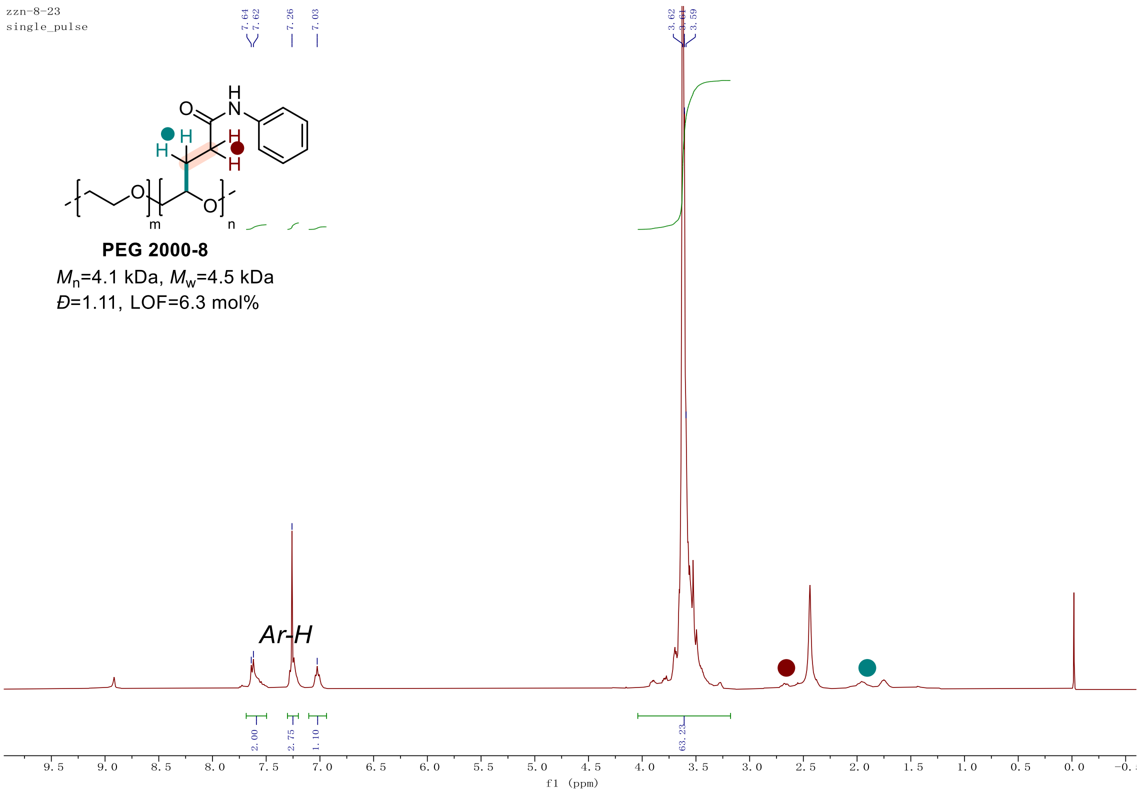

^{13}C NMR (151 MHz, $CDCl_3$) of **PEG 2000-8**

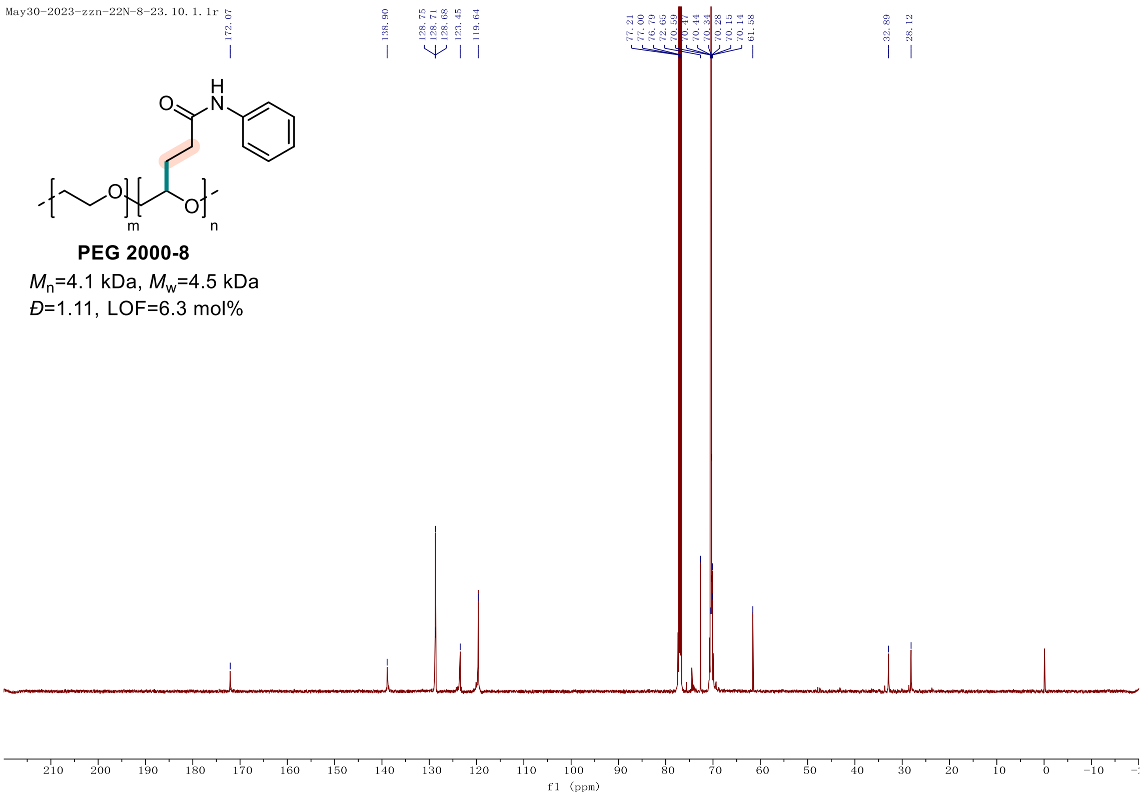

^{1}H NMR (400 MHz, $CDCl_3$) of **PEG 2000-9**

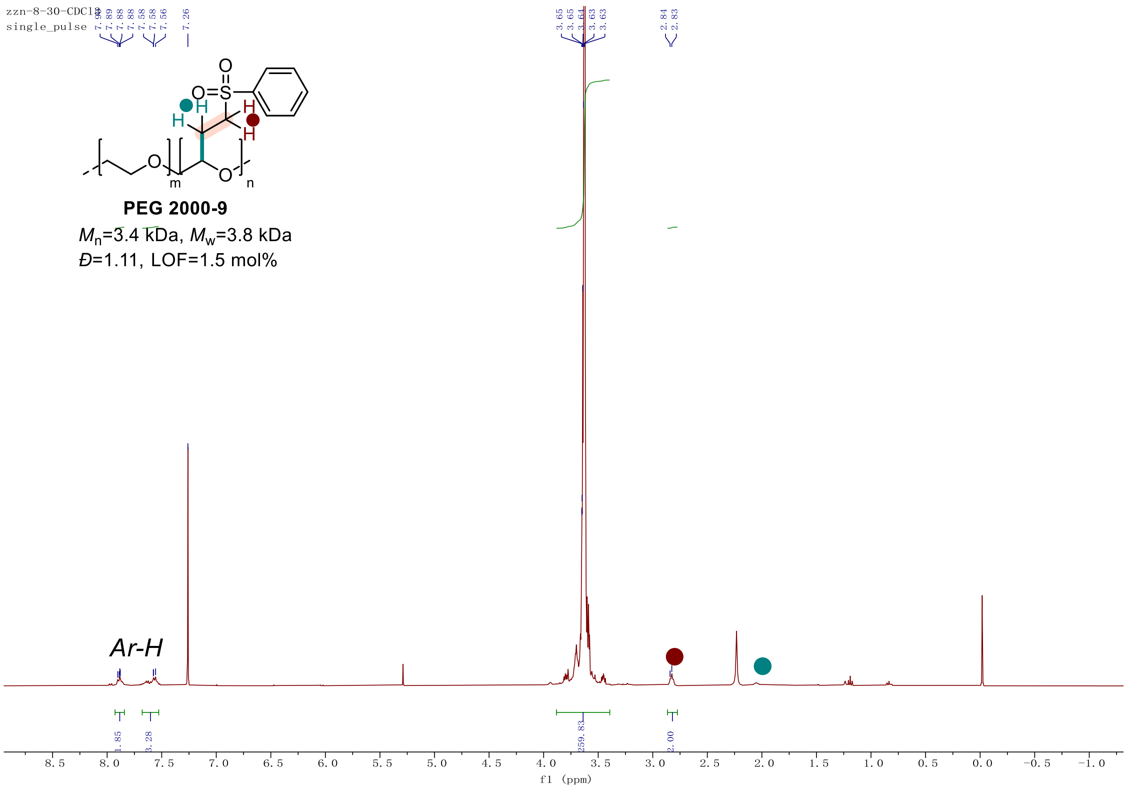

^{1}H NMR (400 MHz, $CDCl_3$) of **PEG 2000-9**

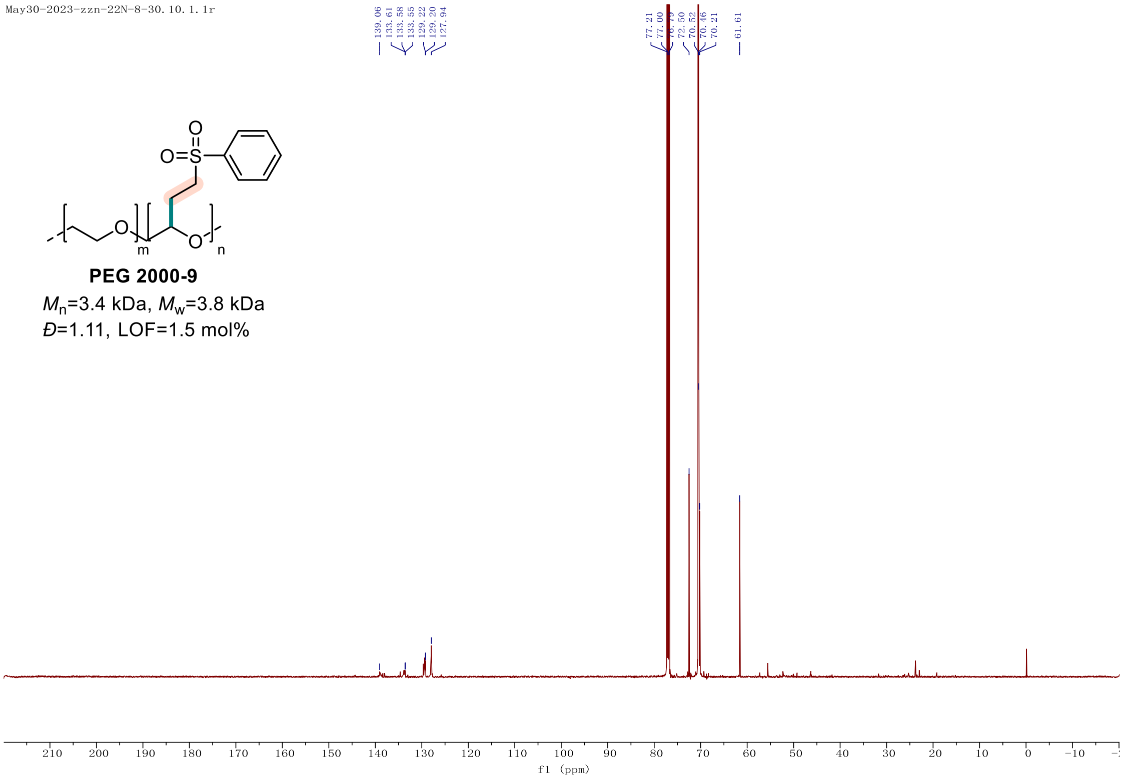

^{1}H NMR (400 MHz, $CDCl_3$) of **PEG 2000-10**

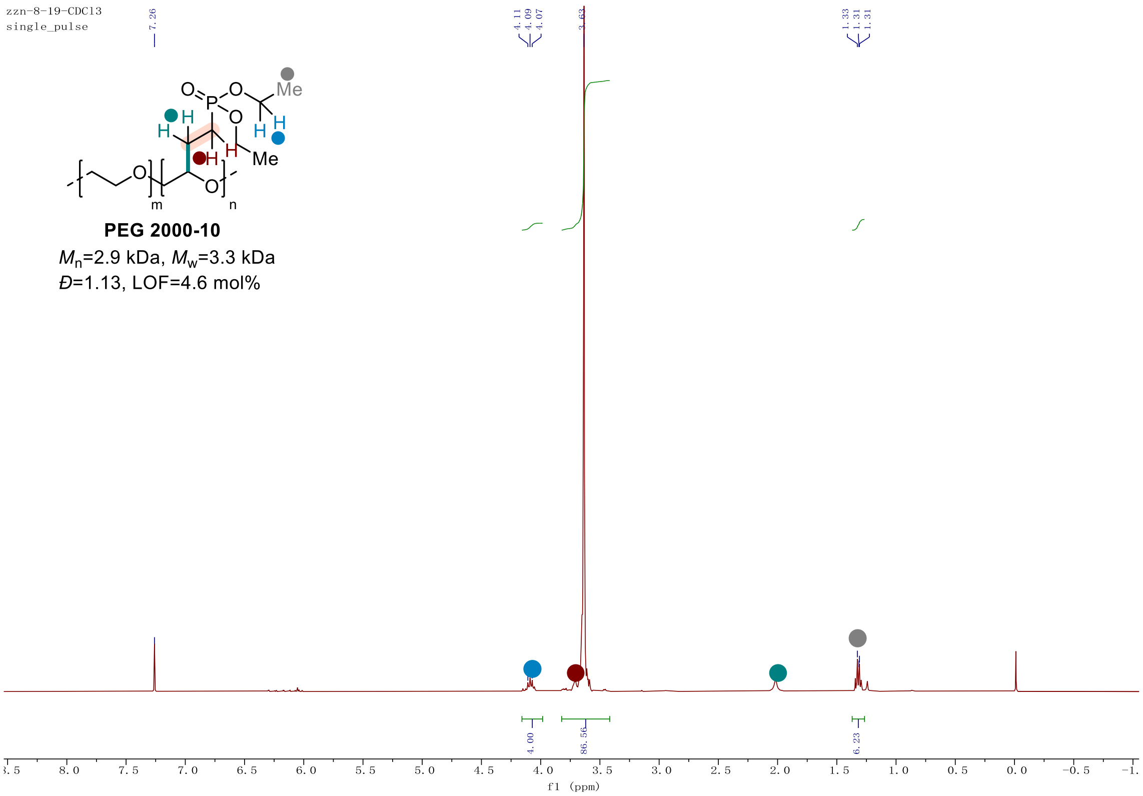

^{13}C NMR (151 MHz, $CDCl_3$) of **PEG 2000-10**

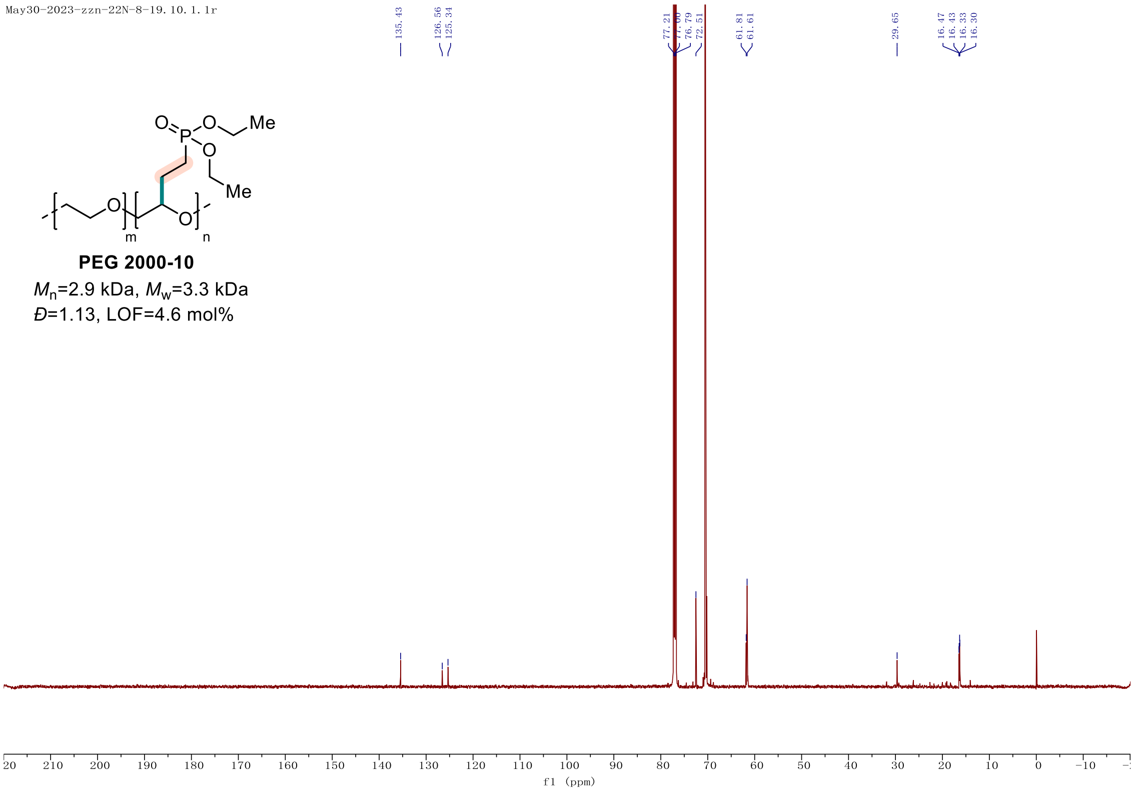

^{31}P NMR (162 MHz, $CDCl_3$) of **PEG 2000-10**

zzn-7-117-CDCl3
single pulse decoupled gated NOE

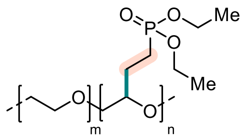

PEG 2000-10
M_n=2.9 kDa, M_w=3.3 kDa
$Đ$=1.13, LOF=4.6 mol%

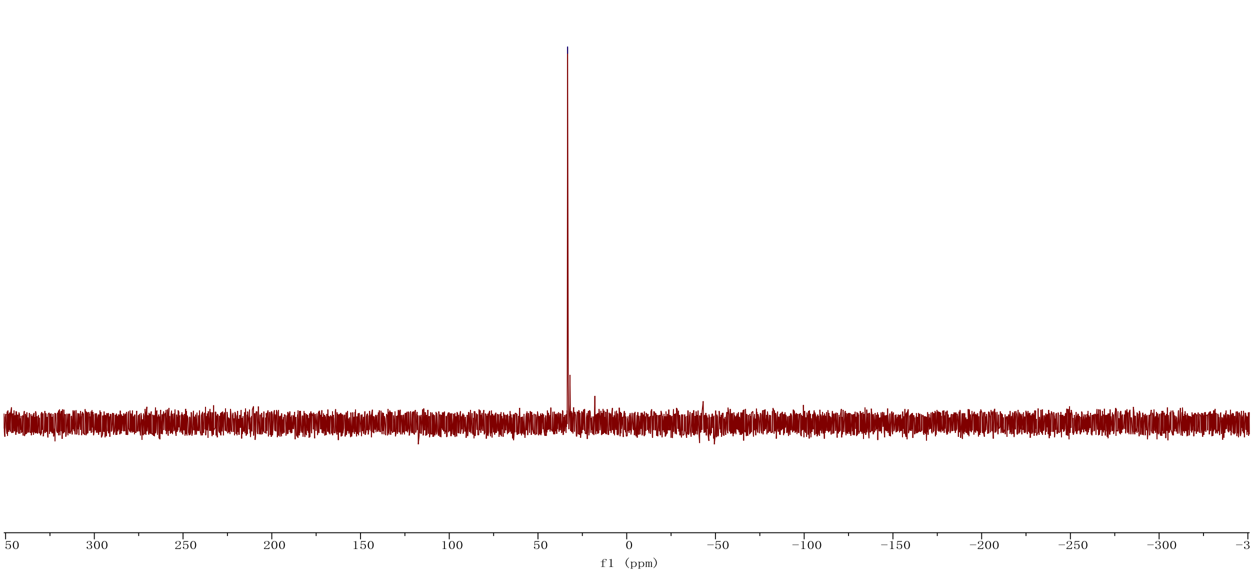

^{1}H NMR (400 MHz, $CDCl_3$) of **PEG 2000-11**

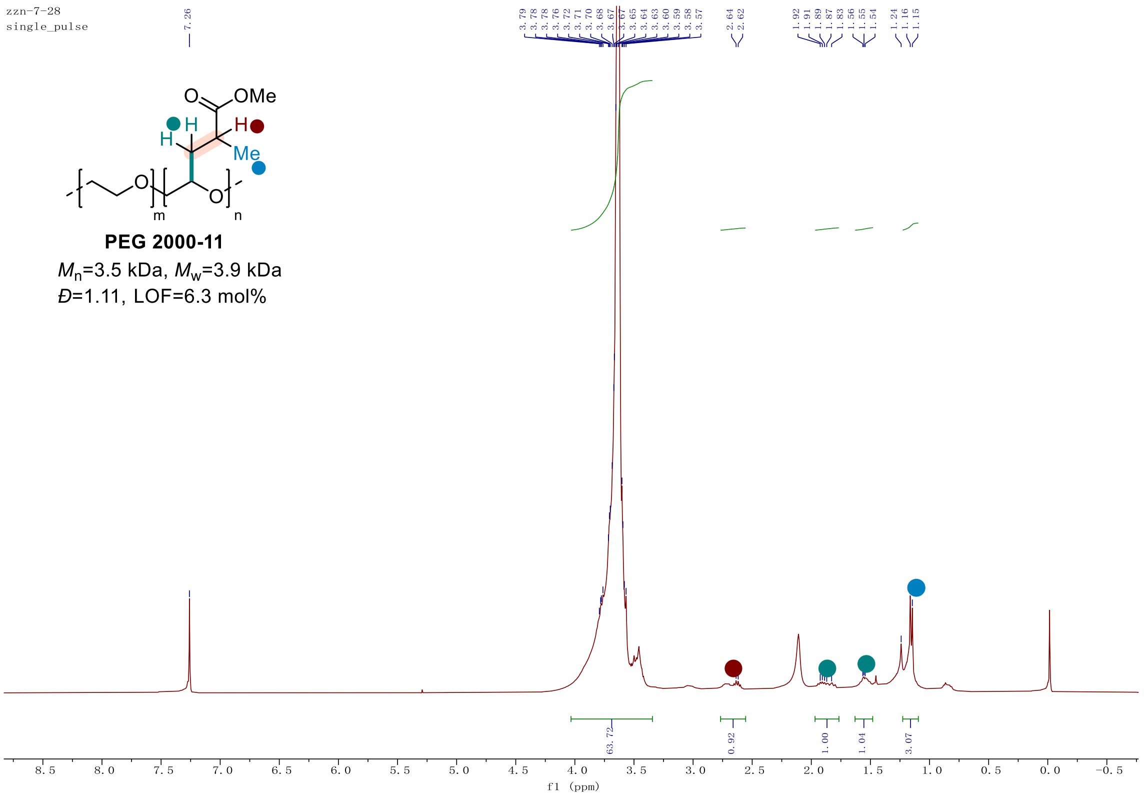

^{13}C NMR (151 MHz, $CDCl_3$) of **PEG 2000-11**

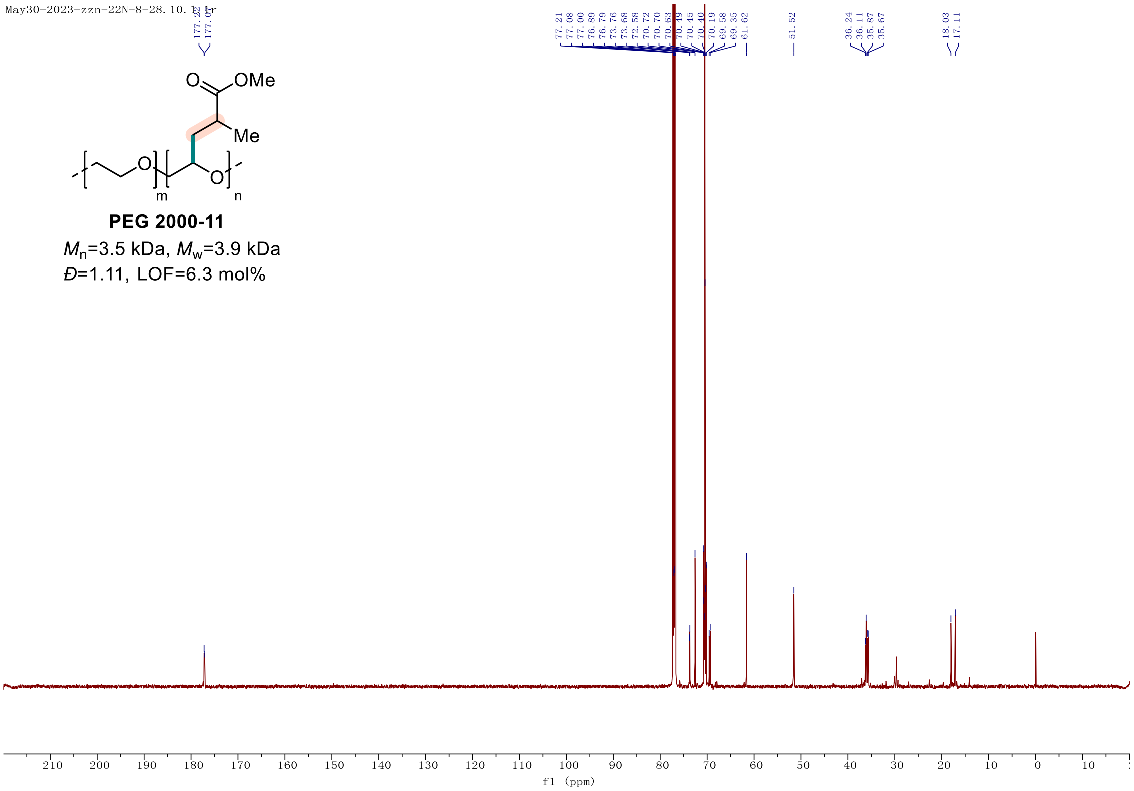

COSY of **PEG 2000-11**

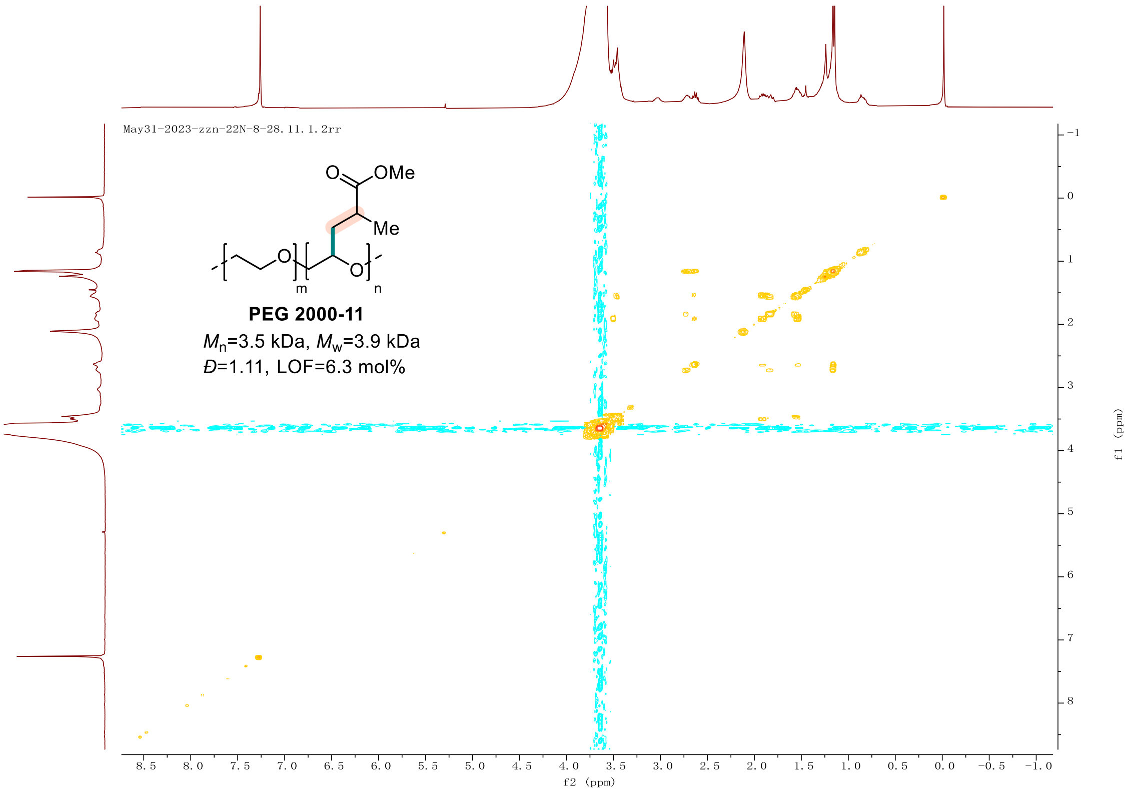

HMBC of **PEG 2000-11**

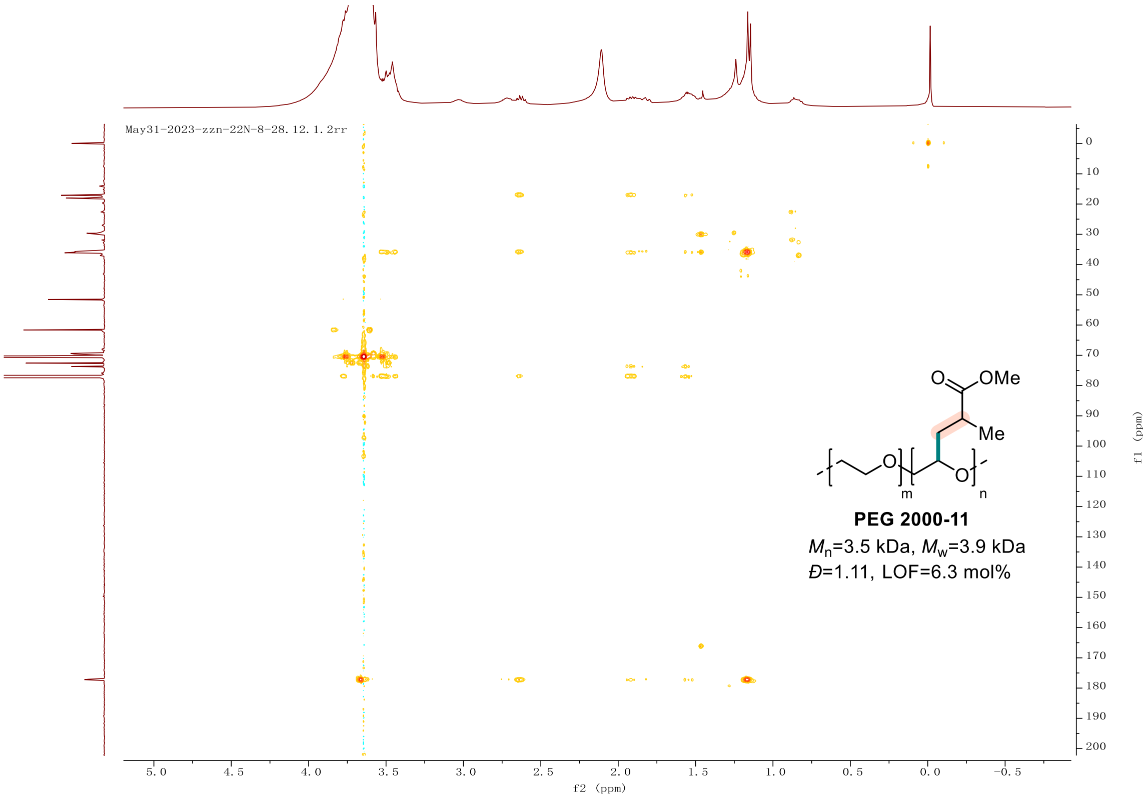

DOSY of **PEG 2000-11**

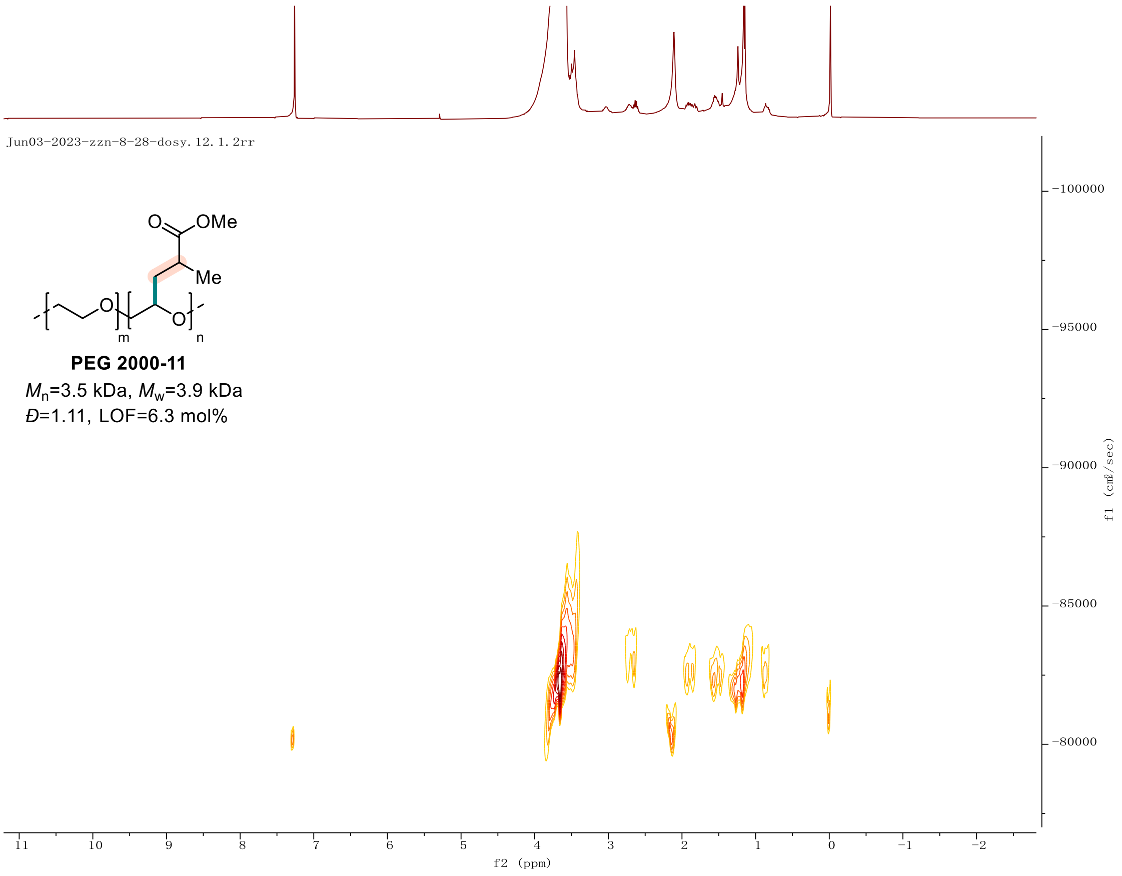

^{1}H NMR (400 MHz, $CDCl_3$) of **PEG 2000-12**

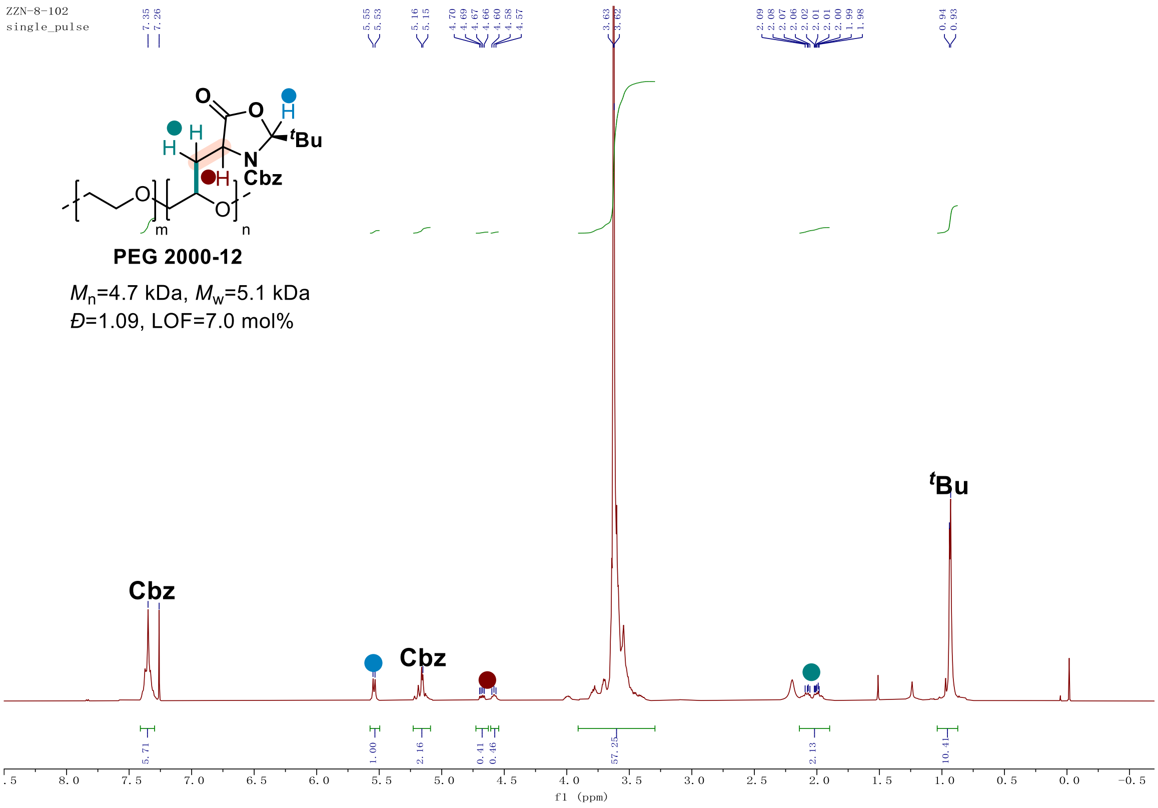

^{13}C NMR (151 MHz, $CDCl_3$) of **PEG 2000-12**

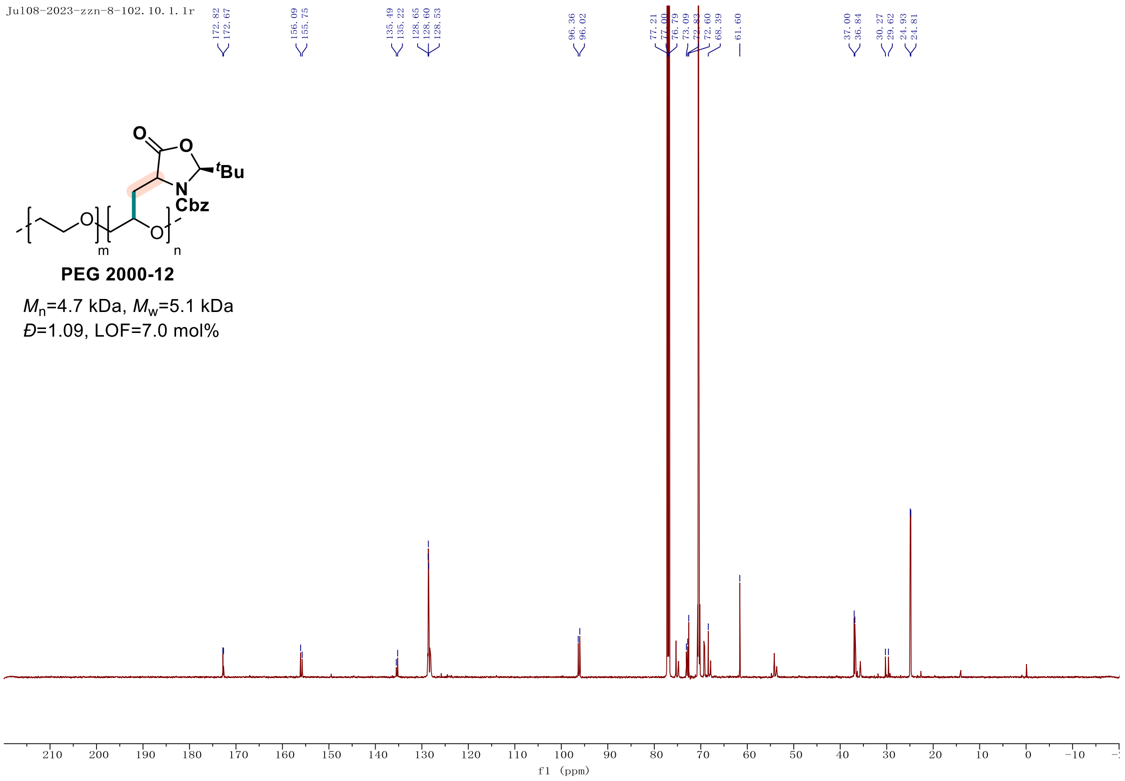

^{1}H NMR (400 MHz, $CDCl_3$) of **PEG 12000-13**

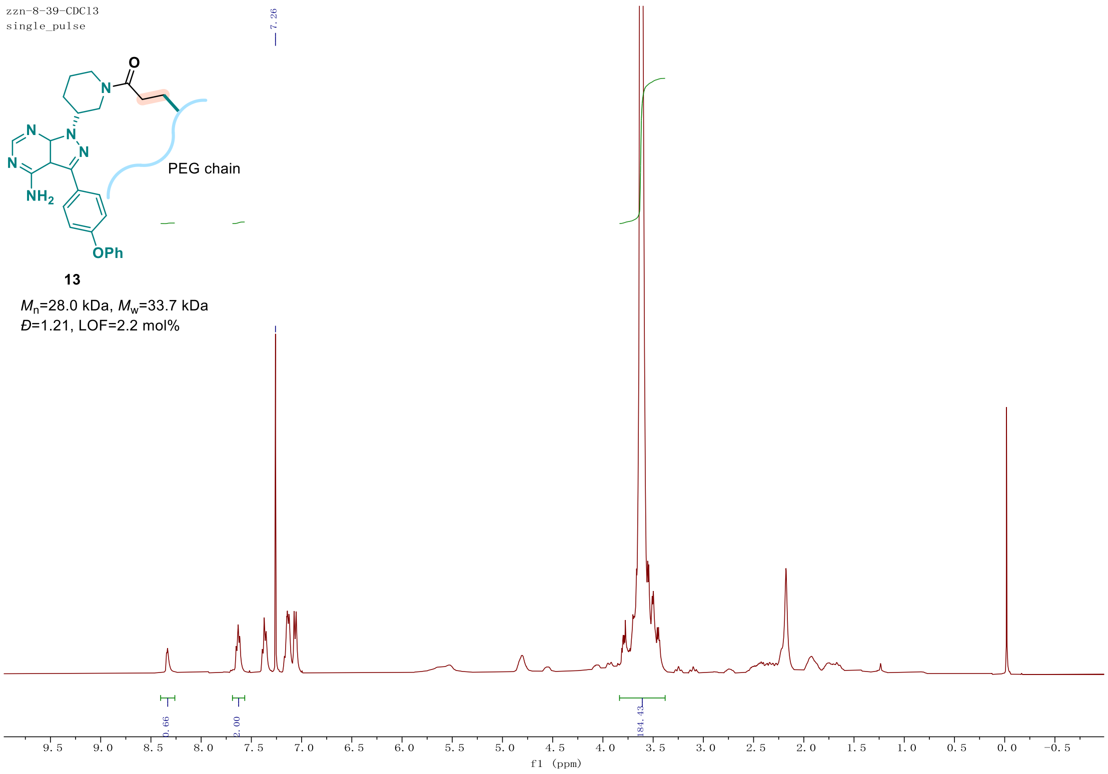

^{13}C NMR (151 MHz, $CDCl_3$) of **PEG 12000-13**

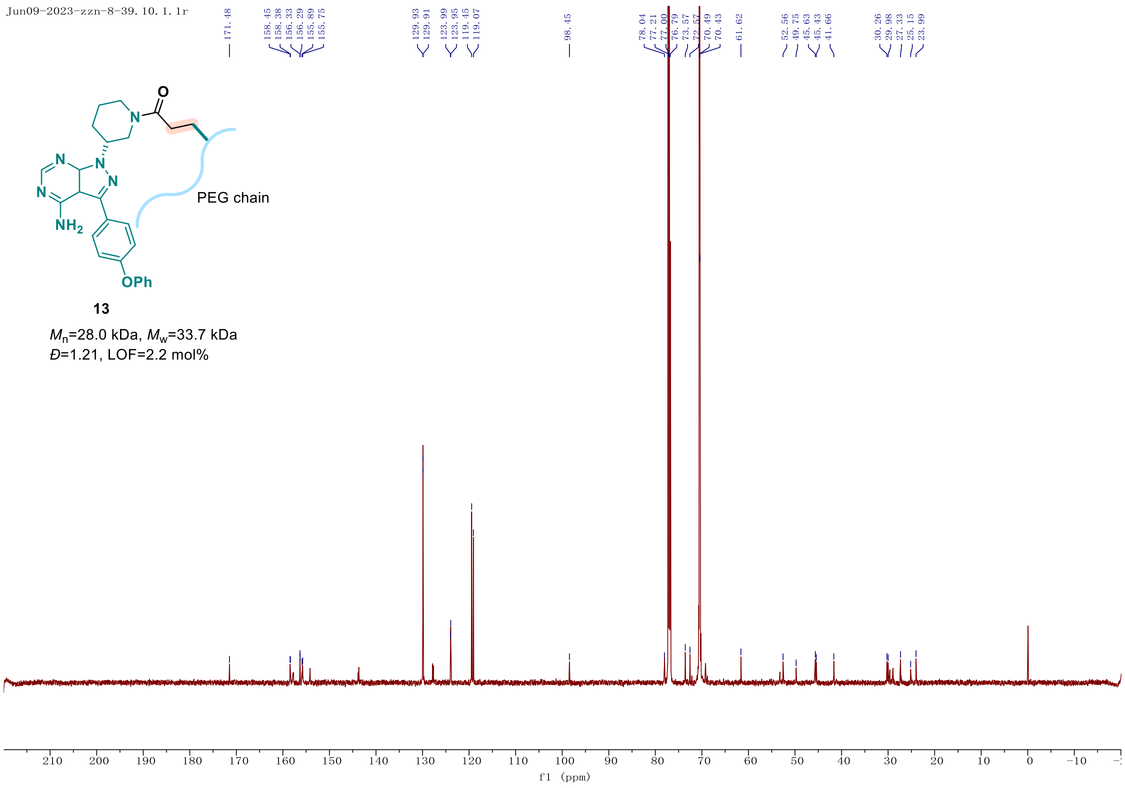

^{1}H NMR (400 MHz, $CDCl_3$) of **PEG 12000-14**

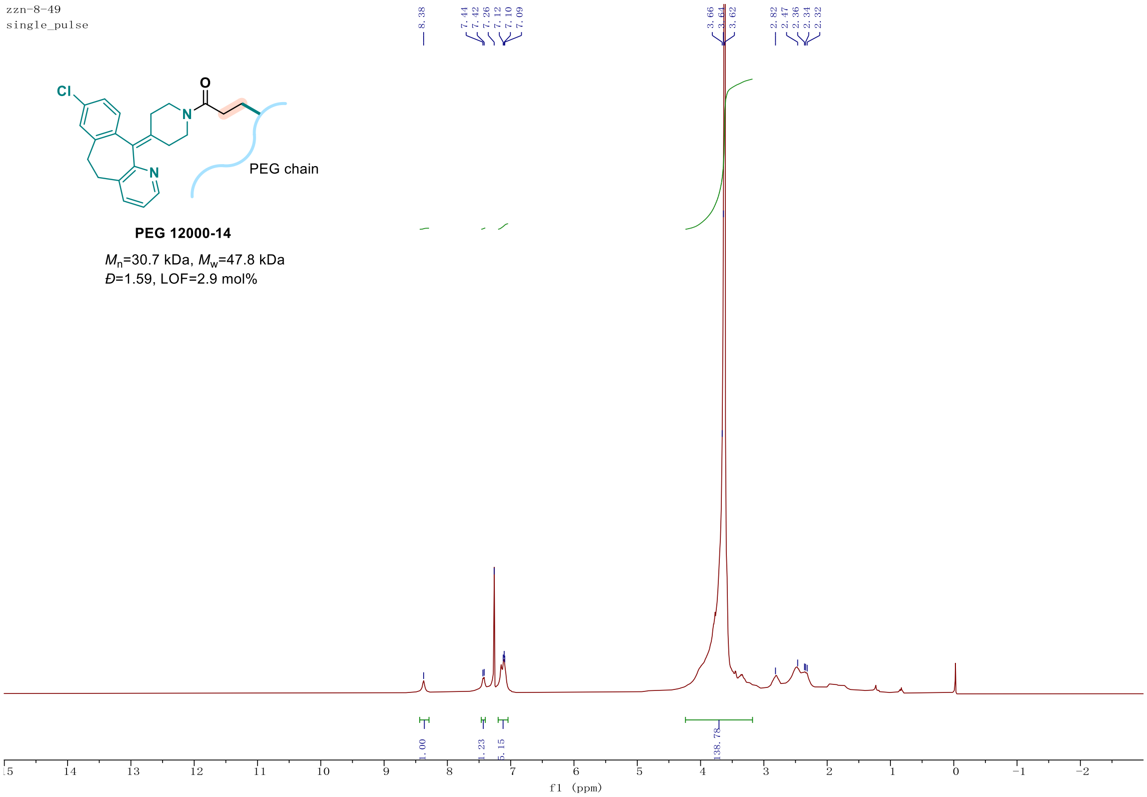

^{13}C NMR (151 MHz, $CDCl_3$) of **PEG 12000-14**

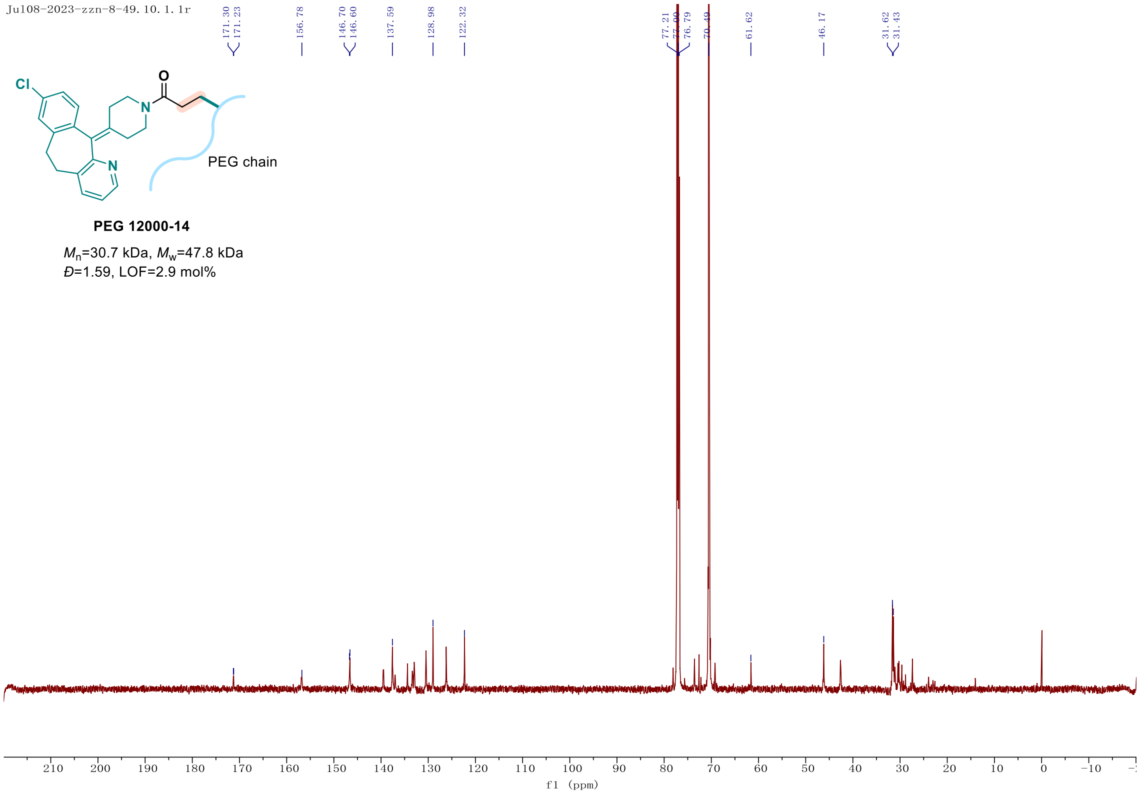

^{1}H NMR (400 MHz, $CDCl_3$) of **PEG 12000-15**

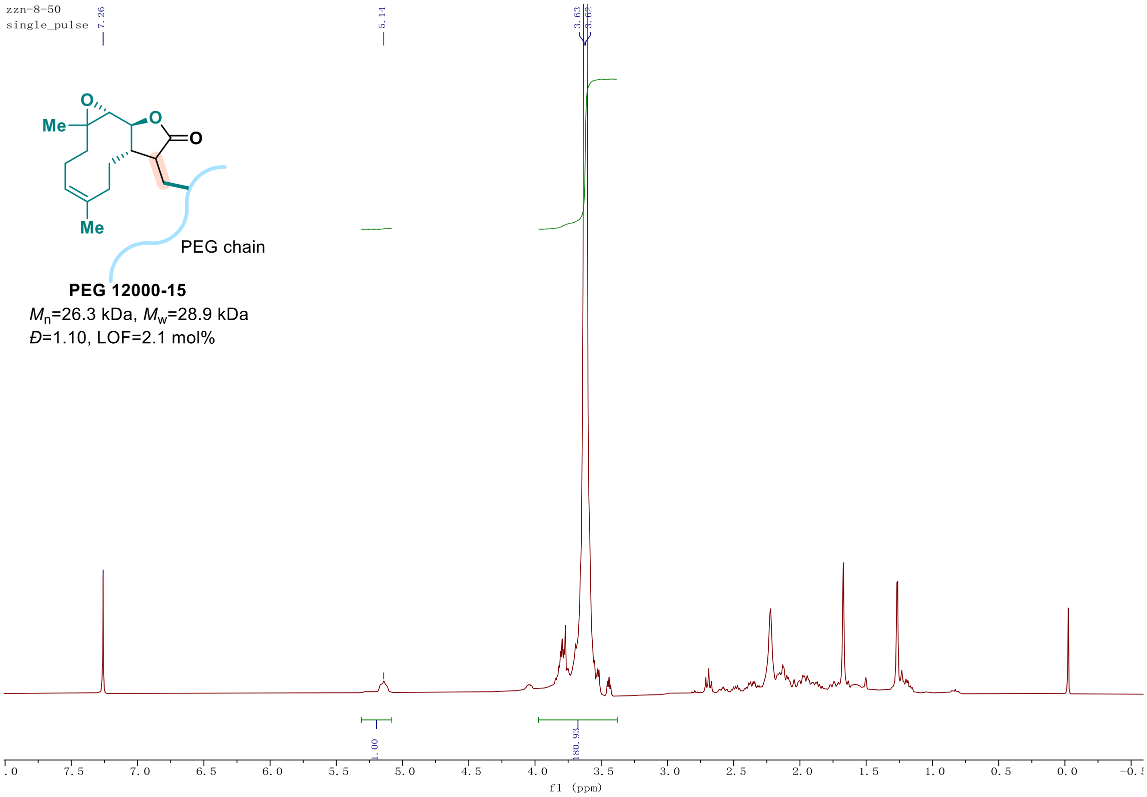

^{13}C NMR (151 MHz, $CDCl_3$) of **PEG 12000-15**

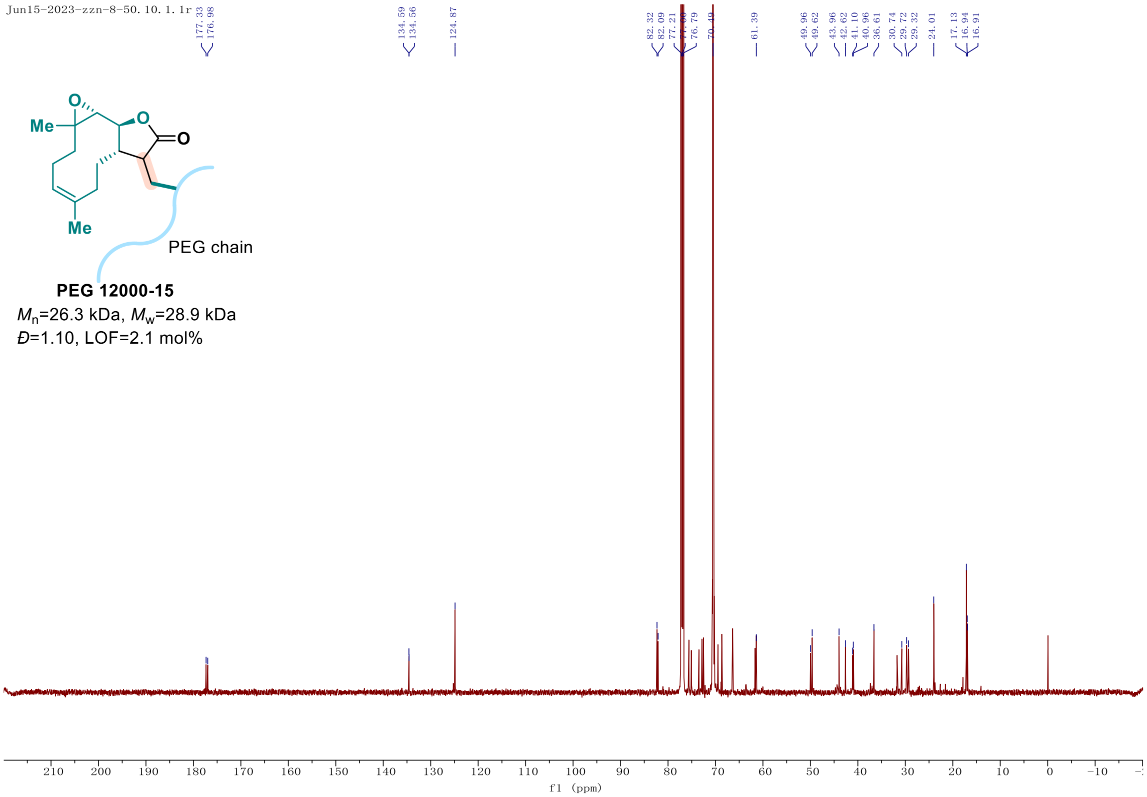

^{1}H NMR (400 MHz, $CDCl_3$) of **PEG 12000-16**

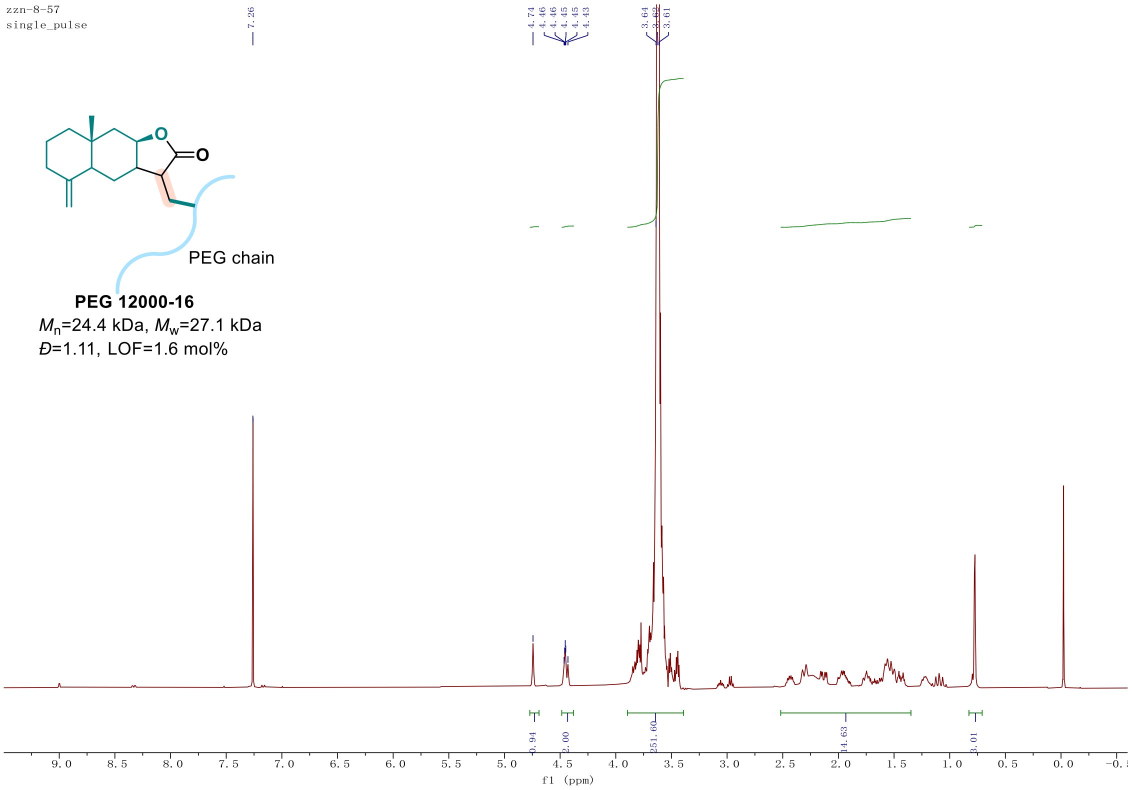

^{13}C NMR (151 MHz, $CDCl_3$) of **PEG 12000-16**

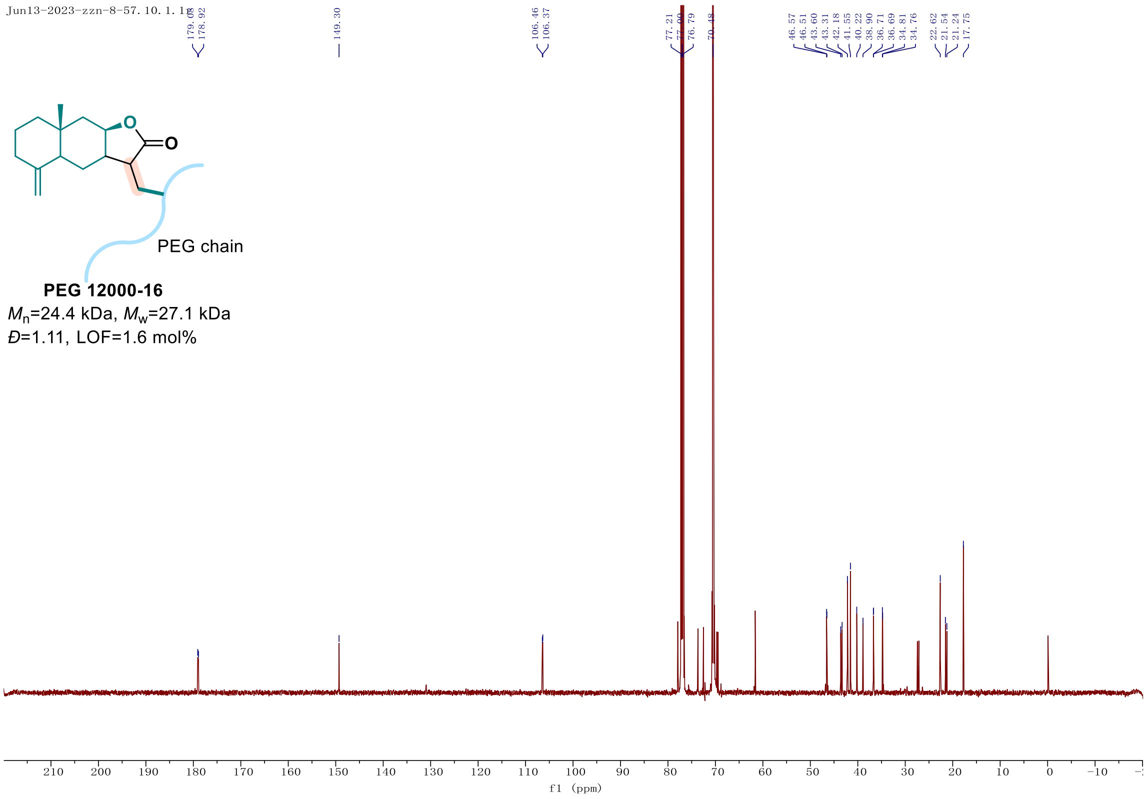

^{1}H NMR (400 MHz, $CDCl_3$) of **PEG 12000-17**

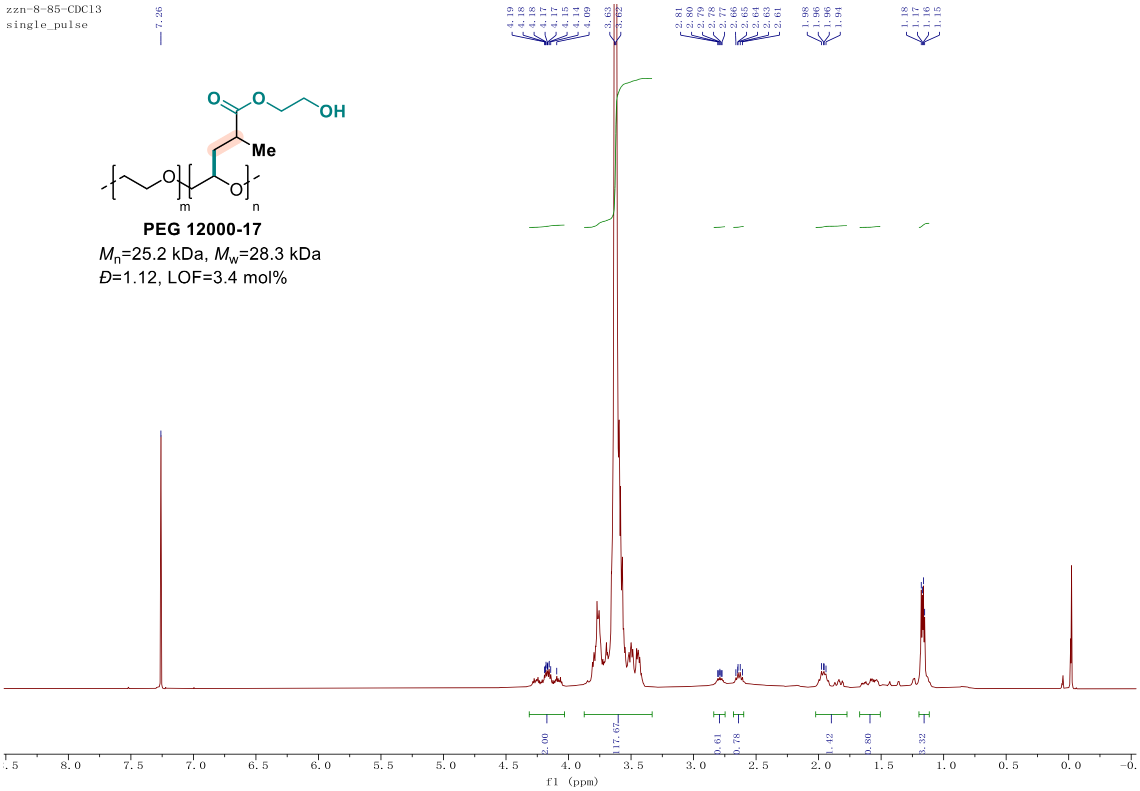

^{13}C NMR (151 MHz, $CDCl_3$) of **PEG 12000-17**

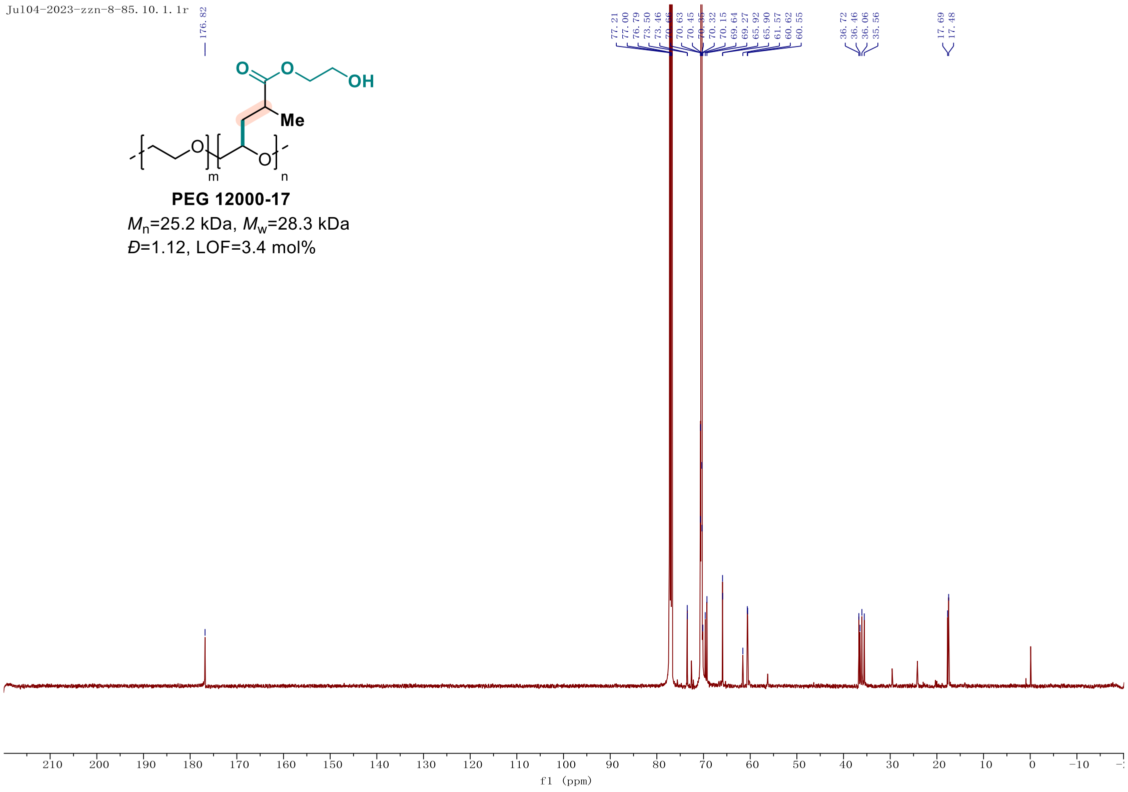

^{1}H NMR (400 MHz, $CDCl_3$) of **PEG 12000-18**

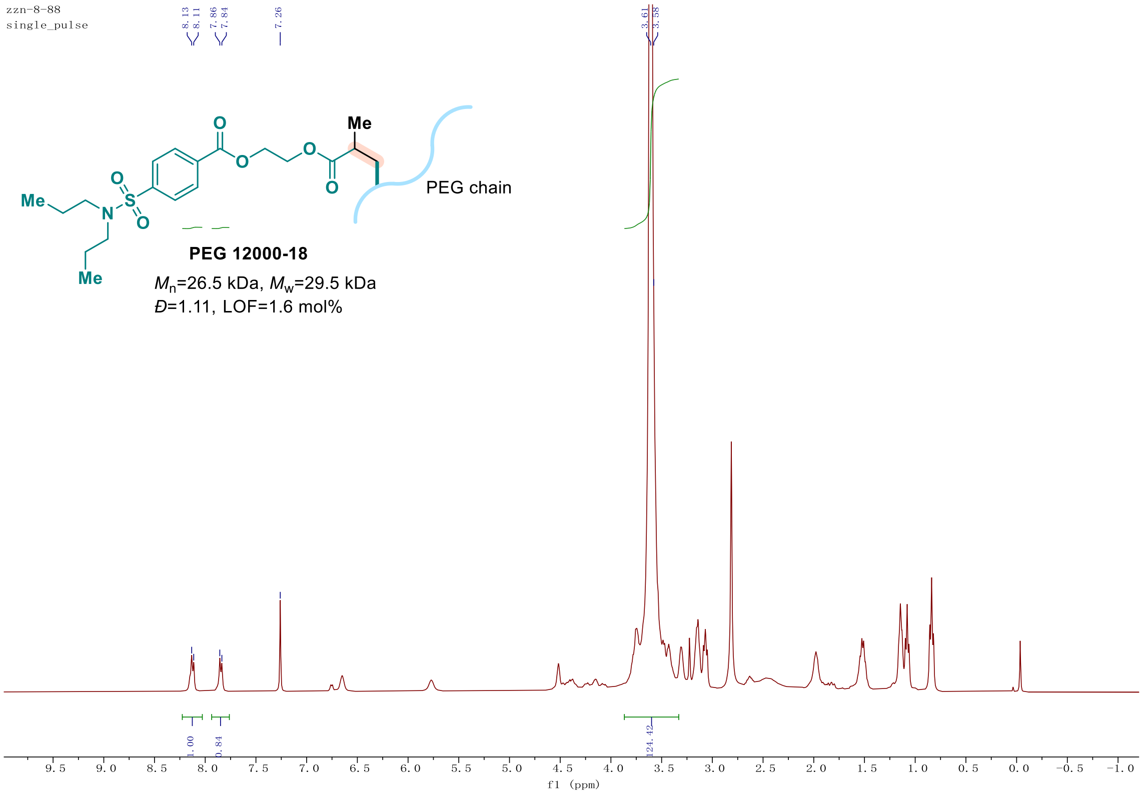

^{13}C NMR (151 MHz, $CDCl_3$) of **PEG 12000-18**

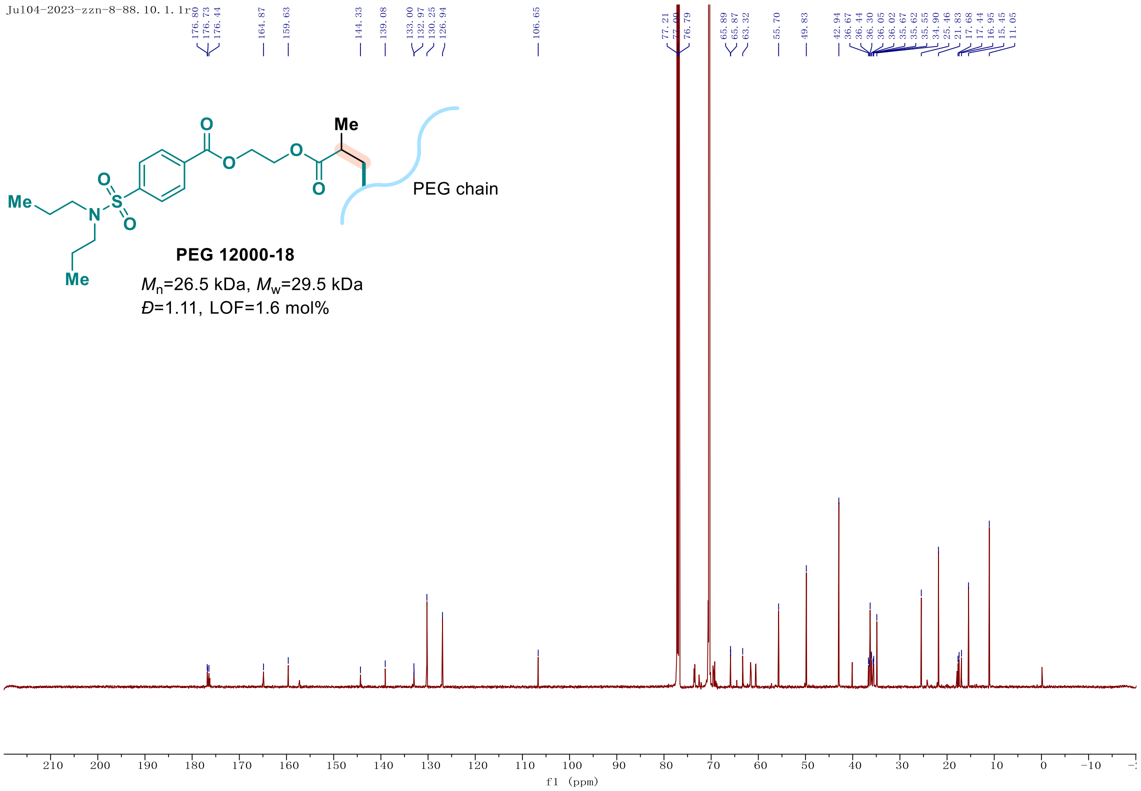

^{1}H NMR (400 MHz, $CDCl_3$) of **PEG 12000-19**

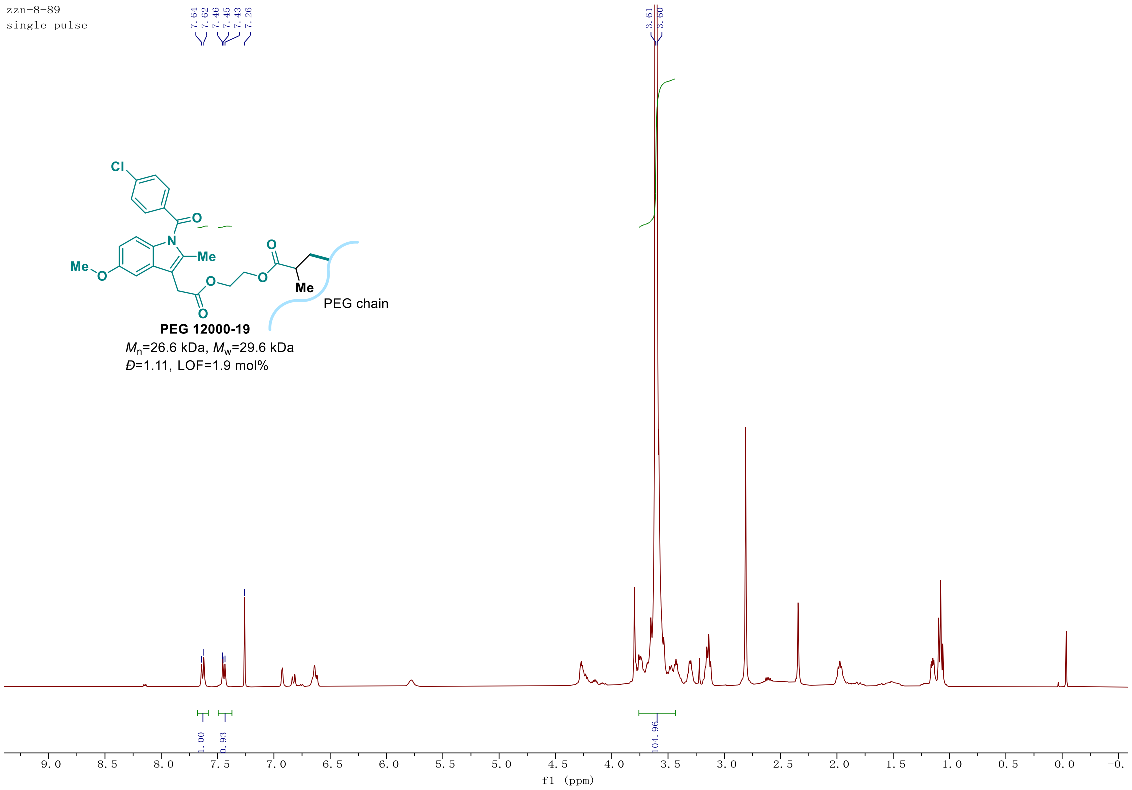

^{13}C NMR (151 MHz, $CDCl_3$) of **PEG 12000-19**

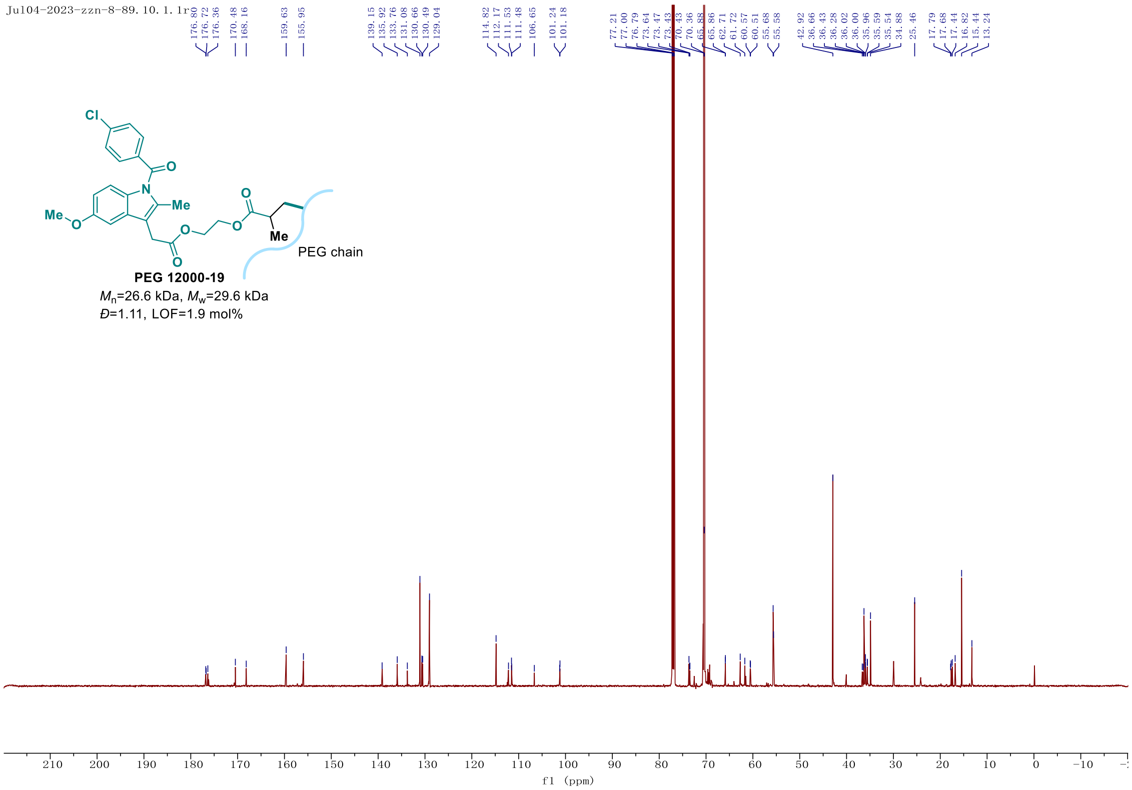

^{1}H NMR (400 MHz, $CDCl_3$) of **PEG 12000-20**

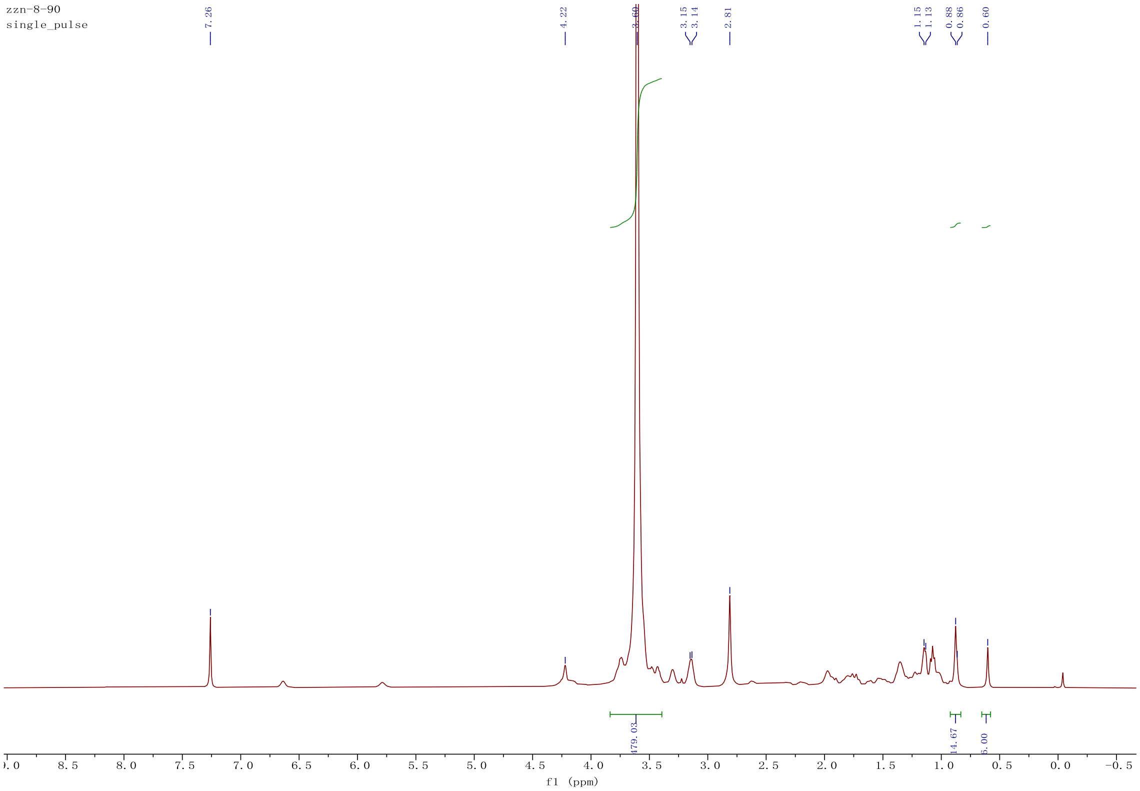

^{13}C NMR (151 MHz, $CDCl_3$) of **PEG 12000-20**

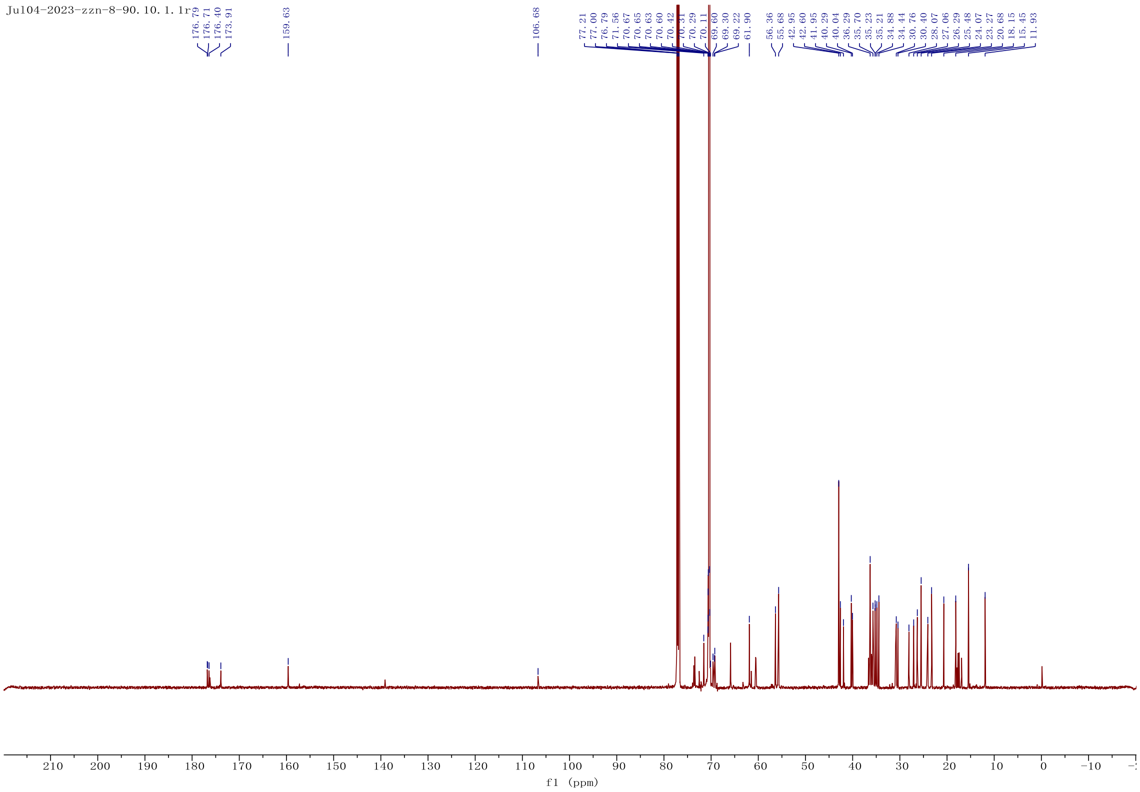

^{1}H NMR (400 MHz, $CDCl_3$) of **PEG 12000-17**

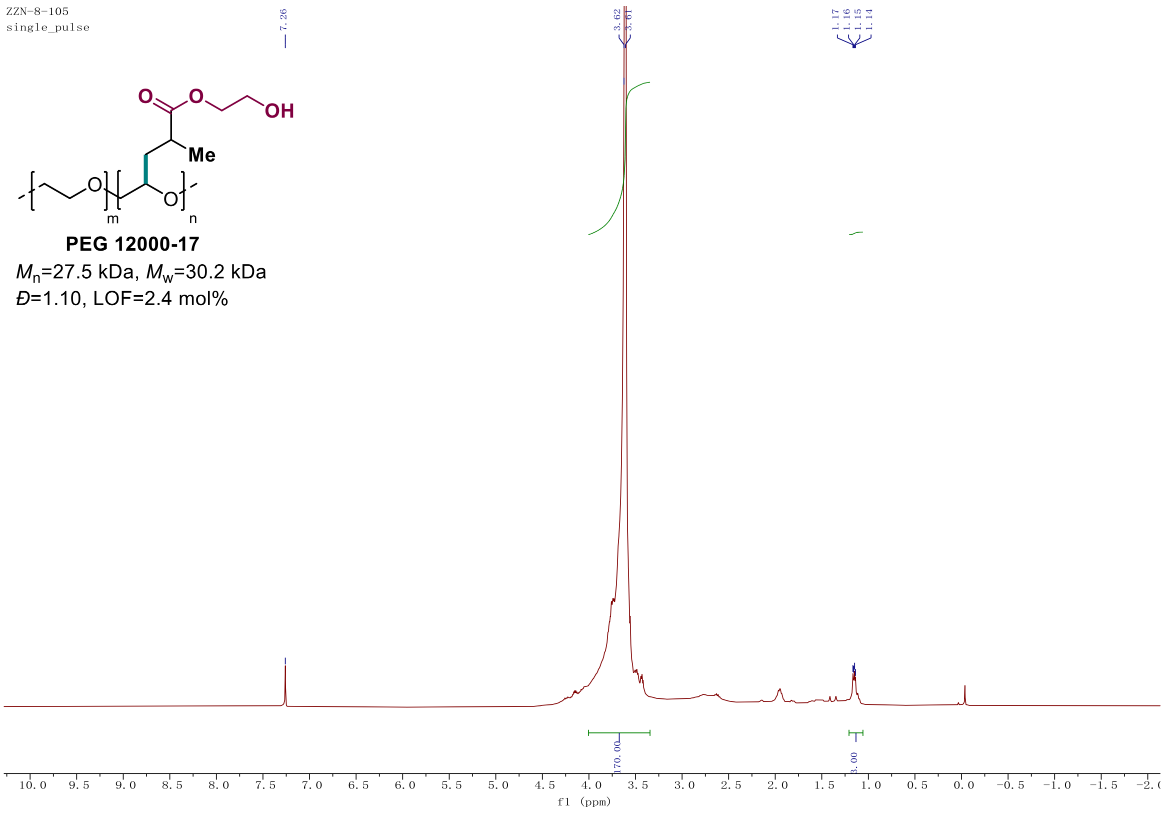

^{1}H NMR (400 MHz, $CDCl_3$) of **PEG 12000-17-11**

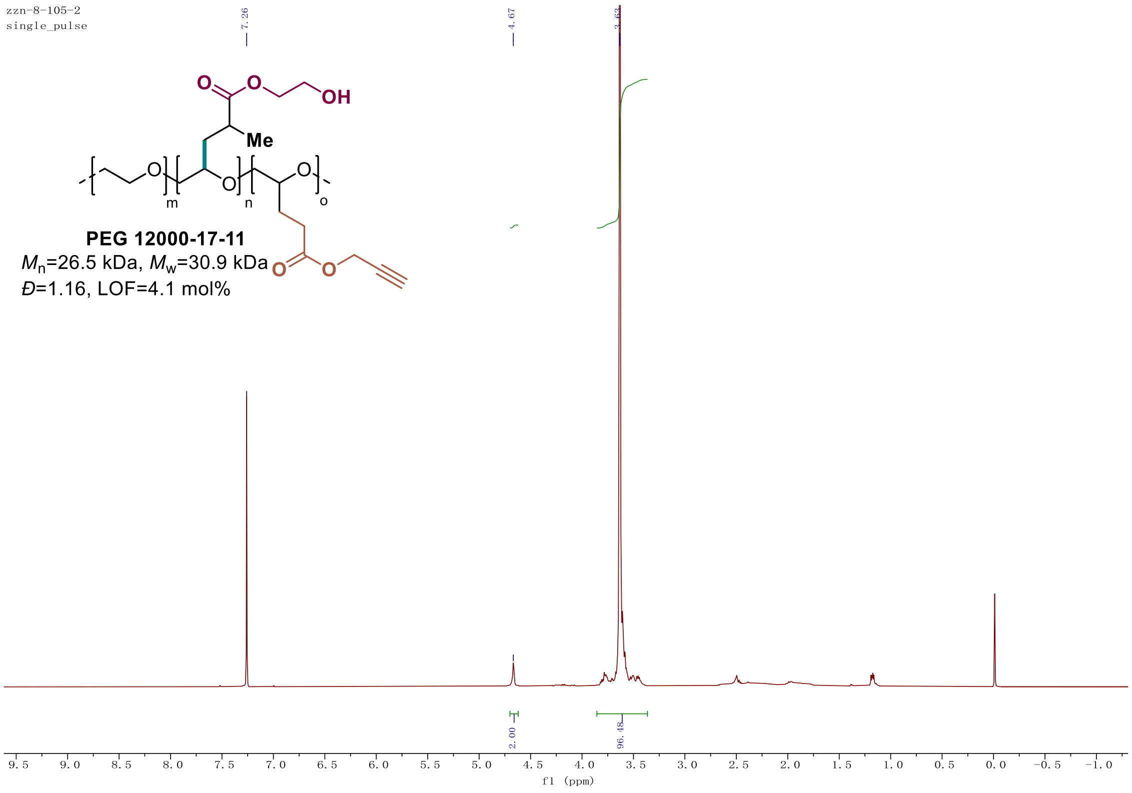

^{1}H NMR (400 MHz, $CDCl_3$) of **PEG 12000-17-11-15**

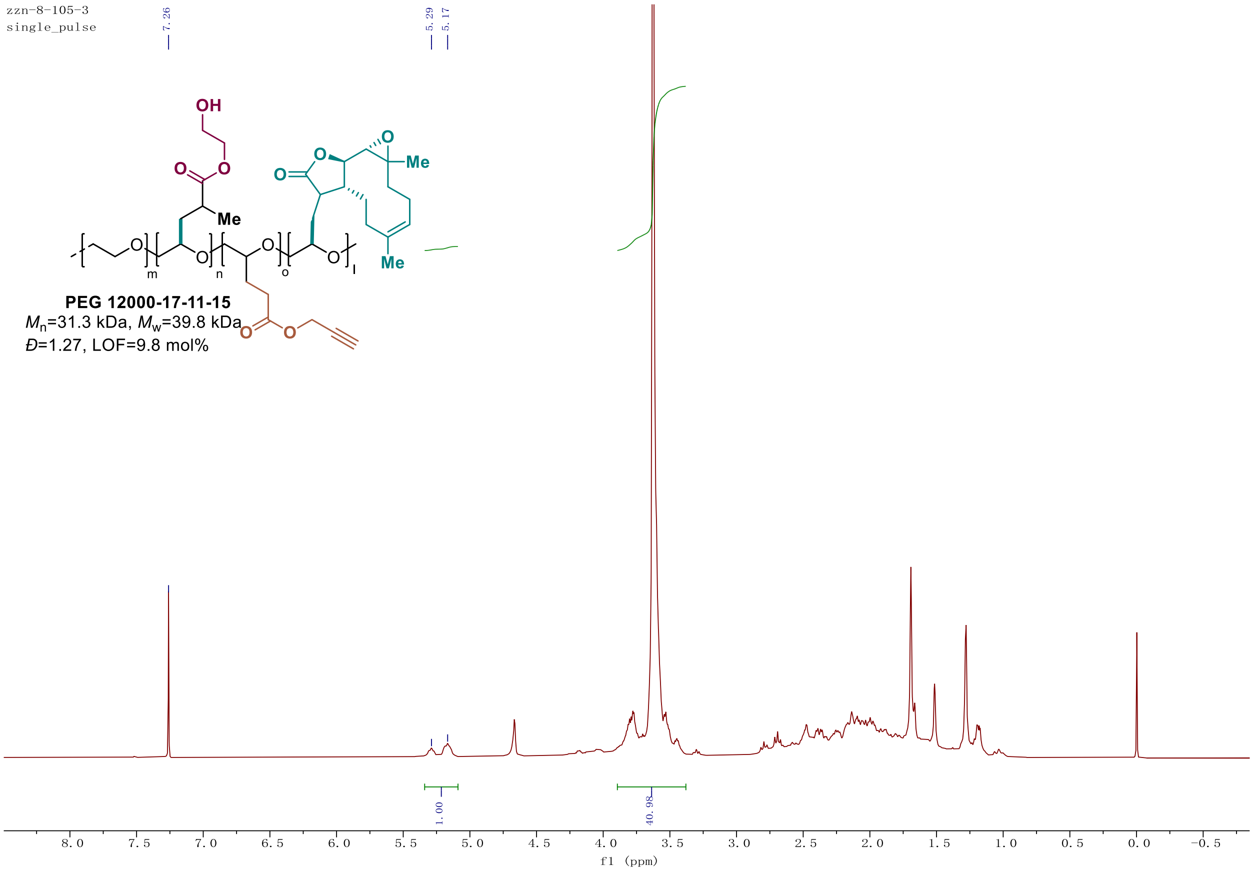

^{1}H NMR (400 MHz, CDCl$_3$) of **MeO-PEG-OMe 2000-17**

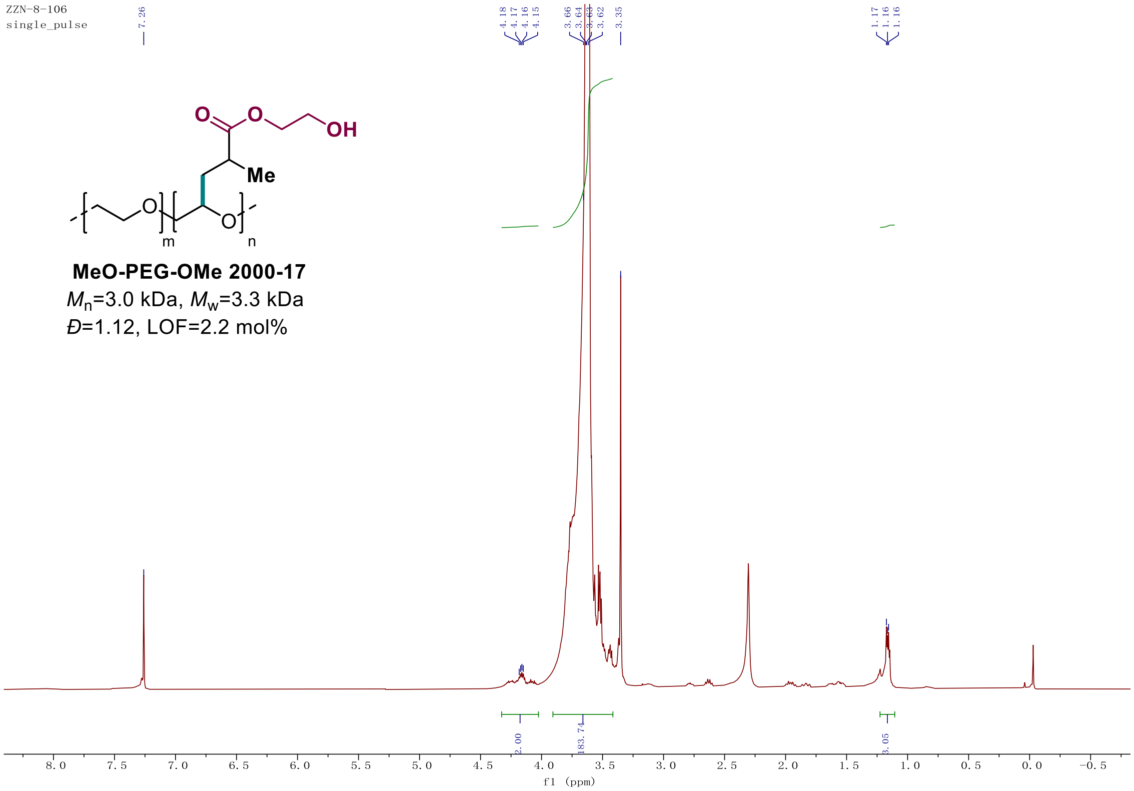

^{13}C NMR (151 MHz, CDCl$_3$) of **MeO-PEG-OMe 2000-17**

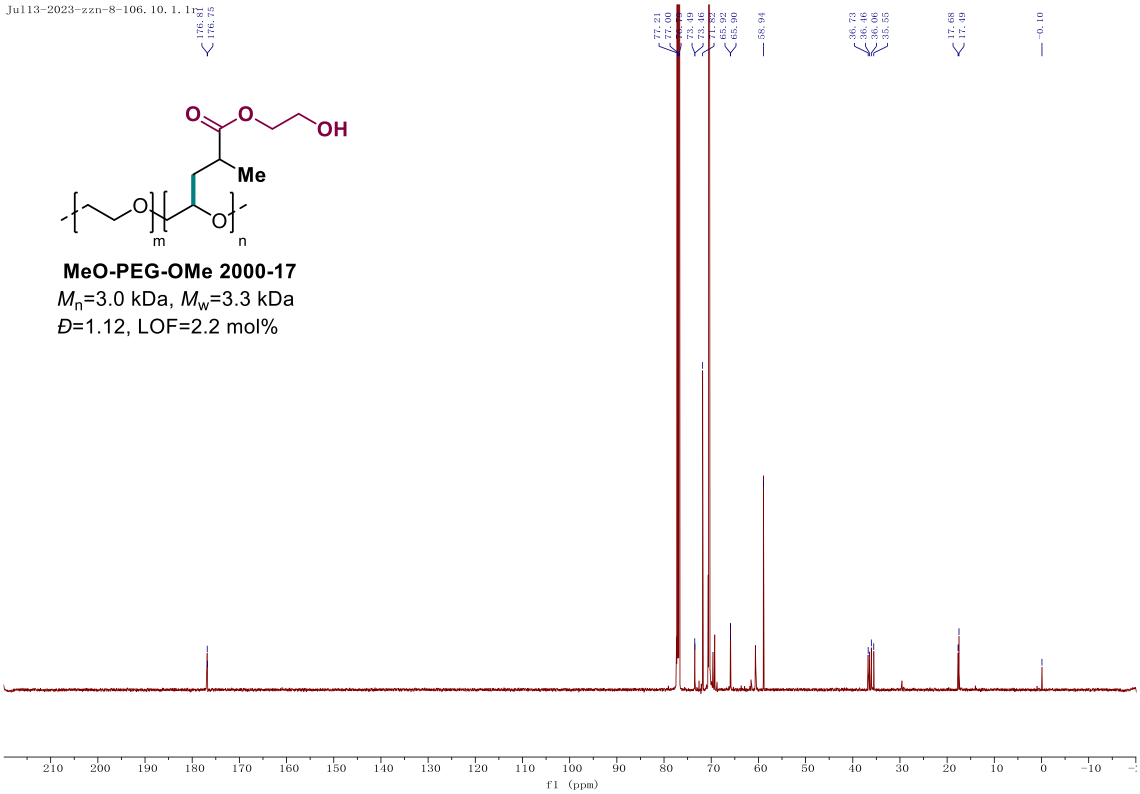

^{1}H NMR (400 MHz, $CDCl_3$) of **MeO-PEG-OMe 2000-21**

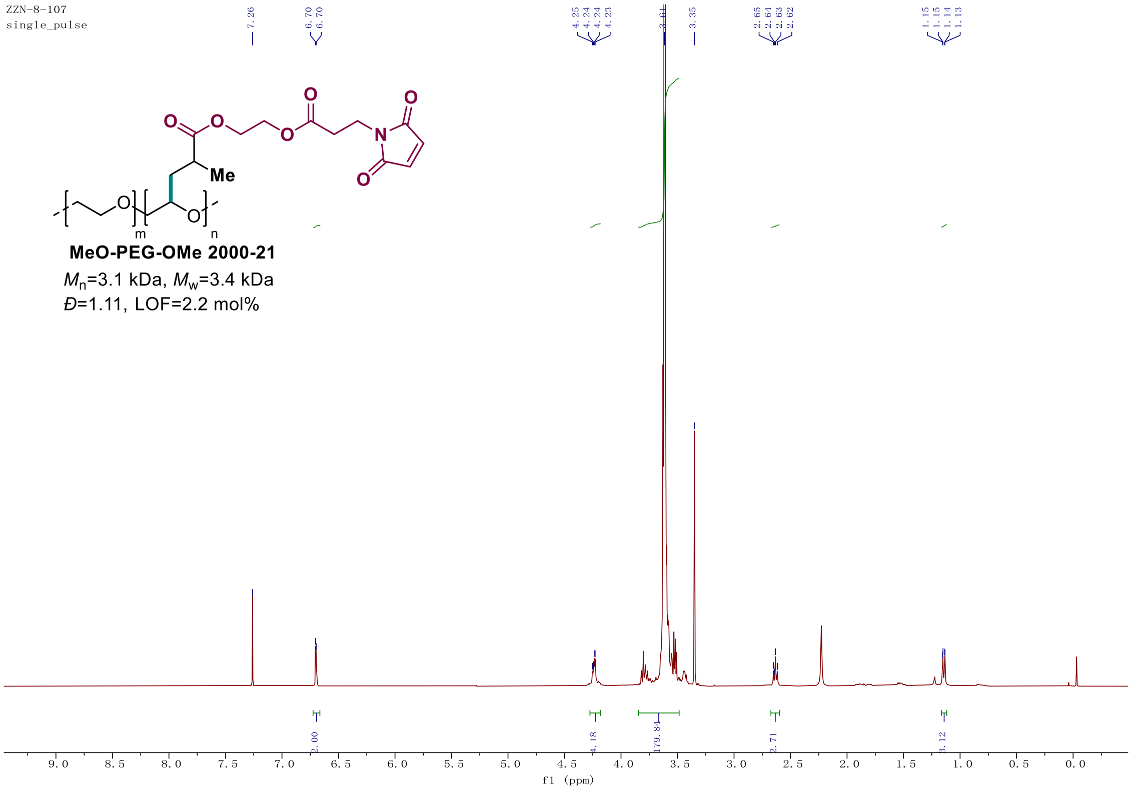

^{13}C NMR (151 MHz, $CDCl_3$) of **MeO-PEG-OMe 2000-21**

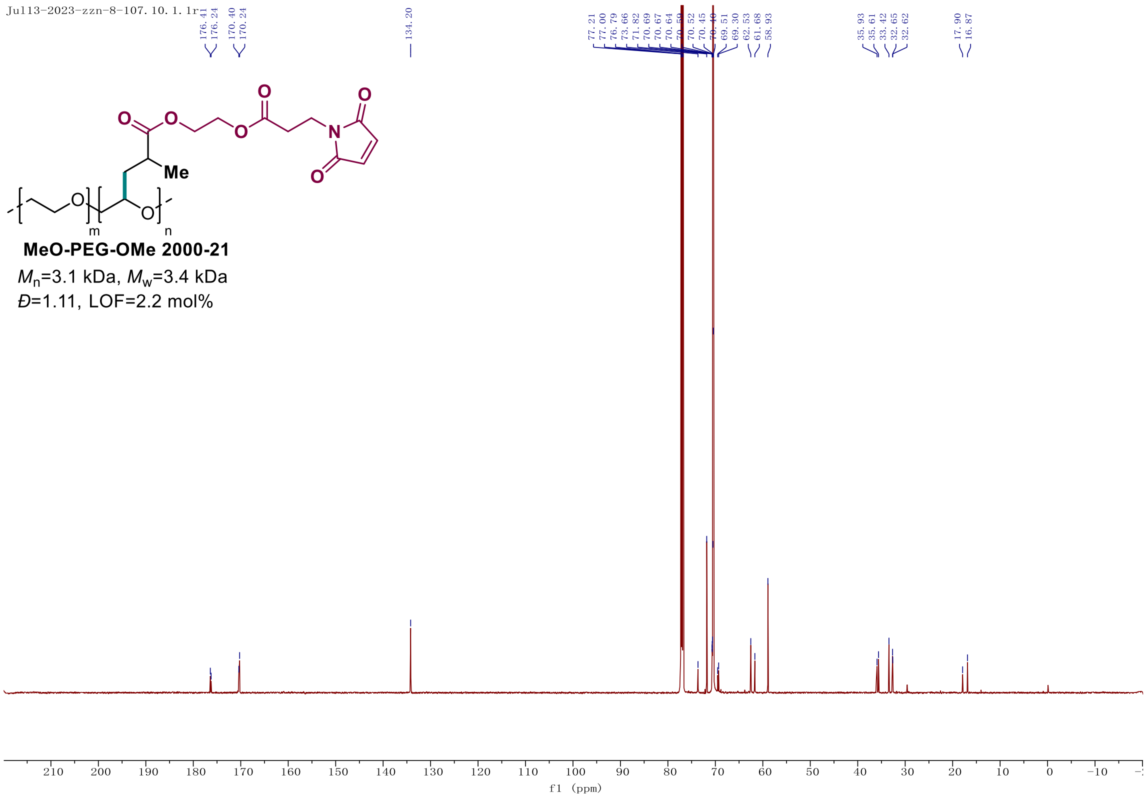

^{1}H NMR (400 MHz, $CDCl_3$) of **DME-1**

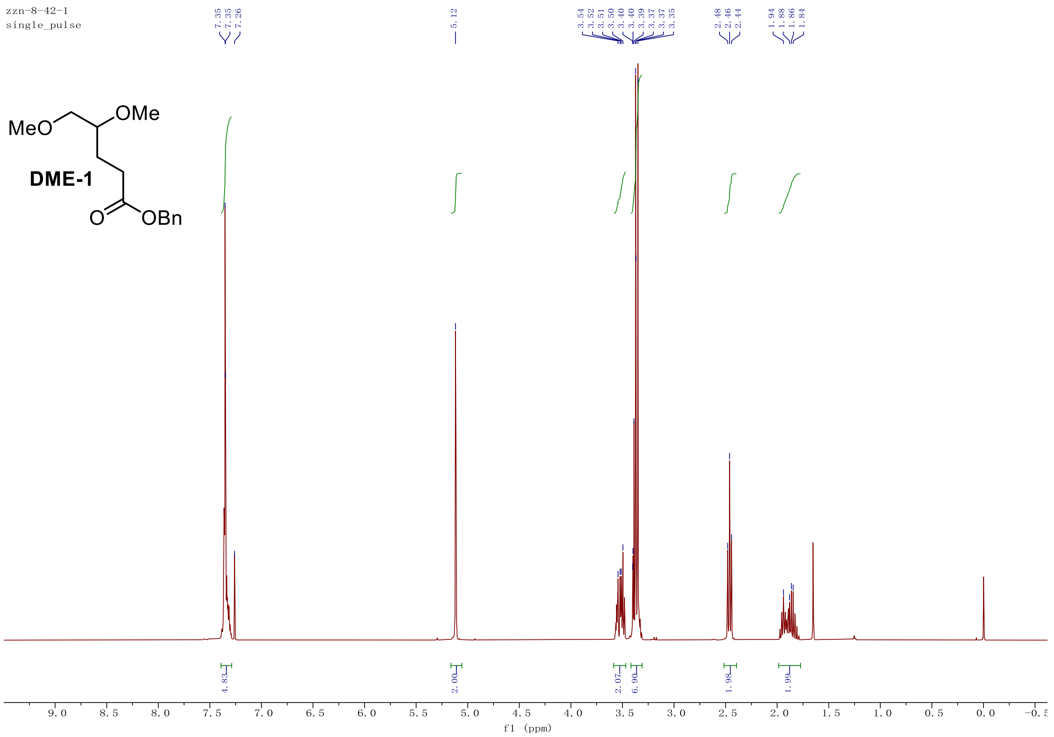

^{13}C NMR (101 MHz, $CDCl_3$) of **DME-1**

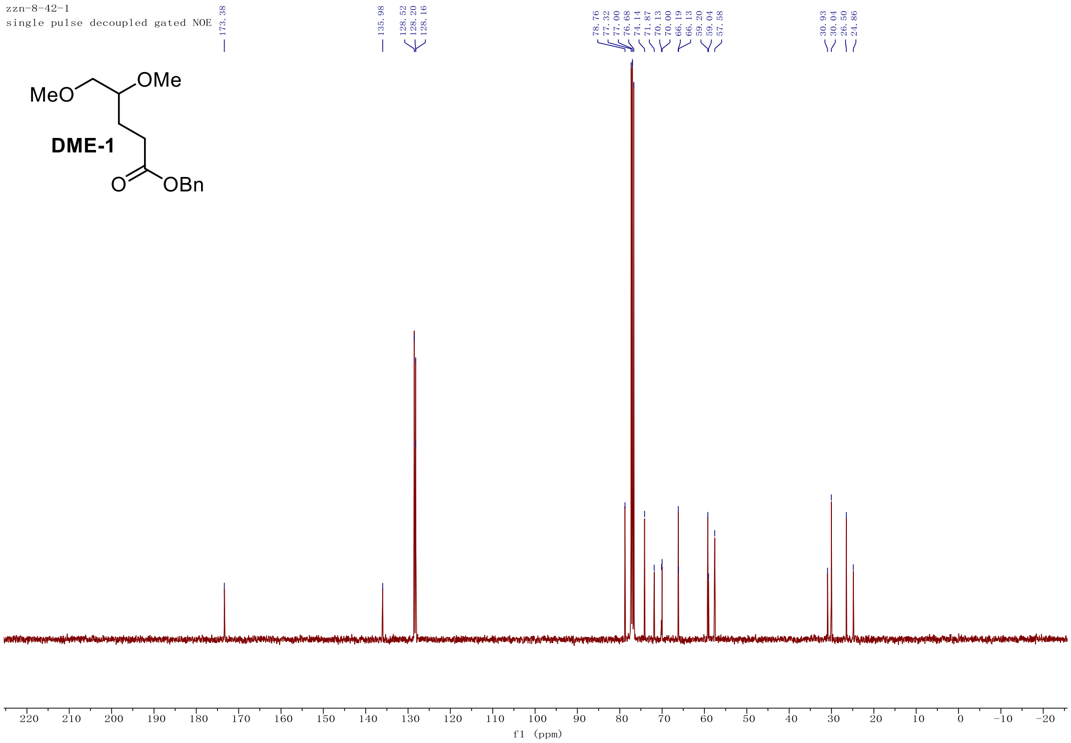

^{1}H NMR (400 MHz, $CDCl_3$) of **18-crown-6-1**

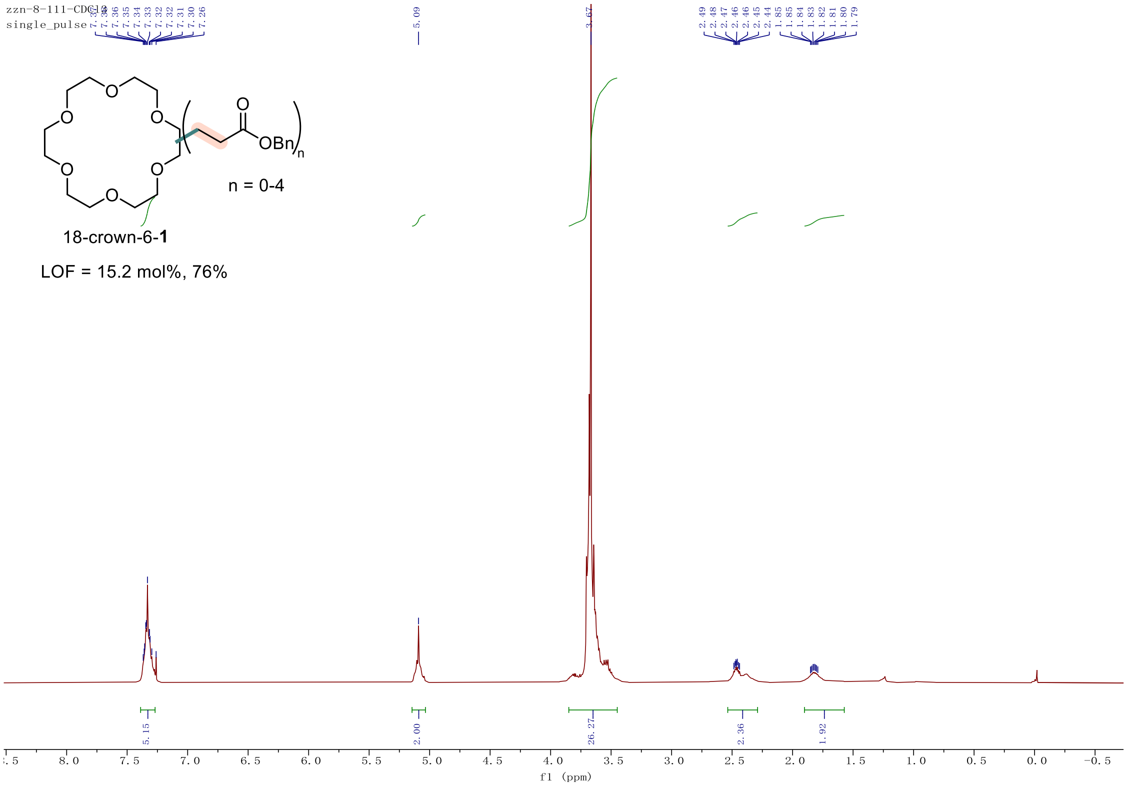

^{13}C NMR (151 MHz, $CDCl_3$) of **18-crown-6-1**

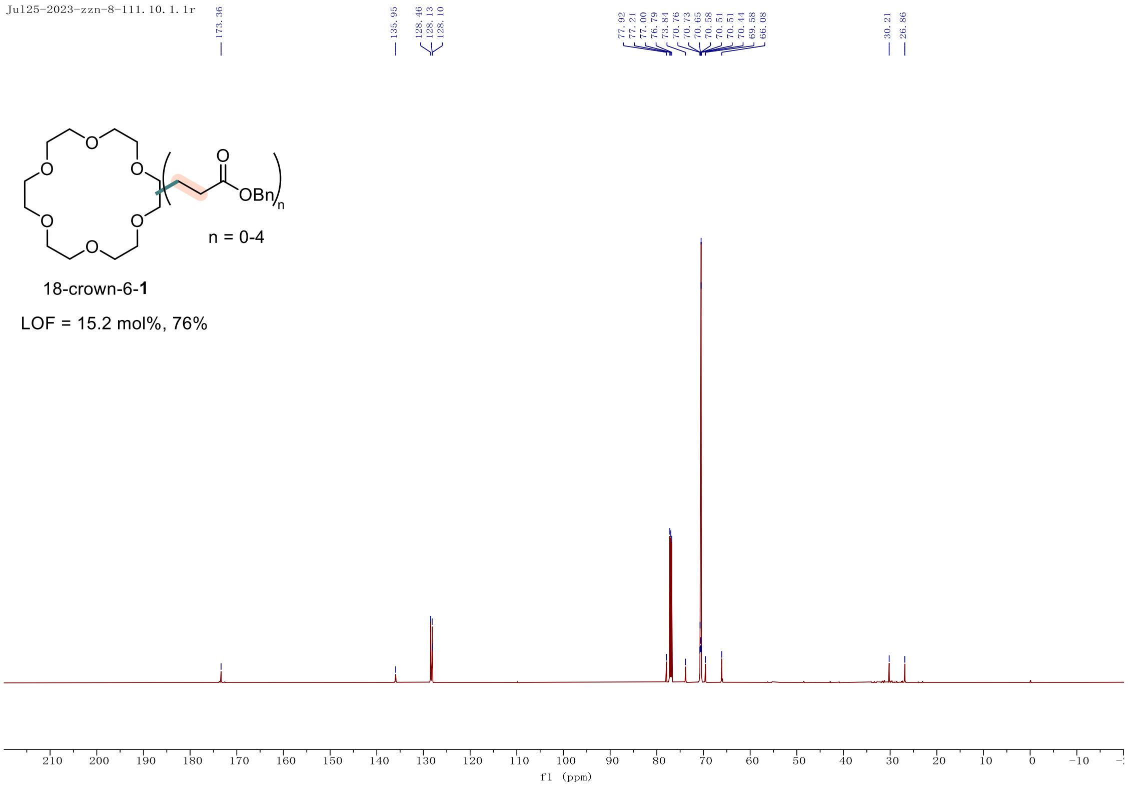

^{1}H NMR (400 MHz, $CDCl_3$) of **18-crown-6-1** (n = 1)

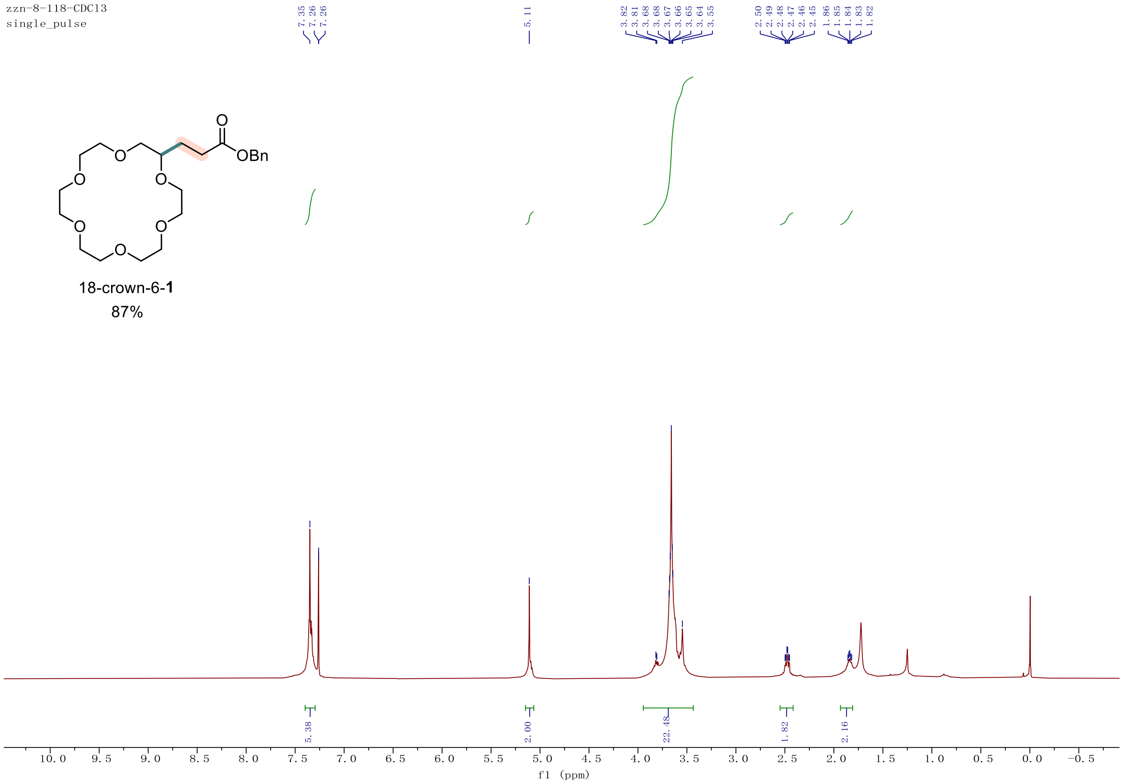

^{13}C NMR (151 MHz, $CDCl_3$) of **18-crown-6-1** (n = 1)

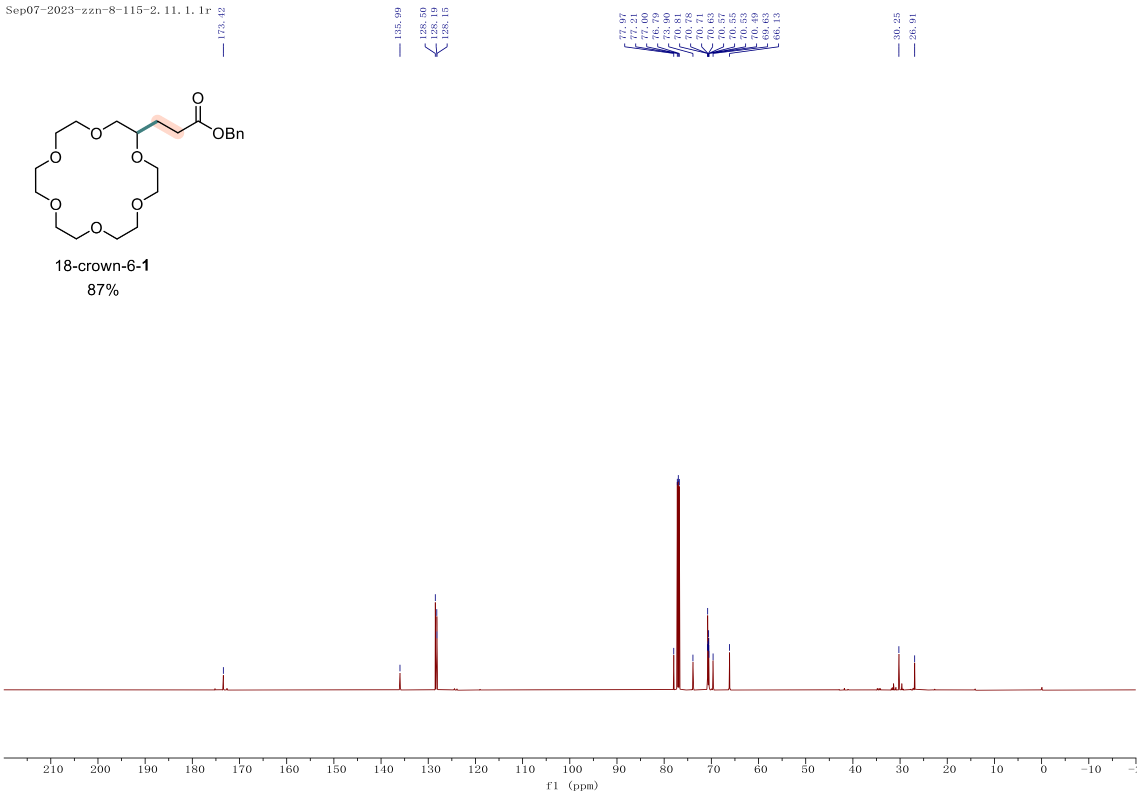

Supplementary References

1. Dong, Y. et al. Photoredox Catalytic Installation of an Alkyl/Aryl Dide Chain and Deuterium into (*S*)-Methyleneoxazolidinone: Synthesis of Enantioenriched α-Deuterated α-Amino Acid Derivatives. *Org. Lett.* **25**, 4745-4749 (2023).

2. Shan, H. et al. Discovery of a Novel Covalent CDK4/6 Inhibitor Based on Palbociclib Scaffold. *Eur. J. Med. Chem.* **219**, 113432 (2021).

3. Zhang, Z. et al. Controllable C–H Alkylation of PEGs via Iron Photocatalysis. *J. Am. Chem. Soc.* **145**, 7612-7620 (2023).

4. Gaussian 09, Revision E.01, Frisch, M. J.; Trucks, G. W.; Schlegel, H. B.; Scuseria, G. E.; Robb, M. A.; Cheeseman, J. R.; Scalmani, G.; Barone, V.; Mennucci, B.; Petersson, G. A.; Nakatsuji, H.; Caricato, M.; Li, X.; Hratchian, H. P.; Izmaylov, A. F.; Bloino, J.; Zheng, G.; Sonnenberg, J. L.; Hada, M.; Ehara, M.; Toyota, K.; Fukuda, R.; Hasegawa, J.; Ishida, M.; Nakajima, T.; Honda, Y.; Kitao, O.; Nakai, H.; Vreven, T.; Montgomery, J. A.; Peralta, Jr., J. E.; Ogliaro, F.; Bearpark, M.; Heyd, J. J.; Brothers, E.; Kudin, K. N.; Staroverov, V. N.; Keith, T.; Kobayashi, R.; Normand, J.; Raghavachari, K.; Rendell, A.; Burant, J. C.; Iyengar, S. S.; Tomasi, J.; Cossi, M.; Rega, N.; Millam, J. M.; Klene, M.; Knox, J. E.; Cross, J. B.; Bakken, V.; Adamo, C.; Jaramillo, J.; Gomperts, R.; Stratmann, R. E.; Yazyev, O.; Austin, A. J.; Cammi, R.; Pomelli, C.; Ochterski, J. W.; Martin, R. L.; Morokuma, K.; Zakrzewski, V. G.; Voth, G. A.; Salvador, P.; Dannenberg, J. J.; Dapprich, S.; Daniels, A. D.; Farkas, O.; Foresman, J. B.; Ortiz, J. V.; Cioslowski, J.; and Fox, D. J. Gaussian, Inc., Wallingford CT, 2013.

5. Becke, A. D. *J. Chem. Phys.* Density-Functional Thermochemistry. III. The Role of Exact Exchange. **98**, 5648-5652 (1993).

6. Lee, C.; Yang, W.; Parr, R. G. Development of the Colle-Salvetti Correlation-Energy Formula into a Functional of the Electron Density. *Phys. Rev. B*, **37**, 785-789 (1988).

7. Grimme, S.; Ehrlich, S.; Goerigk, L. Effect of the Damping Function in Dispersion Corrected Density Functional Theory. *J. Comput. Chem.* **32**, 1456-1465 (2011).

8. Dolg, M.; Wedig, U.; Stoll, H.; Preuss, H. Energy-Adjusted ab Initio Pseudopotentials for the First Row Transition Elements. *J. Chem. Phys.* **86**, 866-872 (1987).

9. Nicklass, A.; Dolg, M.; Stoll, H.; Preuss, H. Ab Initio Energy-Adjusted Pseudopotentials for the Noble Gases Ne Through Xe: Calculation of Atomic Dipole and Quadrupole Polarizabilities. *J. Chem. Phys.* **102**, 8942-8952 (1995).

10. Ditchfield, R.; Hehre, W. J.; Pople, J. A. Self-Consistent Molecular-Orbital Methods. IX. An Extended Gaussian-Type Basis for Molecular-Orbital Studies of Organic Molecules. *J. Chem. Phys.* **54**, 724-728 (1971).

11. Hehre, W. J.; Ditchfield, R.; Pople, J. A. Self-Consistent Molecular Orbital Methods. XII. Further Extensions of Gaussian-Type Basis Sets for Use in Molecular Orbital Studies of Organic Molecules. *J. Chem. Phys.* **56**, 2257-2261 (1972).

12. Hariharan, P. C.; Pople, J. A. The Influence of Polarization Functions on Molecular Orbital Hydrogenation Energies. *Theor. Chim. Acta* **28**, 213-222 (1973).

13. Zhao, Y.; Truhlar, D. G. The M06 Suite of Density Functionals for Main Group Thermochemistry, Thermochemical Kinetics, Noncovalent Interactions, Excited States, and Transition Elements: Two New Functionals and Systematic Testing of Four M06-Class Functionals and 12 Other Functionals. *Theor. Chem. Acc.* 120, 215-241 (2008).

14. Clark, T.; Chandrasekhar, J.; Spitznagel, G. W.; Schleyer, P. Von R. Efficient Diffuse Function-Augmented Basis Sets for Anion Calculations. III. The 3-21+G Basis Set for First-Row Elements, Li–F. *J. Comput. Chem.* **4**, 294-301 (1983).

15. Krishnan, R.; Binkley, J. S.; Seeger, R.; Pople, J. A. Self-Consistent Molecular Orbital Methods. XX. A Basis Set for Correlated Wave Functions. *J. Chem. Phys.* **72**, 650-654 (1980).

16. Marenich, A. V.; Cramer, C. J.; Truhlar, D. G. Universal Solvation Model Based on Solute Electron Density and on a Continuum Model of the Solvent Defined by the Bulk Dielectric Constant and Atomic Surface Tensions. *J. Phys. Chem. B.* **113**, 6378-6396 (2009).
